# Cosmic kidney disease: an integrated pan-omic, physiological and morphological study into spaceflight-induced renal dysfunction

**DOI:** 10.1038/s41467-024-49212-1

**Published:** 2024-06-11

**Authors:** Keith Siew, Kevin A. Nestler, Charlotte Nelson, Viola D’Ambrosio, Chutong Zhong, Zhongwang Li, Alessandra Grillo, Elizabeth R. Wan, Vaksha Patel, Eliah Overbey, JangKeun Kim, Sanghee Yun, Michael B. Vaughan, Chris Cheshire, Laura Cubitt, Jessica Broni-Tabi, Maneera Yousef Al-Jaber, Valery Boyko, Cem Meydan, Peter Barker, Shehbeel Arif, Fatemeh Afsari, Noah Allen, Mohammed Al-Maadheed, Selin Altinok, Nourdine Bah, Samuel Border, Amanda L. Brown, Keith Burling, Margareth Cheng-Campbell, Lorianna M. Colón, Lovorka Degoricija, Nichola Figg, Rebecca Finch, Jonathan Foox, Pouya Faridi, Alison French, Samrawit Gebre, Peter Gordon, Nadia Houerbi, Hossein Valipour Kahrood, Frederico C. Kiffer, Aleksandra S. Klosinska, Angela Kubik, Han-Chung Lee, Yinghui Li, Nicholas Lucarelli, Anthony L. Marullo, Irina Matei, Colleen M. McCann, Sayat Mimar, Ahmed Naglah, Jérôme Nicod, Kevin M. O’Shaughnessy, Lorraine Christine De Oliveira, Leah Oswalt, Laura Ioana Patras, San-huei Lai Polo, María Rodríguez-Lopez, Candice Roufosse, Omid Sadeghi-Alavijeh, Rebekah Sanchez-Hodge, Anindya S. Paul, Ralf Bernd Schittenhelm, Annalise Schweickart, Ryan T. Scott, Terry Chin Choy Lim Kam Sian, Willian A. da Silveira, Hubert Slawinski, Daniel Snell, Julio Sosa, Amanda M. Saravia-Butler, Marshall Tabetah, Erwin Tanuwidjaya, Simon Walker-Samuel, Xiaoping Yang, Yasmin, Haijian Zhang, Jasminka Godovac-Zimmermann, Pinaki Sarder, Lauren M. Sanders, Sylvain V. Costes, Robert A. A. Campbell, Fathi Karouia, Vidya Mohamed-Alis, Samuel Rodriques, Steven Lynham, Joel Ricky Steele, Sergio Baranzini, Hossein Fazelinia, Zhongquan Dai, Akira Uruno, Dai Shiba, Masayuki Yamamoto, Eduardo A.C.Almeida, Elizabeth Blaber, Jonathan C. Schisler, Amelia J. Eisch, Masafumi Muratani, Sara R. Zwart, Scott M. Smith, Jonathan M. Galazka, Christopher E. Mason, Afshin Beheshti, Stephen B. Walsh

**Affiliations:** 1 London Tubular Centre, Department of Renal Medicine, University College London, London, UK; 2 The Institute for Biomedical Sciences (IBS), The George Washington University, Washington, DC USA; 3 Weill Institute for Neurosciences, Department of Neurology, University of California San Francisco, San Francisco, CA USA; 4 Department of Experimental and Translational Medicine, Università Cattolica del Sacro Cuore di Roma, Rome, Italy; 5 Centre for Advanced Biomedical Imaging, University College London, London, UK; 6 Centre for Computational Medicine, University College London, London, UK; 7 Department of Renal Medicine, University College London, London, UK; 8 Institute for Computational Biomedicine, Weill Cornell Medical College, New York, NY USA; 9 University of Pennsylvania Perelman School of Medicine, Philadelphia, PA USA; 10 Department of Anesthesiology and Critical Care Medicine, Children’s Hospital of Philadelphia, Philadelphia, PA USA; 11 School of Medicine, College of Medicine and Health, University College Cork, Cork, Ireland; 12 Tissue Engineering and Biomaterials Group, Ghent University, Ghent, Belgium; 13 Center for Medical Genetics, Department of Biomolecular Medicine, Ghent University, Ghent, Belgium; 14 Bioinformatics and Computational Biology Laboratory, The Francis Crick Institute, London, UK; 15 Applied Biotechnology Laboratory, The Francis Crick Institute, London, UK; 16 Sainsbury Wellcome Centre for Neural Circuits and Behaviour, University College London, London, UK; 17 Anti-Doping Laboratory Qatar, Doha, Qatar; 18 Space Biosciences Division, NASA Ames Research Center, Moffett Field, CA USA; 19 MRC MDU Mouse Biochemistry Laboratory, University of Cambridge, Cambridge, UK; 20 Center for Data Driven Discovery in Biomedicine, Children’s Hospital of Philadelphia, Philadelphia, PA USA; 21 Division of Neurosurgery, Children’s Hospital of Philadelphia, Philadelphia, PA USA; 22 Department of Medicine—Nephrology & Intelligent Critical Care Center, University of Florida, Gainesville, FL USA; 23 Department of Biomedical Engineering, Rensselaer Polytechnic Institute, Troy, NY USA; 24 Centre of Metabolism and Inflammation, University College London, London, UK; 25 School of Medicine, The University of North Carolina at Chapel Hill, Chapel Hill, NC USA; 26 Pharmacology, The University of North Carolina at Chapel Hill, Chapel Hill, NC USA; 27 Blue Marble Space Institute of Science, Seattle, WA USA; 28 Department of Anesthesiology and Critical Care Medicine, Children’s Hospital of Philadelphia Research Institute, Philadelphia, PA USA; 29 KBR, Space Biosciences Division, NASA Ames Research Center, Moffett Field, CA USA; 30 Department of Medicine, University of Cambridge, Cambridge, UK; 31 School of Health, Science and Wellbeing, Staffordshire University, Stoke-on-Trent, UK; 32 Department of Physiology and Biophysics, Weill Cornell Medical College, New York, NY USA; 33 The HRH Prince Alwaleed Bin Talal Bin Abdulaziz Alsaud Institute for Computational Biomedicine, Weill Cornell Medical College, New York, NY USA; 34 Monash Proteomics and Metabolomics Platform, Monash Biomedicine Discovery Institute, Monash University, Clayton, VIC Australia; 35 Physiology, Biophysics & Systems Biology, Weill Cornell Medical College, New York, NY USA; 36 Monash Bioinformatics Platform, Monash Biomedicine Discovery Institute, Monash University, Clayton, VIC Australia; 37 Division of Experimental Medicine & Immunotherapeutics (EMIT), Department of Medicine, University of Cambridge, Cambridge, UK; 38 State Key Laboratory of Space Medicine Fundamentals and Application, China Astronaut Research and Training Center, Beijing, China; 39 Cornell Center for Immunology, Cornell University, Ithaca, NY USA; 40 Children’s Cancer and Blood Foundation Laboratories, Departments of Pediatrics and Cell and Developmental Biology, Drukier Institute for Children’s Health, Meyer Cancer Center, Weill Cornell Medical College, New York, NY USA; 41 Advanced Sequencing Facility, The Francis Crick Institute, London, UK; 42 Federal University of São Paulo (UNIFESP), São Paulo, Brazil; 43 Pediatrics, Weill Cornell Medical College, New York, NY USA; 44 Department of Immunology and Inflammation, Imperial College London, London, UK; 45 Englander Institute for Precision Medicine, Weill Cornell Medical College, New York, NY USA; 46 International Space University, 67400 Illkirch-Graffenstaden, France; 47 University Health Network, Toronto, ON Canada; 48 Department of Agricultural and Biological Engineering, Purdue University, West Lafayette, IN USA; 49 Proteomics, King’s College London, London, UK; 50 Department of Medicine-Quantitative Health Section, University of Florida, Gainesville, FL USA; 51 Departments of Biomedical Engineering and Electrical and Computer Engineering, University of Florida, Gainesville, FL USA; 52 Space Research Within Reach, San Francisco, CA USA; 53 Center for Space Medicine, Baylor College of Medicine, Houston, TX USA; 54 Department of Biomedical and Health Informatics, Children’s Hospital of Philadelphia Research Institute, Philadelphia, PA USA; 55 Department of Integrative Genomics, Tohoku Medical Megabank Organization, Tohoku University, Sendai, Miyagi, Japan; 56 Mouse Epigenetics Project, ISS/Kibo experiment, Japan Aerospace Exploration Agency (JAXA), Tsukuba, Ibaraki Japan; 57 JEM Utilization Center, Human Spaceflight Technology Directorate, Japan Aerospace Exploration Agency (JAXA), Tsukuba, Ibaraki Japan; 58 Department of Medical Biochemistry, Graduate School of Medicine, Tohoku University, Sendai, Miyagi Japan; 59 Center for Biotechnology & Interdisciplinary Studies, Rensselaer Polytechnic Institute, Troy, NY USA; 60 Stanley Center for Psychiatric Research, Massachusetts Institute of Technology and Harvard University, Cambridge, MA USA; 61 Department of Neuroscience, University of Pennsylvania Perelman School of Medicine, Philadelphia, PA USA; 62 Institute of Medicine, University of Tsukuba, Tsukuba, Ibaraki Japan; 63 Department of Preventative Medicine and Community Health, University of Texas Medical Branch, Galveston, TX USA; 64 NASA Johnson Space Center, Houston, TX USA; 65 The WorldQuant Initiative for Quantitative Prediction, Weill Cornell Medical College, New York, NY USA; 66 The Feil Family Brain and Mind Research Institute, Weill Cornell Medical College, New York, NY USA; 67 Broad Institute, Cambridge, MA USA; 68 Space Biosciences Division, Universities Space Research Association (USRA), Washington, DC USA

**Keywords:** Nephrons, Renal calculi, Thrombotic microangiopathies, Sequencing, Mass spectrometry

## Abstract

Missions into Deep Space are planned this decade. Yet the health consequences of exposure to microgravity and galactic cosmic radiation (GCR) over years-long missions on indispensable visceral organs such as the kidney are largely unexplored. We performed biomolecular (epigenomic, transcriptomic, proteomic, epiproteomic, metabolomic, metagenomic), clinical chemistry (electrolytes, endocrinology, biochemistry) and morphometry (histology, 3D imaging, miRNA-ISH, tissue weights) analyses using samples and datasets available from 11 spaceflight-exposed mouse and 5 human, 1 simulated microgravity rat and 4 simulated GCR-exposed mouse missions. We found that spaceflight induces: 1) renal transporter dephosphorylation which may indicate astronauts’ increased risk of nephrolithiasis is in part a primary renal phenomenon rather than solely a secondary consequence of bone loss; 2) remodelling of the nephron that results in expansion of distal convoluted tubule size but loss of overall tubule density; 3) renal damage and dysfunction when exposed to a Mars roundtrip dose-equivalent of simulated GCR.

## Abstract (web summary)

Siew et al. using multi-omic, physiological & imaging approaches have demonstrated that spaceflight causes kidney remodelling, suggesting a contribution to kidney stone formation, & that space radiation causes kidney damage & early signs of dysfunction.

## Introduction

The renewed zeitgeist for space travel, ushered in by the advent of commercial spaceflight and tourism, has spurred on state-funded agencies to embark on even more ambitious exploratory ‘Deep Space’ missions. The first of these, namely the Artemis Program, Lunar Gateway space station and later the Deep Space Transport/Mars Missions, will for the first-time place humans outside of Earth’s protective magnetic field exposed to significant quantities of unmitigated space radiation and weightlessness for many months to years at a time.

The health effects of low Earth orbit (LEO) spaceflight (e.g. the International Space Station) are multiple, with much of the research communities’ intense focus centred on the musculoskeletal, neurological, ocular and cardiovascular degeneration that can manifest as early as a few weeks into a mission. Notably, the effects of LEO spaceflight on many other organ systems are less clear, with indispensable organs such as the kidneys receiving relatively little attention in the absence of overt symptoms. However, in the face of extended periods of deep space travel health issues may only present with late onset due to cloaked subclinical pathophysiology and chronic damage eating into the extensive functional reserves of such organs.

The kidneys are critical in regulating blood pressure, by controlling both the renin/angiotensin/aldosterone system (RAAS) and the reabsorption of sodium chloride and water from the urine. In microgravity, the cephalad redistribution of blood volume with the concomitant decrease in the pressure gradient should reduce kidney perfusion. In turn, a lower perfusion pressure within the renal artery should lead to a greater release of renin and activation of the RAAS. However, studies show a marked reduction of diuresis and natriuresis in space with the RAAS, and antidiuretic hormone (ADH) increasing through unknown mechanisms^1–3^. These unexplained effects, be they mediated through known homeostatic mechanisms, or more theoretical ones, such as tensegrity^4^, are germane to kidney stone formation (i.e. nephrolithiasis). Astronauts have an unusually high rate of kidney stone formation, with 1-year post-flight astronauts experiencing incidence rates of 2–7 times that of pre-flight estimates, and in-flight risk estimated to be double that again^5^. This is of mission critical significance, one Soviet in-flight renal stone episode nearly caused a mission termination due to the severe symptoms, but was relieved by spontaneous stone passage by the cosmonaut just before an urgent deorbit was initiated^6^. It has been demonstrated that spaceflight associated changes in urinary biochemistry favour kidney stone formation^7–9^. Microgravity may have a direct effect on the crystallisation and nucleation of nascent kidney stones^10^, but this is dwarfed by the net biochemical urinary changes historically attributed to the microgravity-induced bone demineralisation observed in spaceflight. A school of thought that has led to a paucity of research into other pathophysiological phenomena that may be contributing to the unusually high incidence of nephrolithiasis.

The kidney is a plastic organ, and is capable of dynamically remodelling the architecture of the nephron in response to changes in blood pressure or dietary potassium. An 18 day potassium-free diet resulted in a 31% increase in rat kidney weight, which was mainly seen in the medullary collecting duct^11^. Changes in the size of nephron segments in wild type mice have been observed using advanced imaging techniques in a period as short as three days^12^. So, it is plausible that spaceflight-induced shifts in fluid and electrolyte handling could precipitate maladaptive remodelling events that contribute to the underlying pathophysiology.

In addition to the well-studied environmental stressor of microgravity, deep space missions beyond the protection of the geomagnetosphere (the Van Allen belts) are exposed to space radiation. Space radiation comprises both intermittent events (e.g. Solar Particle Events or Solar Proton Events) with the most energetic being a Coronal Mass Ejection and Solar Wind and Galactic Cosmic Radiation (GCR), with GCR having the most significant health impacts.

There is concern regarding the carcinogenic effect of GCR exposure in the planned Mars Missions. However, the kidney is an exquisitely radiation sensitive organ; it is the dose limiting organ in abdominal radiotherapy^13^. Chronic kidney dysfunction can occur with acute low linear energy transfer (LET) γ-radiation doses as low as <0.5 Gy^14^, the LET dose expected on a Mars Mission. However, GCR comprises low-LET x- and γ-rays with protons and high energy ‘HZE’ ions of heavier elements (e.g. silicon, titanium, iron). It has been established that exposure to HZE ions results in mitochondrial damage^15,16^. The renal proximal tubule is heavily dependent on mitochondrial respiration^17^ and is one of the main tubular segments in which damage is associated with kidney failure^18^. Indeed, proximal tubular injury is a prominent histological feature in medical (low-LET) radiation nephropathy^19^. Due to their dependence on mitochondrial aerobic respiration, proximal tubular cells are extremely vulnerable to mitochondrial damage that we would expect from HZE exposure in addition to low-LET radiation damage.

We hypothesised that the biochemical urinary changes seen in spaceflight might reflect functional and morphological changes detectable in the kidney secondary to microgravity, possibly due to abnormal renal perfusion.

Further, we hypothesised that GCR exposure will cause mitochondrial and therefore proximal tubular dysfunction, leading to tissue damage and potentially irreversible loss of renal function, in addition to the known micro and macrovascular damage caused by GCR.

**Fig. 1:**
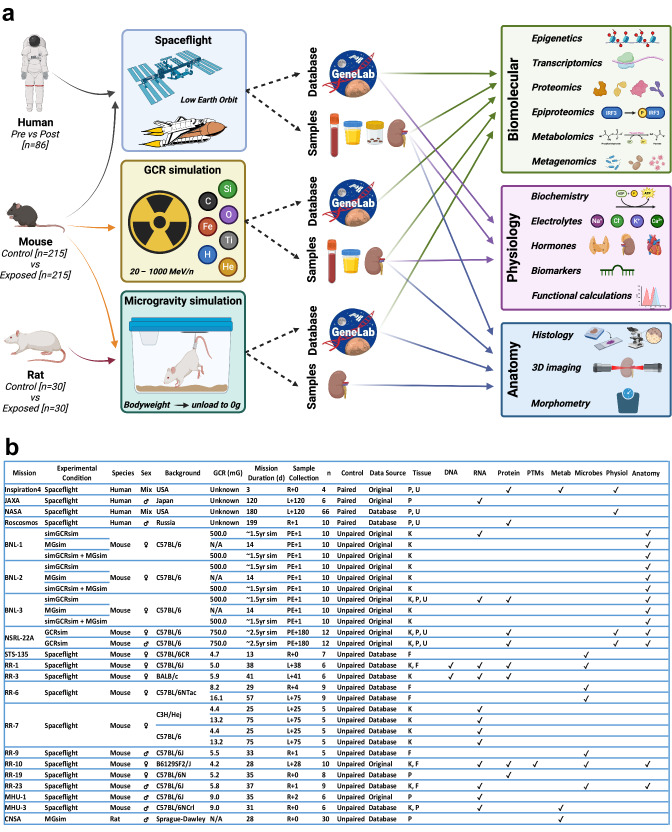
Study design. **a** Infographic representation of the study design. (Created with BioRender.com released under a Creative Commons Attribution-NonCommercial-NoDerivs 4.0 International license). **b** Details of spaceflight missions and simulations included in the project. R return, L launch, PE post-exposure, P plasma, U urine, K kidney; F faecal. **a**, **b** N numbers correspond to the number of experimental individuals that were exposed to spaceflight or a simulated spaceflight condition, and to control condition individuals were omitted from the presented counts to ensure paired and unpaired experiments were comparable.

In order to explore these questions, we took a pan-omics approach incorporating anatomical and physiological parameters from 20 independent human and rodent spaceflight missions/simulations that had extant data and/or samples from which we could generate novel data (Fig. 1).

## Results

### Primary changes in kidney function increase the risk of spaceflight-induced nephrolithiasis

**Fig. 2:**
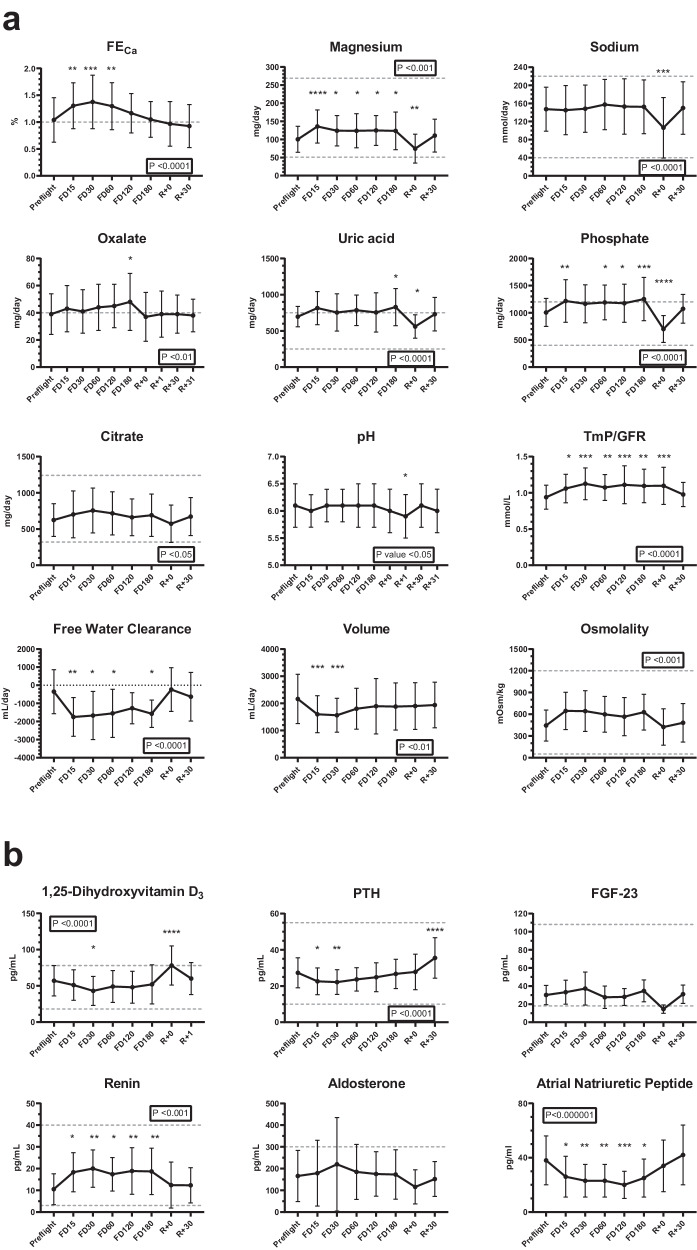
Human spaceflight urine and plasma physiological measurements. **a** Urinary chemistries and **b** blood plasma endocrine profiles from NASA astronauts (*n* = 66) exposed to spaceflight up to 180 days measured pre-flight, during (FD flight day) and after returning (R). Dashed lines represent upper and lower normal clinical values, or upper limit where only a single line is present. Data are presented as mean ± SD. Boxed *P*-values report the repeated measure one-way ANOVA result; all timepoints were compared to pre-flight by pairwise multiple comparison Bonferroni corrected *post-hoc* tests (*p < 0.05, **p < 0.01, ***p < 0.001, ****p < 0.0001) (For exact *P*-values see Supplementary Table 1). FE_Ca_ Fraction Excretion of Calcium. TmP/GFR ratio of tubular maximum reabsorption rate of phosphate to glomerular filtration rate. 1,25-dihydroxyvitamin D3 calcitriol. PTH parathyroid hormone. FGF-23 fibroblast growth factor-23.

We analysed urinary and plasma parameters of interest for nephrolithiasis in 66 astronauts that stayed on the International Space Station (ISS) for up to 180 days. Values were analysed across time, including before, during and after flight. Our data showed an increased urinary excretion of metabolites and electrolytes that are considered risk factors for nephrolithiasis (Fig. 2). In the figure, urinary excretion of calcium is expressed as fractional excretion (FE_Ca_). In the absence of plasma calcium abnormalities FE_Ca_ should be < 1%; in astronauts, FE_Ca_ increases significantly during flight. This urinary abnormality normalises on return to Earth (Fig. 2). A less significant increase was seen in oxalate, phosphate, uric acid urinary excretion during spaceflight, all rapidly decreasing at return with some parameters transiently overcorrecting. Urinary citrate did not significantly change and remained within the normal range. There was also a decrease in urinary volume during spaceflight and an increase in urinary osmolality (Fig. 2). Urinary electrolytes (chloride, sodium and potassium) remained stable during spaceflight compared to pre-flight with the exception of magnesium that, although within the normal range, significantly increased (Fig. 2**;** Supplementary Fig. 1A). Calculated values including estimated glomerular filtration rate (eGFR), aldosterone:renin ratio, FE for urinary electrolytes and water, transtubular potassium gradient (TTKG) and the ratio of tubular maximum reabsorption of phosphate (TmP) to GFR can be found in Fig. 2 and Supplementary Fig. 1A, B. In addition, Supplementary Fig. 1C shows plasma values from the SpaceX Inspiration4 mission. Although these astronauts only spent 3 days in space, there was a degree of eGFR instability detected several months after returning to Earth.

Although, most of the metabolite changes support the emergence of pro-lithogenic profile during spaceflight, they do not entirely explain the increased risk of nephrolithiasis in astronauts, suggesting that there might be other previously unidentified mechanisms underpinning this phenomenon.

**Fig. 3:**
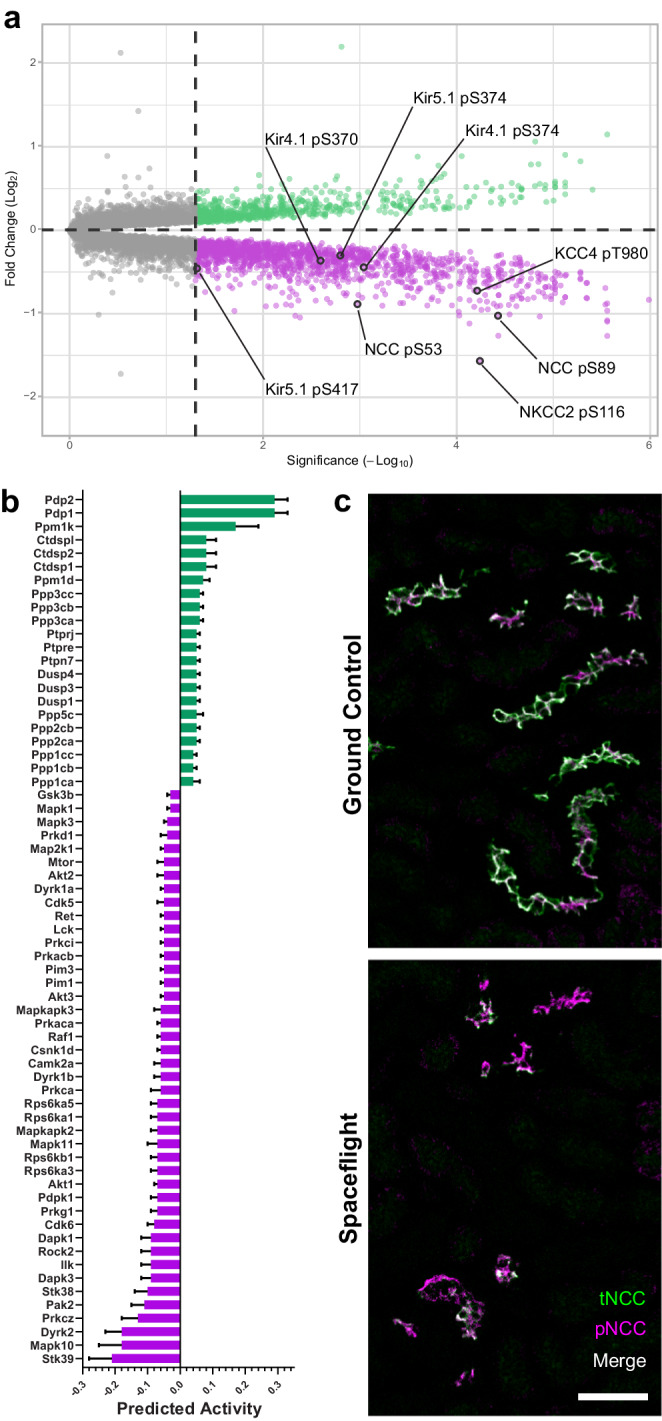
Spaceflight-induced changes in the kidney phosphoproteome. **a** Volcano plot of differentially phosphorylated amino acid residues detected in phosphopeptide-enriched kidney protein extract from RR-10 spaceflight-exposed mice (28 days). Regulatory phosphosites in transporters and channels related to the thick ascending limb of the loop of Henle and distal convoluted tubule are highlighted. Green indicates a statistically significant increase in peptide phosphorylation, magenta indicates a decrease, and grey represents no significant change. An unadjusted two-tailed -Log_10_(Adjusted *P*-value) of 1.3 was considered statistically significant (indicated by the dashed line intersecting the x-axis). Kir4.1 (*KCNJ10*); Kir5.1 (*KCNJ16*); NKCC2 (*SLC12A1)*; NCC (*SLC12A3*); KCC4 (*SLC12A7*); pT, phospho-threonine; pS, phospho-serine. **b** Kinase-Substrate Enrichment Analysis (KSEA) was performed using the Robust Inference of Kinase Activity (RoKAI) App v2.2.1 to predict kinase and phosphatase activity levels using phosphoproteomics from RR-10 spaceflight-exposed mice (28 days) kidney tissue. Bars in blue represent downregulated activity and those in red represent upregulated activity. An adjusted *P*-value of < 0.05 was considered significant. Data are mean SEM. An FDR of 5% was used as a cutoff. *N* = 10 biologically independent animals per group (flight vs ground control). 4257 single-site phosphopeptides (Ser/Thr/Tyr) were used as initial input, 495 of which were matched to known kinase/phosphatase targets, that were then used to calculate respective predicted kinase/phosphatase activities. Input and results files for this can be found at OSD 466.**c** Representative confocal images of RR-10 spaceflight-exposed mice (28 days) kidney sections [*n* = 7 spaceflight / *n* = 10 ground control) stained with an anti-NCC pT44, pT48 & pT53 antibody (green; pNCC) and a total NCC antibody (magenta; tNCC) to visualise NCC phosphorylation-dependent activation (green and magenta overlap will appear as white); 50 µm scale bar.

To investigate this, we initially explored RR-10 spaceflight mouse kidney tissue epiproteomic data to infer functional changes. Post-translational modifications of channels, transporters and pumps can alter their activity, and chief among these is phosphorylation status for which we noted several thousand significantly altered phosphosites (Fig. 3a**;** Supplementary Data 1), suggesting a general increase in phosphatase and decrease in kinase activities (Fig. 3b). In particular, two of the most dramatically dephosphorylated proteins were the thiazide-sensitive Na-Cl cotransporter (NCC), encoded by *SLC12A3*, expressed in the distal convoluted tubule (and validated by phospho-specific antibody staining in Fig. 3c) and furosemide-sensitive Na-K-Cl cotransporter (NKCC2), encoded by *SLC12A1*, expressed in the thick ascending limb of the loop of Henle (Fig. 3a). These transporters requires phosphorylation of key residues at their N-terminus for activation^20^, and NKCC2 is regulated by the calcium-sensing receptor (CaSR) and genetic (i.e., Bartter syndrome) or pharmacological (e.g., furosemide) impairment of NKCC2 leads to hypercalciuria, and is associated with nephrolithiasis. Whereas changes in NCC activity can lead to alterations in calcium handling and bone mineral density.

**Fig. 4:**
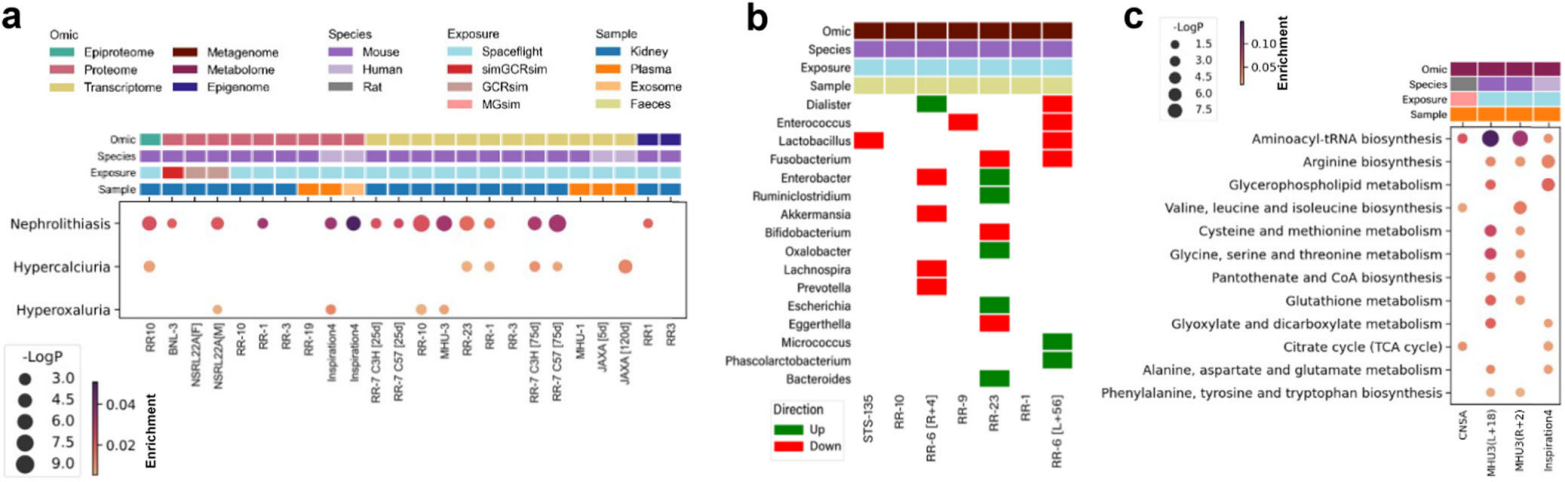
Multi-mission pan-omic investigation of the contributors to nephrolithiasis risk during spaceflight. **a** Multi-omic over-representation analysis of DisGeNET gene-disease associations related to nephrolithiasis. To integrate datasets from different omics modalities, species, missions and tissues, all biomolecules (e.g. phosphopeptides, proteins, transcripts and methylated DNA) were converted to the human orthologs where necessary and linked back to their HGNC gene symbol, aggregated and collapsed to single genes (e.g. multiple phosphosites, isoforms, CpG sites). An unadjusted, two-tailed -Log_10_(*P*-value) of 2 was considered significant for ontological term enrichment. **b** Categorical heatmap of differential abundance directionality in nephrolithiasis-related faecal microbial taxa after spaceflight. An unadjusted, two-tailed -Log_10_(*P*-value) of 1.3 was considered significant. **c** Over-representation analysis of KEGG module metabolic pathways with replication in at least two datasets. An unadjusted, two-tailed -Log_10_(*P*-value) of 1.3 was considered significant for ontological term enrichment. **a**, **c** Enrichment ratio; the number of differentially regulated hits in a dataset that belong to a given ontological term, normalised to the total number of statistically significant hits in the respective dataset.

**Fig. 5:**
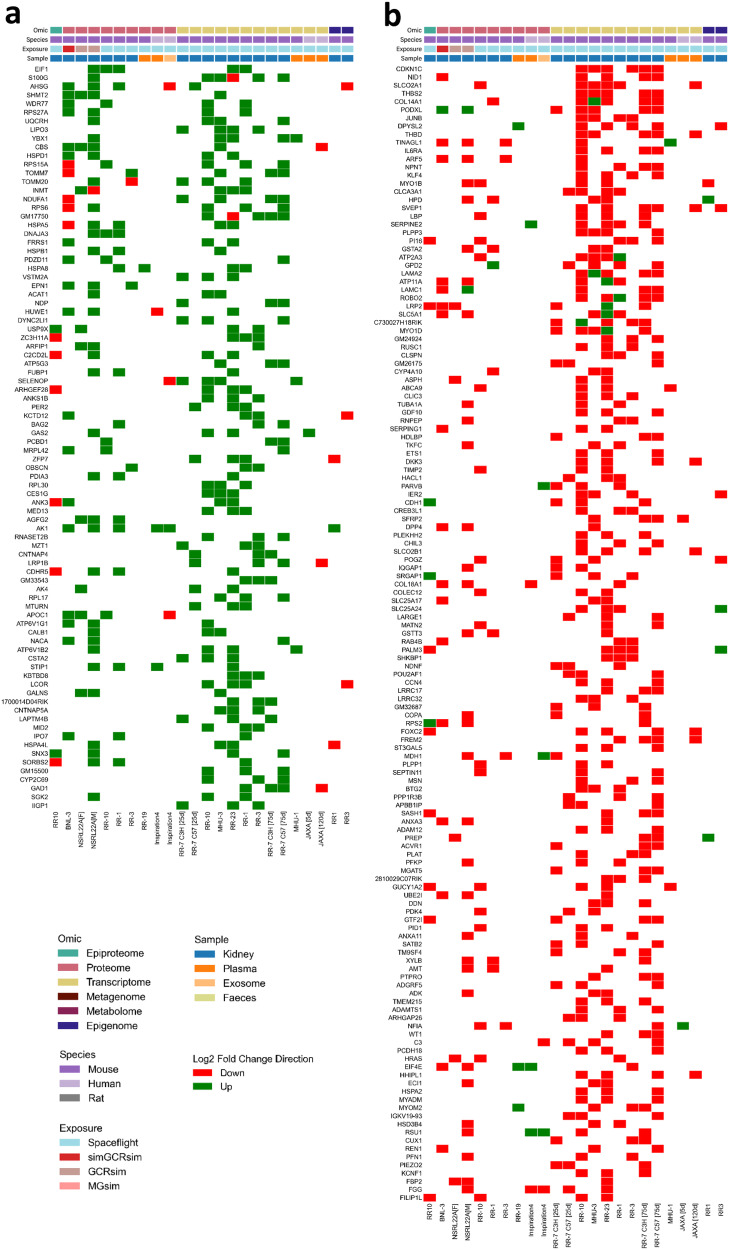
Multi-mission multi-omic consensus on differentially regulated gene products in the kidney. Categorical heatmap of **a**, upregulated and **b**, downregulated gene products in exposure groups (e.g. spaceflight, GCRsim) compared to control groups (e.g. ground control, sham). To integrate datasets from different omics modalities, species, missions and tissues, all biomolecules (e.g. phosphopeptides, proteins, transcripts and methylated DNA) were converted to the human orthologs where necessary and linked back to their HGNC gene symbol, aggregated and collapsed to single genes (e.g. multiple phosphosites, isoforms, CpG sites). These differentially regulated gene products (DRGP) were ranked and represented in descending order using the following rules: (1) only DRGPs with a *P*-value < 0.05 by Wald test were counted as significant and plotted; (2) Each DRGP was assigned a score of +1 each time it was upregulated or −1 each time it was downregulated; (3) only DRGPs observed in proteome and transcriptome kidney-specific datasets were used for the calculation of the ranking score to avoid confounders, although additional datasets (epiproteome, epigenome and those from plasma/exosomes sources) were plotted for visualisation comparison purposes; (4) The resulting sum of scores for each DRGP was then calculated and multiplied by the number of times the DRGP was observed in the kidney-specific proteome and transcriptome datasets; (5) To increase confidence only DRGPs with a product score of absolute value 9 or higher (but excluding a score of 12) were plotted as these will have a directionality consensus of at least 3 datasets above the number of disagreeing datasets.

To look for corroborating evidence of primary changes in renal function, we analysed plasma and kidney tissue multi-omic data from human and mouse samples (Fig. 4a**;** Supplementary Fig. 2**;** Supplementary Data 2) for disease ontologies related to kidney stone formation. The nephrolithiasis and related ontologies were heavily represented across different datasets, providing orthogonal confirmation of real changes in the gene products comprising this ontology for spaceflight conditions vs controls. For example, α-tubulin (*TUB1A1*) is known to protect against cell-crystal adhesion^21^ but is decreased across multiple kidney datasets in our study (Fig. 5**;** Supplementary Data 3). In contrast, hypercalciuria and hyperoxaluria ontologies were scantily represented, implying that standard terrestrial causes of dysregulated urinary calcium and oxalate were not particularly enriched and therefore not strongly implicated in kidney stone formation.

The directional changes in nephrolithiasis-related faecal microbiome abundances between spaceflight and ground controls are illustrated in **(**Fig. 4b**;** Supplementary Data 4). Overall, these microbiota changes paint a mixed picture with some changes expected to confer a protective profile against nephrolithiasis and others tending to contribute to increased risk, implying that the increased urinary oxalate excretion is unlikely to be primarily diet or microbiota-dependent. Nevertheless, whole faecal microbiome KEGG analysis shows some enrichment of glyoxalate (a precursor to oxalate) and arginine metabolism (Supplementary Fig. 3).

Pathway analysis of novel plasma metabolomic data from humans and rodents under spaceflight conditions (Fig. 4c**;** Supplementary Data 5) shows a striking over-representation of Aminoacyl-tRNA biosynthesis and arginine biosynthesis metabolic pathways.

Aminoacyl-tRNA biosynthesis is the ubiquitous housekeeping process of charging amino acids to their cognate tRNAs and providing the substrates for global protein synthesis. In addition to this canonical function, it has become clear that aminoacyl-tRNA synthetases are involved in a variety of physiological and pathophysiological processes including angiogenesis, inflammation, translational control and post translational modification^22–24^.

Arginine metabolism has been implicated in toxic nephrolithiasis^25^ and glycerophospholipids are the main lipid component of cell membranes and pathway enrichment is compatible with remodelling events (reviewed by Han^26^). Citric Acid Cycle pathway is common to many metabolomic analyses of oxalosis.

### Structural remodelling of the nephron occurs within a month of spaceflight exposure

**Fig. 6:**
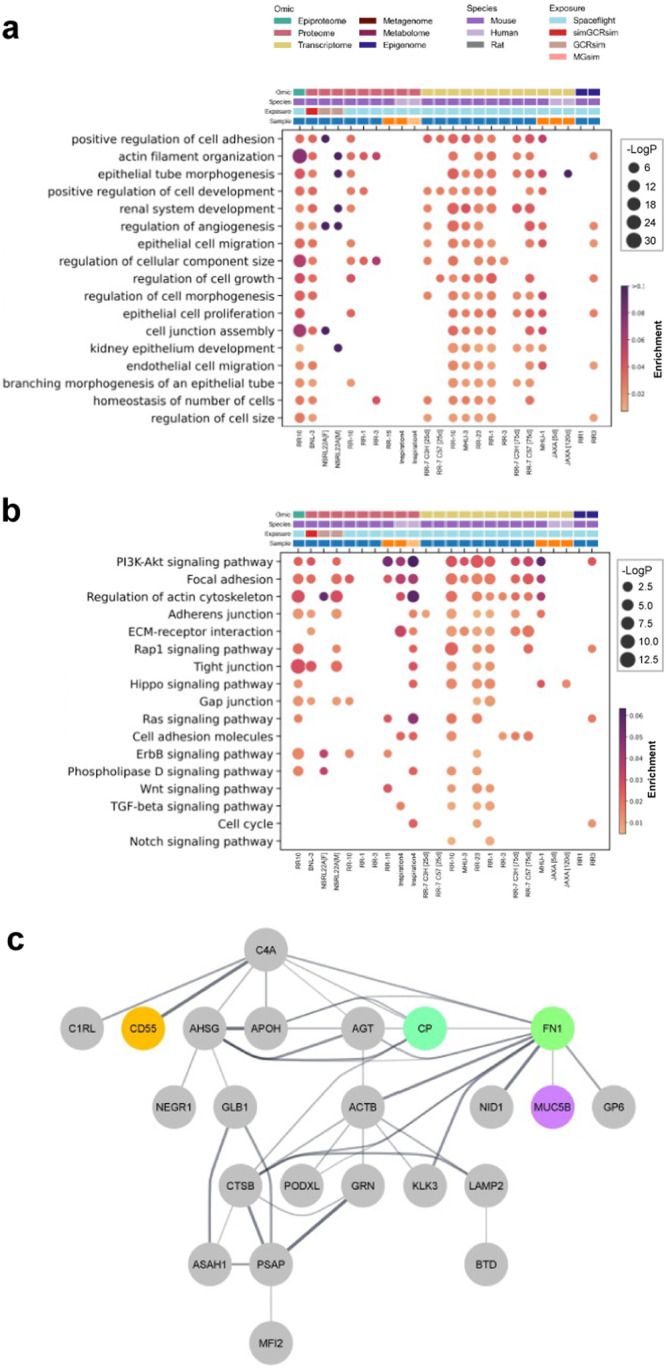
Biomolecular evidence for spaceflight-induced nephron remodelling. **a** A curated list of multi-omic over-representation analysis of GO—biological process ontological terms related to remodelling. **b** A curated list of multi-omic over-representation analysis of KEGG pathway ontological terms related to remodelling. **a**, **b** To integrate datasets from different omics modalities, species, missions and tissues, all biomolecules (e.g. phosphopeptides, proteins, transcripts and methylated DNA) were converted to the human orthologs where necessary and linked back to their HGNC gene symbol, aggregated and collapsed to single genes (e.g. multiple phosphosites, isoforms, CpG sites). An unadjusted, two-tailed -Log_10_(*P*-value) of 2 was considered significant for ontological term enrichment. Enrichment ratio; the number of differentially regulated hits in a dataset that belong to a given ontological term, normalised to the total number of statistically significant hits in the respective dataset. **c** STRING protein-protein interaction network of previously absent inflammation-associated and collagen I-associated ECM proteins that appeared in cosmonauts’ urine (*n* = 10) after 166–199 days of spaceflight exposure.

To investigate whether renal remodelling was occurring in spaceflight, we performed Gene Ontology (GO) and Kyoto Encyclopaedia of Genes and Genomes (KEGG) pathway enrichment analysis across 24 datasets obtained from 2 human and 13 rodent spaceflight missions and 2 rodent GCR simulation experiments. The datasets were epigenomic, transcriptomic, proteomic and phosphoproteomic. GO pathways (Fig. 6a**;** Supplementary Data 6) that are repeatedly significantly enriched across the datasets are compatible with remodelling events occurring in the kidney, these include regulation of cell adhesion, actin filament organisation, epithelial tube morphogenesis and renal system development pathways.

Enriched KEGG pathways (Fig. 6b**;** Supplementary Data 7 likewise include focal adhesion, tight junction, gap junction, sphingolipids, actin and cell cycle pathways, again consistent with remodelling.

Proteomic analysis of urine from the Russian Roscosmos human spaceflight mission (Fig. 6c) shows extracellular matrix (ECM) and lipid-turnover related proteins that were enriched 7 days after spaceflight compared to baseline. These include Fibronectin (*FN1*; involved in the organisation of the ECM), Cathepsin B (*CTSB*; maintains turnover of glomerular basement membrane^27^), Apolipoprotein H (*APOH*; which binds to cell surface phospholipids) and Proactivator Polypeptide (*PSAP*; involved in the lysosomal degradation of sphingolipids). Notable among these are Nidogen-1 (*NID1*; a key part of the nephron basement membrane) and Podocalyxin (*PODXL*; a major constituent of the glycocalyx of podocytes in the glomerulus) which are two of the most frequently downregulated gene products across kidney datasets in our study (Fig. 5**;** Supplementary Data 3).

**Fig. 7:**
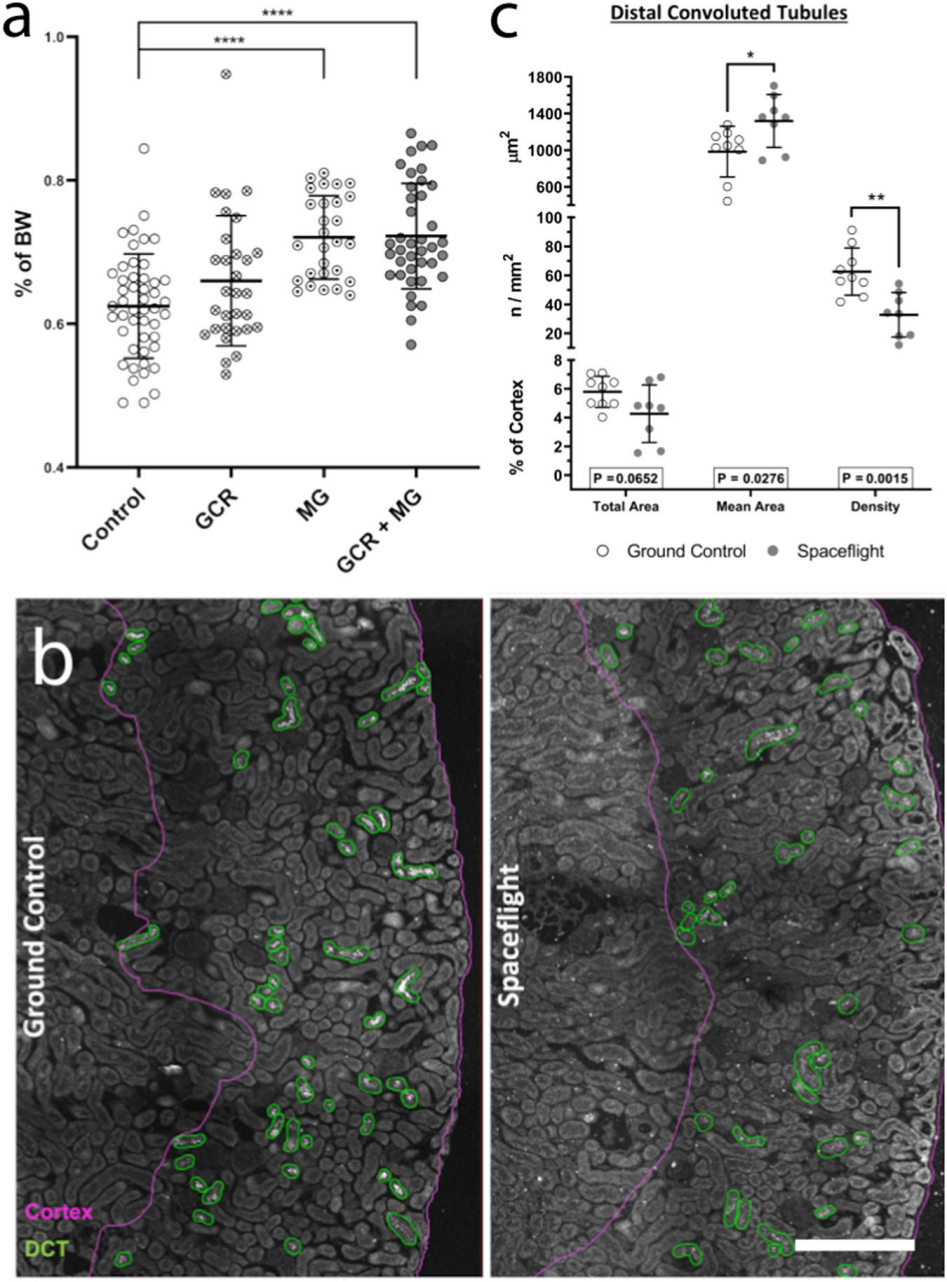
Anatomical evidence for spaceflight-induced nephron remodelling. **a** Morphometry of kidney mass was assessed by wet weights in 1 G, the weight of both left and right kidneys were averaged, and the kidney weights were normalised against bodyweight (BW) for the same animal and expressed as a percentage. All ground control and sham animals that received no exposure treatment were grouped into control. Animals that only received either full or simplified galactic cosmic radiation (GCR) simulations were grouped into GCR. Animals that only underwent hindlimb unloading microgravity simulation were grouped into microgravity (MG). Animals that underwent a combination of GCR and MG, or were exposed to spaceflight, were grouped into GCR + MG. Data are presented as mean ± SD. One-way ANOVA with pairwise Dunnett’s multiple comparison post-hoc tests (*p < 0.05, **p < 0.01, ***p < 0.001, ****p < 0.0001). *N* numbers are control = 48, GCR = 30, MG = 28, MG + GCR = 39 biologically independent animals per group. **b** Representative confocal images showing the annotations of cortex regions (magenta borders) and distal convoluted tubules (DCT, green) in RR-10 spaceflight-exposed mice (28 days) from whole slide images of kidney sections immunolabelled with tNCC/pNCC as DCT markers. 200 µm scale bar. **c** Morphometric analysis of DCTs assessed the average area per tubule (top Y-axis), the density of no. of tubules per unit area of cortex (middle Y-axis), and the total summed area of all DCTs as percentage of cortex area. Data are mean ± SD. *P*-value of  0.05 by students t-test was considered significant. *N* = 10 biologically independent animals per group. Source data are provided as a Source Data file.

To determine the extent of any potential structural remodelling, we looked for macroscopic changes in GCR and/or microgravity exposed rodent kidneys. Much like Elger and co-workers^11^, who found an increase in kidney weight of 30% in chronically potassium depleted animals, we found that microgravity (simulated) and microgravity+GCR (real and simulated) exposed animals had significantly greater kidney weights (relative to bodyweight) than control animals (Fig. 7a).

To establish that these changes weren’t merely due to alterations in extracellular fluid content, quantitative and qualitative histomorphometry was performed on mouse kidneys from the RR-10 spaceflight mission. Samples were stained for the canonical distal convoluted tubule marker Na-Cl cotransporter (NCC; encoded by *SLC12A3*), as this segment is known to be the most plastic in the nephron. Our data suggest that RR-10 mice have chronic dephosphorylation of NCC in addition to potentially altered basolateral potassium handling via Kir4.1/5.1 and KCC4, both of which are known to induce distal convoluted tubule remodelling^12,28^ (Fig. 3a, c**;** Supplementary Data 1).

Qualitative assessment of 3D images of optically cleared tissues revealed no obvious lesions or anatomical abnormalities (See videos in Supplementary Movie 1 & 2). However, quantitative analysis of distal convoluted tubules revealed that spaceflight led to a reduction in tubule density, but that these retained tubules had a larger average size, demonstrating tubular segment remodelling (Fig. 7b, c).

### Multi-omic analyses reveal convergent disease pathways induced by spaceflight

**Fig. 8:**
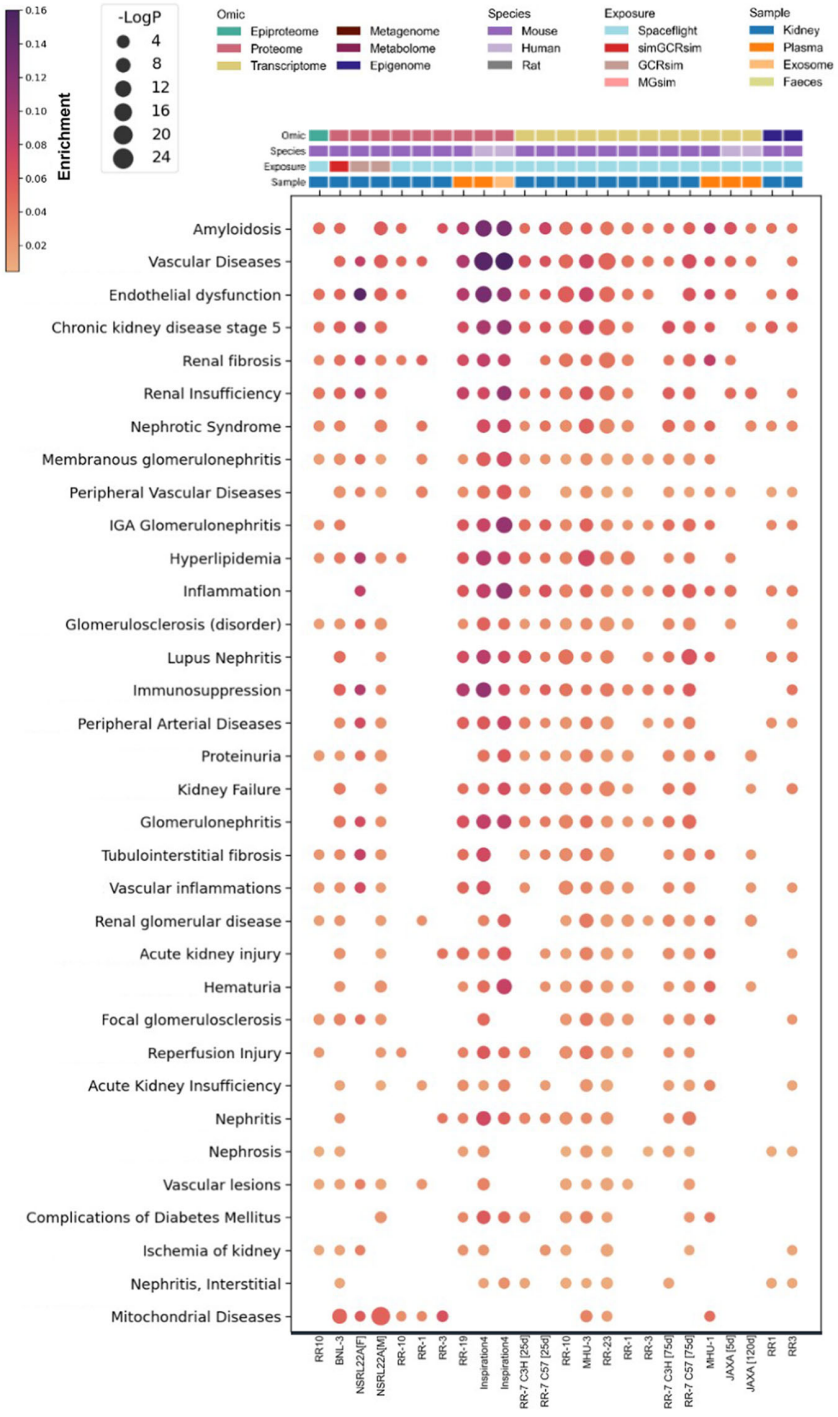
Biomolecular evidence for spaceflight-induced kidney damage. A curated list of enriched gene-disease associations are presented for DisGeNET ontological terms relevant to kidney health. These were ranked and represented in descending order using the following rules: 1) No. of mission datasets it replicated in; 2) most significant *P*-value; 3) greatest enrichment. To integrate datasets from different omics modalities, species, missions and tissues, all biomolecules (e.g. phosphopeptides, proteins, transcripts and methylated DNA) were converted to the human orthologs where necessary and linked back to their HGNC gene symbol, aggregated and collapsed to single genes (e.g. multiple phosphosites, isoforms, CpG sites). An unadjusted, two-tailed Log_10_(*P*-value) of 2 was considered significant for ontological term enrichment. Enrichment ratio; the number of differentially regulated hits in a dataset that belong to a given ontological term, normalised to the total number of statistically significant hits in the respective dataset.

To understand the common processes occurring across the multiple human and rodent, spaceflight and GCR exposed missions, omics data were analysed against DisGeNET, KEGG and GO databases. Significantly enriched kidney-relevant disease ontologies that were reproduced in our diverse cohort of datasets were plotted in Fig. 8 for DisGeNET (Supplementary Fig. 2**;** Supplementary Data 2).

Many of the same ontologies are represented in both the spaceflight and simulated GCR experiments. Ontologies that are particularly enriched include amyloidosis, nephrotic syndrome, and membranous glomerulonephritis, reflecting the presence of acute phase and inflammatory gene products in addition to the profibrotic and cell death gene products represented in the chronic kidney disease (CKD) stage 5, renal fibrosis and renal insufficiency ontologies. The heavily enriched peripheral vascular disease and endothelial damage ontologies reflect the known endothelial and microvascular damage marker gene products enriched across the multiple GCR and spaceflight datasets.

Mitochondrial diseases are enriched in the proteomics datasets of kidney tissues (Fig. 8) and is further supported by the enrichment of ontologies relating to mitochondrial suborganellular structures, oxidative phosphorylation, oxidative stress, reactive oxygen species, apoptosis, cellular senescence, HIF-1 and TNFα signalling in GO (Supplementary Fig. 4**;** Supplementary Data 6) and KEGG (Supplementary Fig. 5**;** Supplementary Data 7) over-representation analyses. Mitochondria may also be showing signs of stress-induced fusion (Supplementary Fig. 6), although future studies would be needed to specifically address changes in mitochondria morphology.

As a final step we used the Scalable Precision Medicine Open Knowledge Engine (SPOKE)^29^ knowledge graph tool which is capable of integrating heterogeneous datasets from various biological levels, assay modalities, tissue types, organisms and experimental conditions to validate our findings (Supplementary Fig. 7).

### A GCR exposure with a dose-equivalent to a Mars roundtrip induces renal damage and dysfunction

LEO spaceflight mission participants are exposed to comparatively low doses of GCR due to the protection provided by Earth’s magnetic field. Therefore, to disentangle the impacts of GCR from microgravity and evaluate the consequence of unmitigated GCR exposure, we examined tissues that had only been exposed to simulated GCR. To do this we utilised NASA’s ground-based GCR simulator at Brookhaven National Laboratory, where animals either had an acute exposure to a ~ 1.5-year dose-equivalent (0.5 Gy) in the simplified 6-beam/5-ion GCR simulator followed by rapid 24 h sacrifice (BNL experiments) or were given an acute exposure to a ~ 2.5-year dose-equivalent (0.75 Gy) in the full 33-beam/7-ion GCR simulator with sacrifice at 6 months post-exposure (NSRL-22A experiment).

Pathogenic serum miRNA species have been previously implicated to mediate tissue damage in spaceflight^30^, and GCR is a plausible pathogenic factor. Weighted correlation network analysis (WGCNA) of plasma miRNAs revealed three clusters that generally trended downward in BNL-1 (Supplementary Fig. 8). However, investigation of miRNA predicted mRNA targets and DisGeNET analysis of these targets suggested that these circulating miRNAs would be minor players in the context of renal damage in the acute setting (Supplementary Data 8).

**Fig. 9:**
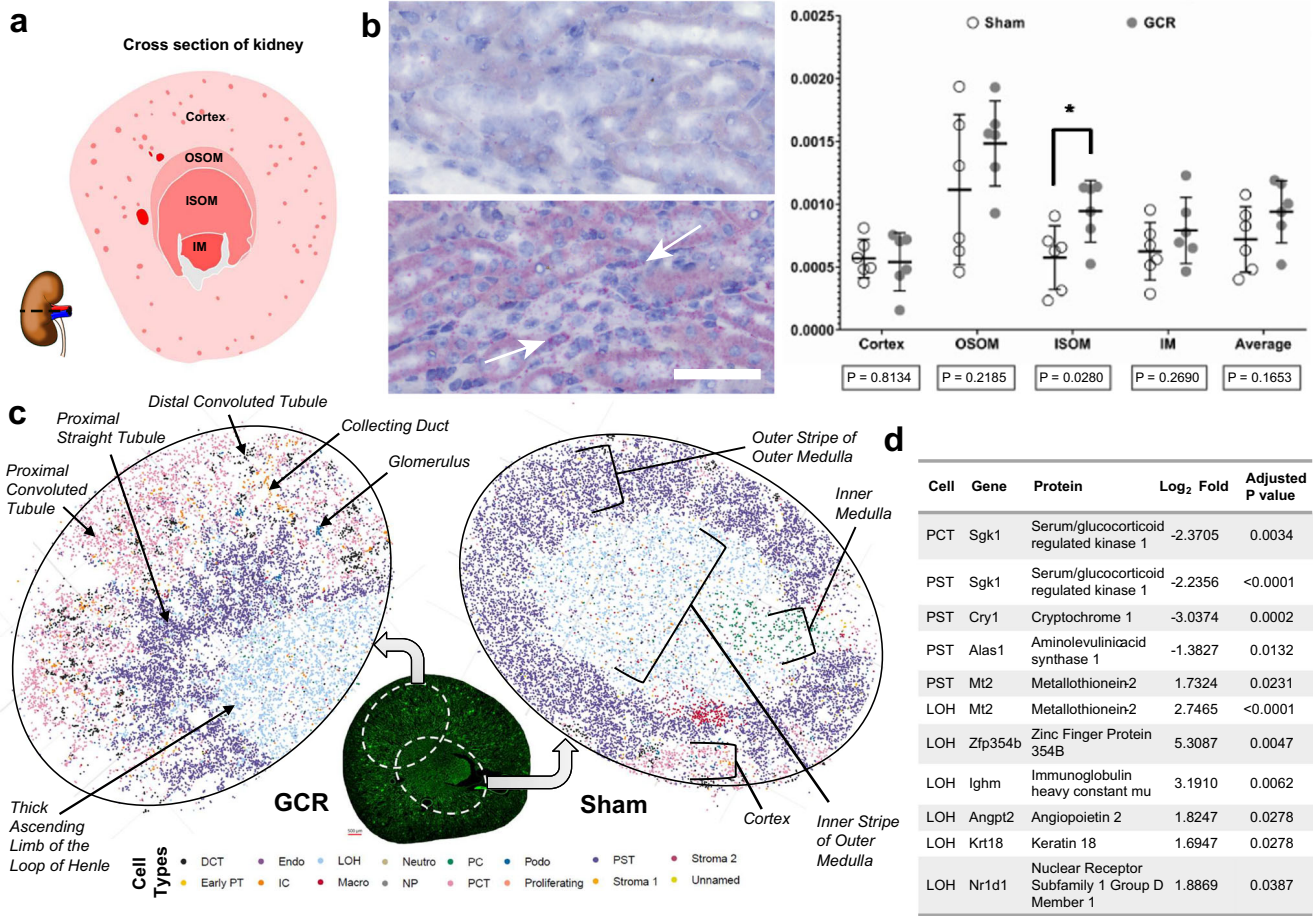
Acute impact of galactic cosmic radiation (GCR) on kidney health. **a** Schematic representation of an axial cross section of mouse kidney hemisected at through the hilum as shown. Annotations of gross anatomical regions of the kidney used for spatial RNA expression analyses. **b** Representative images of miR-125b staining (small red dots) in the outer medulla of haematoxylin-stained kidney sections from BNL-3 simGCRsim-exposed mice (~ 1.5-year-dose equivalent) harvested ~ 24 h post-exposure. White arrows indicate concentrations of miR-125b staining around capillaries in the interstitium; 50 µm scale bar. Data are mean ± SD. A *P*-value of < 0.05 by students *t*-test was considered significant. Average; the simple arithmetic mean of the four anatomical regions, *n* = 6 biologically independent animals per group **c** Gross anatomical and microanatomy features annotated on exemplar slide-seq pucks of BNL-1 simGCRsim-exposed mice (~ 1.5-year-dose equivalent) harvested ~ 24 h post-exposure. Samples for pucks were taken to maximise the anatomical coverage from the approximate regions shown in the green (autofluorescence) axial section of mouse kidney. Beads are assigned identities according to their highest probable cell type classification. For scale, each dot represents a 10 µm bead. **d** The table shows the differentially regulated mRNA transcripts from **c,** captured by different cell typed beads after exposure to GCR. An adjusted *P*-value of < 0.05 by students *t*-test was considered significant. Abbreviations: OSOM outer stripe of outer medulla. ISOM inner stripe of outer medulla. IM Inner medulla. DCT distal convoluted tubule. Early PT or PTS1 early proximal tubule S1. Endo; endothelial cell. IC; intercalated cell. CTAL/MTAL or LOH; (cortical or medullary) thick ascending limb of the loop of Henle. Macro; macrophage. Neutro neutrophil. NP nephron progenitor cell. PC principal cell. PCT or PTS1/2 proximal convoluted tubule S1 + S2; Podo podocyte. PST or PTS3 proximal straight tubule S3. G glomerulus. DTL descending thin limb of the loop of Henle. ATL ascending thin limb of the loop of Henle. MD macula densa. CNT connecting tubule. CCD cortical collecting duct. OMCD outer medullary collecting duct. IMCD inner medullary collecting duct. Endo endothelial cell. IC intercalated cell. Macro macrophage. Neutro neutrophil. NP nephron progenitor cel.; Podo podocyte. The schematic map of a whole kidney’s cross section and the visualisation by actin filament fluorescent were published in The FEBS Journal 287 (2020) 1176–1194 [doi:10.1111/febs.15088] © 2019 Kumaran et al. and is licensed under CC BY 4.0. Source data are provided as a Source Data file.

Retaining biomolecular information within the gross and micro-anatomical context is important, especially when highly heterogenous tissues like the kidney (Fig. 9a). So, we next performed in situ hybridisation (RNAScope) and quantitative whole slide imaging to ascertain the presence and density of these pathogenic miRNAs in kidney tissues of BNL-3 mice. miR-125b was significantly increased in the inner stripe of the outer medulla (ISOM) (Fig. 9b), particularly in the vascular compartment (arrows) which corresponds to the thick ascending limb of the loop of Henle, the thin descending limb of the loop and the medullary collecting duct. miR-16 has a similar pattern of distribution and differential expression but fails to reach significance (Supplementary Fig. 9**)**. These miRNA species are implicated in vascular damage and the ISOM is also the site of the vascular bundle^31^, a complex structure which is the bottleneck of all blood flow to and from the inner medulla. Additionally, mRNA target prediction, KEGG and DisGeNET analysis of miR-125b and miR-16 suggest that these may mediate cell senescence, apoptosis, TGFβ signalling and oncogenesis (Supplementary Fig. 9).

To further understand the effect of simulated GCR on the segment-specific cell types of the kidney’s nephron (Fig. 9a), we performed 2D spatial transcriptomics (Slide-seq^32^) on BNL-1 GCR-exposed renal tissue. Cell types were inferred using a published renal cell atlas^33^, which were mapped onto individual data points which accurately reproduced anatomy (Fig. 9c). Individual significant transcriptional changes in specific cell types were then examined and top hits were analysed by KEGG and DisGeNET (Fig. 9d**;** Supplementary Data 10). Interestingly, immunoglobulin components were among the hits overexpressed by loop of Henle cells, presumably reflecting increased B-cell activity in the interstitium as supported by enrichment of the KEGG pathway for “leukocyte transendothelial migration” (Supplementary Data 10). SGK1 transcriptional activity was decreased in proximal convoluted and straight tubule cells (Fig. 9d). SGK1 is a master regulator of multiple epithelial membrane transporters, including canonical segment specific transporters. The data also support disruption of circadian rhythm in several tubule segments via Cytochrome-1 and Nuclear Receptor Subfamily 1 Group D Member 1 (also known as Rev-Erbɑ; encoded by *NR1D1*)^34^ (Fig. 9d), and hits on KEGG and DisGeNET (Supplementary Data 10). When it comes to damage markers, again the proximal straight tubule and thick ascending limb of the loop of Henle appear to be the most impacted, with decrease in *ALAS1*, and increases in *ANGPT2* and *KRT18* all prognostically poor for renal outcomes^35–37^ (Fig. 9d).

**Fig. 10:**
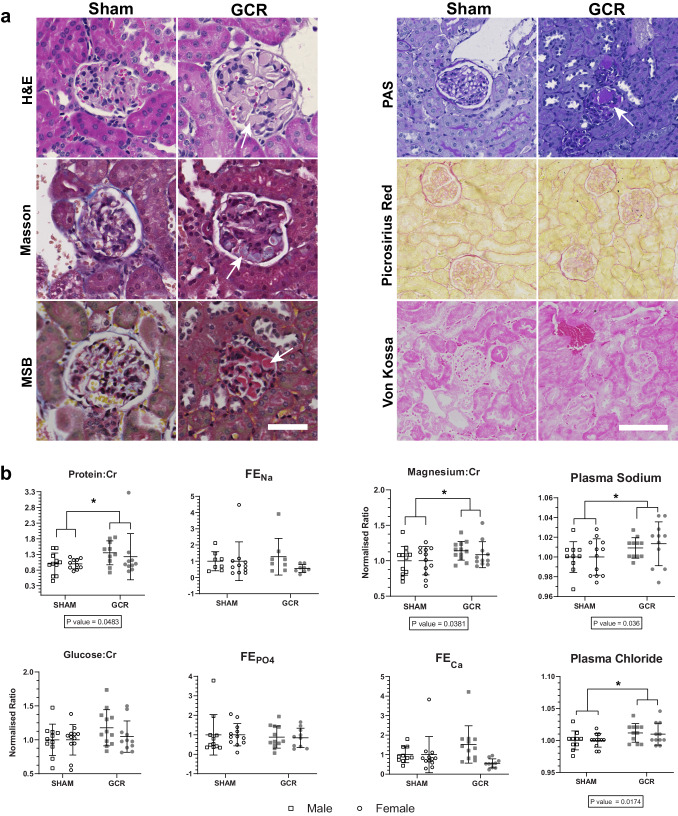
Chronic impact of galactic cosmic radiation (GCR) on kidney health. **a** Representative images of haematoxylin and eosin (H&E), Masson’s trichrome, Martius scarlet blue (MSB), Periodic acid–Schiff (PAS), Picrosirius red, and Von Kossa stained kidney sections from NSRL22A GCRsim-exposed mice (~ 2.5-year dose-equivalent) harvested ~ 6 months post-exposure. Arrows indicate microthrombi in the glomerular capillary tufts visible in H&E, Masson, MSB and PAS stains. Scale bar: 50 µm (left panel) and 100 µm (right). **b** Urine (Protein:Cr, Glucose:Cr, Magnesium:Cr, FE_Na_, FE_Ca_, FE_Mg_(Corr), FE_PO4_) and plasma (sodium, chloride) physiological measurements from NSRL22A GCRsim-exposed mice ( ~ 2.5-year dose-equivalent) harvested ~ 6 months post-exposure that were normalised to sham controls for illustrative purposes. Boxed P-values report the two-way ANOVA treatment group factor result. Data are mean ± SD. A *P*-value of < 0.05 was considered significant. Cr Creatinine, FE Fraction Excretion, Corr corrected for albumin, Ca calcium, Na sodium, Mg magnesium, P_O4_ phosphate, *N* = 12 biologically independent animals per sex per group. ‘Source data are provided as a Source Data file.

From a radiobiological perspective, the kidney is considered a late-responding tissue, and studies spanning at least 1-2 years may be required to appreciate the full impact of chronic GCR exposure on kidney health^38^. Although, some features of radiation-induced nephropathy and sub-clinical manifestations can emerge in < 6 months post-irradiation. To investigate this, serial tissue sections from NSRL-22A mice were histochemically stained for routine examination of potential kidney damage (haematoxylin and eosin, Masson’s trichrome stain, Martius Scarlet Blue) were examined by a blinded expert histopathologist (Fig. 10a).

No histological evidence of differences in interstitial fibrosis or tissue calcifications were observed between Sham and GCR exposed tissues stained with PAS, Picrosirius Red and Von Kossa (Fig. 10a). To confirm this, we quantitated the fractional interstitial area in the PAS stains which showed no difference between groups (mean 6.10% ± 2.10 vs 6.16% ± 2.27. *p* = 0.9623). As previously reported, first signs of fibrosis or calcifications may not present until after 6 months^38^, and therefore NSRL-22A may be too short a period to observe these disease features, despite significant enrichment of fibrosis-related DisGeNET ontologies in the kidney proteomics from the same animals (Supplementary Fig. 2; Supplementary Data 2). However, consistent with radiation-induced nephropathy, overt thrombotic microangiopathy was detected in GCR exposed tissues from 27% (3/11) female animals, and 0% (0/12) male animals. None was seen in tissue from sham exposed animals (Fig. 10a).

Given the biomolecular and histopathological signs of renal injury, we then looked for functional evidence of glomerular, proximal tubular and distal tubular dysfunction. Urinary and plasma biochemical analyses (Fig. 10b**;** Supplementary Fig. 10) showed significantly greater urinary protein excretion in GCR vs sham animals, consistent with a glomerular lesion or proximal tubule dysfunction, the latter supported by the reduction in the endocytic receptor megalin (*LRP2*) which normally reabsorbs proteins that escape into the ultrafiltrate (Fig. 5). There was a slight increase in urinary glucose, which is a highly specific, but not very sensitive marker of proximal tubular dysfunction in non-diabetic animals. While this did not reach significance (*p* = 0.1198), there was significant downregulation of sodium-glucose linked transporter, SGLT1 (*SLC5A1*) (Fig. 5), that would be consistent with proximal tubule dysfunction. Finally, there was significantly greater magnesiuria in GCR exposed animals, consistent with downregulated magnesium reabsorption in the loop of Henle or the distal convoluted tubule.

## Discussion

We have presented a large volume of data encompassing both human and rodent data, extant and novel multi-omics datasets, historical and extant clinical chemistry and novel imaging data from 16 spaceflight missions and 4 GCR exposures to examine the effect of spaceflight on kidney function.

Tubular remodelling, by which we mean structural and functional adaptation of the tubular nephron to environmental stimuli, is a well described yet underappreciated phenomenon. Tubular remodelling occurs not only in response to genetic modification of signal pathways^39^, but also chronic diuretic exposure^40^, kidney injury^41^, and both chronic^11^ and acute^42^ hypokalaemia. These changes occur rapidly, within 3 days of diet induced hypokalaemia^12^.

Our data robustly and orthogonally supports tubular remodelling occurring in microgravity with and without GCR. This is highly likely to have functional consequences, as tubular remodelling does in other scenarios^39^.

Renal remodelling in microgravity (possibly related to the cephalad fluid shift) may therefore be a primary event that causes subsequent dysregulation of serum and urine electrolyte homeostasis. This is supported by the prompt return to baseline of humans on return to terrestrial gravity.

Changes in urinary biochemistry are known to be critical in the pathogenesis of kidney stone formation; a condition that occurs disproportionally commonly in astronauts, and that is potentially mission critical.

The increased risk for nephrolithiasis in space has been historically attributed to the negative impact of microgravity on bone mineral density that leads to increased resorption of bone, release of calcium salts in the bloodstream, increased renal load and therefore increased urinary excretion of calcium and phosphate^6,8^. Our historical data (from 66 astronauts) confirms hypercalciuria, and the phosphoproteomic data shows decreased phosphorylation of NKCC2 (*SLC12A1*), which will cause decreased paracellular reabsorption of divalent cations in the loop of Henle, and thus renal calcium and magnesium wasting. Hypocitraturia was not observed; this is in line with previous observations by Pietryzk et al. of an approximate rate of 14% of hypocitraturia during spaceflight^6^, and Whitson et al. who found no significant hypocitraturia^8^.

Hyperoxaluria also occurs during spaceflight. Oxalate is freely filtered by the glomerulus and almost entirely excreted in the urine. Increased urinary oxalate reflects increased plasma oxalate load that could come from either increased endogenous liver production or increased absorption by the gut. The latter could be caused by several conditions such as increased dietary oxalate, malabsorptive conditions or altered microbiome. Dietary data were not available, and we assumed an absence of malabsorptive conditions. However, we do know that spaceflight affects the gut microbiome^43^. Metagenomic analysis of the microbiome showed that in the highest quality faecal microbiome dataset (namely RR-23) *Oxalobacter* sp., the bacterium primarily responsible for oxalate metabolism^44^, increases during spaceflight. Bacteroides, a bacterium that was found to be increased in stone formers^45^, and Bifidobacterium, a pro-biotic able to metabolise oxalate in the gut^46^, were enriched and depleted in spaceflight respectively. It is difficult, based on low replicability of data to ascribe a primary, or even secondary microbiome-related aetiology for increased stone formation in spaceflight.

Proposed interventions to prevent potential mission critical kidney stone formation in spaceflight include increasing urine output by maximising oral fluid intake^47^, oral potassium citrate^48^ (or potassium magnesium citrate^49^) supplementation and inflight management, including ureteral stenting^50^ and lithotripsy^51^. While these are sensible and may indeed be effective, none deal with the hypercalciuria that has been consistently shown in human space travellers. Oral or parenteral bisphosphonates have been used with some success^52^. Further, thiazide diuretics reduce urinary calcium excretion^53^ (possibly by ECV contraction causing increased proximal tubular calcium reabsorption^54^) and reduce age related bone mineral density loss^55^, and could represent a “two birds with one stone” therapeutic option.

Could the dephosphorylation of NKCC2 guide us toward a therapeutic target to reduce spaceflight induced hypercalciuria? NKCC2 activity is modulated by the calcium sensing receptor (CaSR) via phosphorylation^56^, and calcilytics (CaSR antagonists), well tolerated but disappointing agents in their utility for treating terrestrial osteoporosis^57^, might be one potential avenue to manipulate NKCC2 activity to ameliorate spaceflight induced hypercalciuria.

Abnormal water homeostasis has been noted in astronauts since the early days of spaceflight, with a marked reduction in both diuresis and natriuresis with vasopressin dysregulation via unknown mechanisms^1–3^. In fact, the reduced NKCC2 activity would conversely be anticipated to enhance diuresis, much like a loop diuretic. In this context, downregulation of prostaglandin transporters (PGT; encoded by *SLCO2A1*), a result seen in multiple of our proteomic and transcriptomic datasets (Fig. 5), is intriguing and may offer a way to reconcile this paradox. As carriers of a gain-of-function mutation of *SLCO2A1*^58^, see a reduction in PGT activity in the proximal straight tubule and the cortical collecting duct which colocalises with Aquaporin-2 (*AQP2*). Loss of PGT-mediated reabsorption by collecting duct principal cells, causes a subsequent build-up in the luminal concentration of the prostaglandin PGE_2_, which stimulates apical prostaglandin E_2_ receptor 4 (EP_4_) receptors to activate Aquaporin-2 and inappropriate water reabsorption. In our study the loss of NKCC2 activity through dephosphorylation and reduction of PGT abundance can offset each other.

Tubular remodelling in spaceflight raises another, interesting question; is the hypercalciuria seen in spaceflight a consequence or cause of spaceflight related loss of bone mineral density (BMD)? We know that primary renal phenomena associated with tubular remodelling (e.g. genetic inactivation of *SLC12A1* in Bartter syndrome^59^ or chronic furosemide exposure^60^) can cause a loss of BMD as a consequence of the primary hypercalciuria. This implies that renally targeted therapies (e.g. thiazides, calcilytics) will be effective. There does appear to be a synergistic effect of microgravity and GCR on BMD loss; GCR does have primary bone effects, increasing osteoclastogenesis and bone loss in rodents^16^, so bone targeted countermeasures will still be relevant.

The kidney is a key microvascular end-organ and microvascular disease appears to be one of the main consequences of GCR exposure^30^. We concur with this view: vascular disease and endothelial dysfunction disease ontologies were highly enriched across omics datasets (Fig. 8). Consistent with this, the arginine biosynthesis pathway was highly enriched across our human and mouse datasets (Fig. 4c), there was a significantly increased amount of pathogenic miR-125b in the highly vascular ISOM (Fig. 9b) and as a likely consequence of this microvascular pathology, we observed incidences of thrombotic microangiopathy on histopathological examination (Fig. 10a).

In addition to this, pathophysiological macrovascular changes are a well-documented consequence of microgravity, with 6 months in space shown to induce ~ 20 years of vascular ageing through arterial stiffening^61^. The resultant loss of vascular elasticity, and thus buffering capacity, leads to elevated transmission of blood flow and pressure pulsatility to the microvasculature. Long-term, this can cause barotrauma or oxidative stress to renal microvasculature due to suboptimal perfusion, especially as the myogenic response of the afferent arteriole is to vasoconstrict and provide resistance (and thus reduce flow) in the face of excessive pulsatile pressure^62^. Indeed arterial stiffness and CKD are strongly associated, and it is thought that one begets the other in a positive feedback loop^63^. Taken together with the general drop in blood pressure experienced by astronauts^64^, exposure to spaceflight long-term places the kidney in an impossible position whereby it must choose whether to defend renal perfusion or the health of its microvasculature. If the arterioles dilate to enhance blood flow, they leave the microvasculature open to damage from unbuffered pressure waves on top of the radiation damage caused by GCR. Whereas if they constrict to provide protection to the microvasculature, they reduce blood flow and therefore oxygen supply to the renal tubules, which further compounds and exacerbates the chronic oxidative stress and mitochondrial dysfunction from GCR bombardment.

This study is the largest to ever look at the effect of spaceflight on kidney function. We have demonstrated that there is renal structural and functional remodelling likely caused by microgravity, probably synergistically with GCR. We have shown that this remodelling is a potential driver of kidney stone formation and many of the changes in the urinary biochemistry of humans and animals experienced by those exposed to LEO spaceflight. We have also shown that acute exposure to simulated GCR causes both acute and chronic tubular epithelial and vascular damage that appears both progressive and irreversible.

However, our study has several limitations. We have necessarily relied on human (mostly male) and animal (mostly female) data from missions which were exposed to microgravity for relatively short periods of time compared to the projected length of a Mars mission, and only in LEO, which also limited the amount of GCR and other space radiation that they were exposed to. Additionally, our simulated GCR experiments only looked at acute unfractionated dosing, which may not accurately model the chronic cumulative exposure that would happen on a Mars mission.

Therefore, in the absence of experiments that can more accurately model the long-term simultaneous exposure to full GCR and microgravity over several years, and the plausible synergistic impact of these insults, our results should be taken a conservative glimpse into the potential dire health consequences of long-term deep space travel. Much more intensive study of the health impacts of spaceflight on the kidneys and other visceral organs that have received little attention is of the utmost importance if we are to develop mitigation strategies and send humans to other planets and beyond.

## Methods

### Human studies ethical approval

All crews from the Inspiration4, NASA, JAXA spaceflights provided informed written consent prior to their participation.

Inspiration4 subjects were consented at an informed consent briefing (ICB) at SpaceX (Hawthorne, CA), and samples were collected and processed under the approval of the Institutional Review Board (IRB) at Weill Cornell Medicine, under Protocol 21-05023569. All crew members have consented for data and sample sharing. Tissue samples were provided by SpaceX Inspiration4 crew members after consent for research use of the biopsies, swabs, and biological materials.

The procedure followed guidelines set by the Health Insurance Portability and Accountability Act (HIPAA) and operated under Institutional Review Board (IRB) approved protocols. Experiments were conducted in accordance with local regulations and with the approval of the IRB at Weill Cornell Medicine (IRB #21-05023569). NASA IRB (CSA defers to NASA’s IRB), ESA IRB, JAXA IRB for their respective crewmembers from Biochemical Profile (NASA IRB Pro0797) and Nutritional Status Assessment: SMO 016E (pro0326) projects. All crews provided informed written consent prior to participation.

The JAXA human spaceflight study was proposed to and supported by the 2014 International Life Sciences Research Announcements, JAXA, and NASA. Ethics committee approvals were obtained at the University of Tsukuba (No. 251, Nov. 27, 2015), JAXA (JX-IRBA-20-071, Aug. 30, 2016), NASA (Pro1995, Feb. 28, 2017), ESA (2017_04_09, Apr. 20, 2017). Informed consent was obtained by the personal information manager of the study, and de-identified samples were made available to researchers who performed sample processing and data analysis.

Cosmonaut data were obtained from previously published data [https://link.springer.com/article/10.1007/s10517-013-2310-2].

### Animal Studies ethical approval

For all animal data coming from NASA Genelab Open Science Data Repository (see detailed citations for all missions in the supplementary methods section) the ethical oversight information and protocol number can be found in the Protocol section (e.g. https://osdr.nasa.gov/bio/repo/data/studies/OSD-102).

For BNL-1/2/3—Brookhaven National Laboratory IACUC Protocol 506 “miRNA Signature Detection and Countermeasures Against HZE Radiation Exposure for Tissue Degeneration”.

For NSRL-22A—All care and procedures were approved by the Institutional Animal Care and Use Committees (IACUC) at BNL and CHOP and were in accordance with the AAALAC and National Institute of Health (NIH) guidelines for the care and use of laboratory animals.

For RR-10- NASA John F. Kennedy Space Center Institutional Animal Care and Use Committee (IACUC) Research Protocol Review IACUC Protocol #: FLT-20-133 “The Role of CDKN1a/p21Pathway in Microgravity-Induced Bone Tissue Regenerative Arrest—A Spaceflight Study of Transgenic CDKN1a/p21-Null Mice in Microgravity (SpaceX-21)”

### Sex differences

For the human studies: NASA historical data is pooled and anonymised for ethical reasons.

Cosmonaut and JAXA data is all male. SpaceX data involves both sexes, but this cannot be disaggregated for ethical reasons of anonymity.

For the animal studies: The findings apply mostly female animals (roughly two thirds of animal missions were female). As we were collecting data from previously conducted experiments, we did not have the ability to alter this sex disparity. Only one study had both males and females, which enabled us to perform sex difference analyses for parameters such as blood and urine electrolytes.

### Sample sizes

Due to the nature of our study we used samples from animals that had been in spaceflight (usually as part of the ‘Rodent Research’ missions to the International Space Station) or had been taken from costly experiments using the Galactic Cosmic Radiation Simulator at Brookhaven national laboratories. We also used samples and/or data from humans who had undergone spaceflight (in historical NASA missions, Roscosmos missions, and JAXA missions to the ISS or modern commercial missions with SpaceX such as inspiration4). Our sample numbers were therefore limited to the number of samples made available to us in the original experimental designs or mission parameter limitations (restricted by practical considerations of weight to launch, available space and resource consumption). In all cases, we endeavoured to use the maximum number of biological replicates available to us to maximise experimental power.

### Statistics and reproducibility

#### Omics studies

This study encompassed 2 epigenomic (Whole-genome bisulfite sequencing), 9 transcriptomic (8 Bulk RNA sequencing, 1 Bulk small RNA sequencing), 7 proteomic (3 Quantitative fractionated TMT-labelled DDA LC-MS/MS, 2 Quantitative label-free SEER DIA LC-MS/MS, 1 Plasma Slow Off-Rate Modified Aptamers, 1 Shotgun LC-MS/MS), 1 epiproteomic (Quantitative fractionated TMT-labelled DDA LC-MS/MS of phospho-enriched peptides), 3 metabolomic (Quantitative label-free UHPLC-MS/MS) and 6 metagenomic (4 Whole metagenome shotgun sequencing, 2 16S rRNA gene amplicon sequencing) studies on both humans and animals. Full details of the experimental, processing and analytical methodologies and locations of publicly available raw data are given in the supplementary methods.

#### Physiology

##### Sample collection and processing

*NSRL-22A*

Urine collection was performed in awake mice to obtain spontaneous and uncontaminated samples. Mice were gently handled over a sheet of Saran® wrap or Parafilm® to facilitate micturition. Upon collection, each urine sample was carefully divided into two aliquots. One aliquot was immediately acidified with HNO3 to achieve a final concentration of 1% v/v, preventing precipitation of electrolytes and maintaining sample integrity. Both aliquots were then promptly stored at − 80 °C to preserve their biochemical constituents until further analysis. For blood collection, mice were subjected to terminal anaesthesia to minimise distress and ensure compliance with ethical guidelines. Blood samples were drawn from the vena cava using a fine needle and syringe. Plasma was separated by transferring the blood into Microvette® CB 300 LH (Sarstedt) tubes and centrifuging at 2000 *g* for 5 min. The resulting plasma supernatant was carefully aspirated and stored at − 80 °C for future analysis. Both plasma and non-acidified urine samples were analysed for creatinine levels by employing either the standard Jaffe reaction or the enzymatic method, depending on the investigator’s preference and laboratory resources. Electrolyte levels, biochemistries and biomarkers were assessed using the Siemens Dimension RxL Max Integrated Chemistry System, at the Core Biochemical Assay Laboratory at Addenbrooke’s Hospital, Cambridge, UK.

*Inspiration4*

Plasma samples collected from astronauts at timepoints L-92d, L-44d and L-3d were grouped and averaged into “pre-flight” for comparison against individual post-flight timepoints R + 1d, R + 45d, R + 82d and R + 194d. These samples were analysed by Quest Diagnostics using the Comprehensive metabolic panel [CMP; CPT code 80053].

*NASA*

A historical collection of archived astronaut frozen plasma and urine aliquots were collected and stored at − 80 °C over many years. Clinical chemistries, endocrine profiles and biomarkers were obtained at the Nutritional Biochemistry Laboratory at NASA Johnson Space Center. Timepoints L-180d and L-45d were grouped and averaged as “pre-flight” for comparison to individual timepoints L + 15d, L + 30d, L + 60d, L + 120d, L + 180d, R + 0d and R + 30d.

##### Functional calculations

Functional calculations used in the data analysis are fully detailed in the supplementary methods.

#### Imaging

##### Sample fixation and processing

Mouse kidney samples were harvested and fixed through immersion in freshly prepared 4% (w/v) formaldehyde-PBS solution (pH 6.9) for 16 h at 37 °C. Subsequently, the samples were washed three times with PBS with 0.02% Na-Azide and stored at 4 °C until they were embedded in paraffin.

##### Histopathology and Brightfield WSI

2-3 µm FFPE sections from NSRL-22A kidneys were prepared, deparaffinised using Histoclear (National Diagnostics), and rehydrated through a series of graded methanol steps. These were then stained for either Haematoxylin and Eosin (#ab245880, Abcam) or Masson’s Trichrome (#ab150686, Abcam) according to the manufacturers guidelines.

Sections underwent brightfield whole slide imaging with 20X objective tiled and stitched focus stacks taken for each section on a Zeiss Axio Scan.Z1 slide scanner. The resultant images were viewed in QuPath (v0.4.3) and semi-quantitatively scored for any histopathological findings.

##### Immunostaining and confocal WSI

5 µm FFPE sections from RR-10 kidneys were prepared, deparaffinised using Histoclear (National Diagnostics), and rehydrated through a series of graded methanol steps. Antigen retrieval was performed using [1X] R-Universal buffer (AP0530) in a 2100 antigen retriever for a single heat-pressure cycle (Aptum Biologics). Sections were then permeabilized with 0.05% (v/v) Triton X-100-PBS solution for 20 minutes and incubated with Section Block ‘ready-to-use’ (AP0471; Aptum Biologics) for 30 mins at RT. Primary antibodies were incubated overnight at 4 °C for 16 hours at specified concentrations (see supplementary methods), diluted in Antibody Diluent ‘FF/PE Sections’ (AP0472; Aptum Biologics). For phospho-specific antibodies, 10 μg/ml of the non-phospho peptide used to raise the antibody was added per 2 μg/ml of the antibody used. Negative control samples omitted the primary antibody and were processed simultaneously. Following incubation, slides were washed by dipping slides 50x times in 0.05% (v/v) Triton X-100-PBS for 3 rounds, and incubated with secondary antibodies for 1 hour at RT. Pre-absorbed fluorochrome conjugated secondary antibodies were utilised at specified concentrations (concentrations (see supplementary methods), diluted in Antibody Diluent ‘FF/PE Sections’ (AP0472; Aptum Biologics). After washing, slides were mounted using Prolong Gold antifade (#P36930, Life Technologies), permitted to cure for 48-72hrs, sealed with CoverGrip Coverslip Sealant (#23005; Biotium) and protected from light exposure.

Immunofluorescent images were captured using either a Zeiss LSM700 LED laser-scanning confocal microscope or Leica SP8 white-light laser-scanning confocal microscope using 488-nm, 555-nm and 639-nm laser lines, employing a 10X/0.3NA (Zeiss) or 10X/0.5NA (Leica) objective. Zeiss acquisition parameters included single field of view, 8-bit resolution, 1844 x 1844 pixels, 1x digital zoom, 3.85µs pixel dwell time, 4-line Kalman filtering, sequential (by line) channel imaging, and a 4-slice z-stack with a 6 µm thickness to account for chromatic aberration.

Leica acquisition parameters included tile scanning with a 10% overlap, 8-bit resolution, 1024 × 1024 pixels, 0.75x digital zoom, 600 Hz scan speed, 6-line Kalman filtering, sequential (by line) channel imaging, and a 3-slice z-stack with a 5 µm thickness to account for chromatic aberration.

Images were processed using FIJI image analysis software (v.1.53p). Fluorescent z-stacks underwent background subtraction (200 px radius rolling ball) and maximum intensity z-projection. Brightness and contrast adjustments were made using linear histogram stretching to enhance visibility.

##### miRNA in situ hybridisation and brightfield WSI

FFPE blocks for BNL-3 kidneys were trimmed to remove tissue exposed to air, and fresh 5 µm sections were taken. These were not baked onto the slide and stored with desiccant instead. Samples were then processed using the miRNAscope™ HD RED assay (#324531 and #324500, ACD-Biotechne) with the FFPE tissue section workflow according to the manufacturers guidelines. miRNAscope™ Probes against the following were used: mmu-miR-125b-5p (#1082311-S1), mmu-let-7a-5p (#727761-S1) and mmu-miR-16-5p (729031-S1).

Sections underwent brightfield whole slide imaging with 20X objective tiled and stitched focus stacks (with extended depth of focus) taken for each section on a Zeiss Axio Scan.Z1 slide scanner. Using QuPath (v0.4.3) the cortex, outer stripe of the outer medulla, inner stripe of the outer medulla and inner medulla were manually annotated with the aid of a Wacom cintiq pro 32 to create a segmentation mask of the anatomical regions for area calculation. Ilastik (v1.4.0)^65^ automated (supervised) pixel-level classification was then trained to identify positive miRNAscope probe staining. A custom in-house python script was then used to count all positive staining and calculate the area of all anatomical regions, so that the density of each miRNA per unit area of each region could be determined for each kidney section.

##### Optical clearing and 3D imaging

Tissue transformation, delipidation and refractive index matching

Quartered formaldehyde-fixed kidneys from RR-10 mice were tissue transformed using the SHIELD protocol^66^ and reagents from Lifecanvas technologies (MA, USA) based on (10.1038/nbt.4281). SHIELD-transformed kidneys were delipidated (Lifecanvas Technologies; Full Passive Pipeline Protocol v4.06) for approximately 10–14 days at 37 °C in 40 mL of passive delipidation buffer with gentle agitation in EasyClear device. Following delipidation, tissues were washed in several rounds of PBS at 37˚C overnight to remove any delipidation buffer. Before imaging tissues were immersed in Incubated in EasyIndex RI 1.53 refractive index matching solution as per protocol. Tissues were then embedded in 2% w/v ultra-low melting point agarose (Sigma A5030) blocks made up with EasyIndex solution to immobilise the samples. These were then stored in EasyIndex in airtight containers shielded from light prior to imaging.

MesoSPIM imaging

Samples were mounted in quartz cuvettes and immersion oil with refractive indices matching that of EasyIndex, and then suspended and aligned for light-sheet fluorescence imaging with a MesoSPIM^67^. Images were captured at 16-bit for 1-channel dual laser lightsheet illumination (488 nm excitations) to obtain autofluorescence emissions at 2048 x 2048 pixels in the XY, with a Z depth range of 700-1200 pixels, giving a pixel resolution of 3.26 μm (XY) and 4.0 μm (Z).

Image analysis

To evaluate any qualitative changes in gross morphology images were imported into Syglass^68^ (v.1.7.2-79; https://www.syglass.io/; RRID: SCR_017961) for visual investigation by nephrologists and histopathologists in 3D virtual space using Meta Quest 2 VR headsets. Images were also imported into Imaris^69^(v10.0) for visualisation on a Wacom cintiq pro 32” 4 K touchscreen monitor. 3D video renders were later generated with Syglass (v.2.0.0) and Z-slice video created with FIJI (ImageJ; v.1.54 h).

#### Morphometry

##### Normalised kidney weights

PI and NASA GeneLab records for all animals included in the study were examined for bodyweight and kidney wet weight measurements. Only BNL-1, BNL-2, BNL-3 and RR-23 missions had complete kidney and body weight information available. Where available, the weights of both left and right kidneys were averaged, and the kidney weights were normalised against bodyweight for the same animal and expressed as a percentage. All ground control and sham animals that received no exposure treatment were grouped into control for pairwise comparisons against GCR (animals that only received either full or simplified galactic cosmic radiation simulations) or MG (animals that only underwent hindlimb unloading microgravity simulation) or GCR + MG (animals that underwent a combination of GCR and MG or were exposed to spaceflight).

##### Histomorphometry

Whole slide images of tiled immunofluorescent confocal images taken from RR-10 were used for analysis. Using QuPath (v0.4.3) the cortex was manually annotated with the aid of a Wacom cintiq pro 32 to create a segmentation mask of the anatomical region for area calculation. Similarly distal convoluted tubules (DCT) labelled with antibodies against total NCC / phospho-NCC (see supplementary methods for details), the canonical DCT marker, were annotated. Initially, only a handful of tubules were annotated from each slide, and these were then used to train Ilastik (v1.4.0)^65^ for automated (supervised) pixel-level classification of DCTs. These were then further manually refined to remove/include any false-positive/negatives. A custom in-house python script was then used to compute the number of discrete tubules positive for DCT markers, the corresponding area of each of these as well as the total cortex area, such that average tubule area and DCT density per area of cortex could be determined for each kidney section.

### Reporting summary

Further information on research design is available in the Nature Portfolio Reporting Summary linked to this article.

### Supplementary information

Supplementary Information

Peer Review File

Description of Additional Supplementary Files

Supplementary Data 1

Supplementary Data 2

Supplementary Data 3

Supplementary Data 4

Supplementary Data 5

Supplementary Data 6

Supplementary Data 7

Supplementary Data 8

Supplementary Data 9

Supplementary Data 10

Supplementary Movie 1

Supplementary Movie 2

Reporting Summary

### Source data

Source Data

## Data Availability

Figure 1a, b Aggregated from NASA OSDR Fig. 2. a, b and Supplementary Fig. 1 A, B. The data included in these figures are available under restricted access and deidentified in order to preserve participants anonymity, access can be obtained by contacting Scott Smith (scott.m.smith@nasa.gov). Figure 3a, b, c; Fig. 7b, c; Supplementary Fig. 6; Supplementary Data 1,Supplementary Movie 1,2) underlying data from NASA OSDR: OSD-462 (10.26030/8g1a-3041) Fig. 4a; Fig. 5; Fig. 6a, b; Fig. 8; Supplementary Figs. 2,4,5,7; Supplementary Data 2,3,6,7) underlying datasets from NASA OSDR: OSD-102 (10.26030/yn9m-2d19), 163 (10.26030/q8vt-7p92), 253 (10.26030/4mx6-5x80), 336 (10.26030/qasa-rr29), 342 (10.26030/v2ak-0y21), 462 (10.26030/8g1a-3041), 513 (10.26030/pprb-6227), 457 (10.26030/yyce-8y73), 530 (10.26030/r2xr-h714), 532 (10.26030/j15f-vj38), 571 (10.26030/h3p5-tc29), 708 (10.26030/r4pv-hw21), 709 (10.26030/fhs1-z519) Fig. 4b; Supplementary Fig. 3,7; Supplementary data 4) underlying datasets from NASA OSDR: OSD-72 (10.26030/qyw7-qn34), 212 (10.26030/8vac-wb94), 249 (10.26030/h713-bd02), 250 (10.26030/rj1y-dq03), 465 (10.26030/vqhs-qq63), 466 (10.26030/6axn-0058). Figure 4c; Supplementary Fig. 7; Supplementary Data 5) Rat data at request of CNSA authors (10.3389/fphys.2020.00939), MHU-3 data at request from authors (https://ibsls.megabank.tohoku.ac.jp/metabolite-list)^70,71^, NASA OSDR: OSD-571 (10.26030/h3p5-tc29) Fig. 6c) data available from Peptide Atlas (https://peptideatlas.org/). Dataset identifier: PASS00239 Fig. 7a) kidney and bodyweight data are available on request to abehesht@broadinstitute.org for BNL-11213 mice and to valery.boyko@nasa.gov and NASA OSDR: OSD-513 (10.26030/pprb-6227), 462 (10.26030/8g1a-3041), 710 (10.26030/r1hh-ev67), 712 (10.26030/f6b9-4093), 709 (10.26030/fhs1-z519) Fig. 9b; Supplementary Fig. 9) underlying datasets from NASA OSDR: OSD-710 (10.26030/r1hh-ev67) Fig. 9d; Fig. 10A; Supplementary Data 10) underlying datasets from NASA OSDR: OSD-708 (10.26030/r4pv-hw21) Fig. 10b) underlying datasets from NASA OSDR: OSD- 706 (10.26030/rrwe-h429), 707 (10.26030/bcnk-5z50) Supplementary Fig. 1C) underlying datasets from NASA OSDR: O5D-575 (10.26030/mc5d-p710) Supplementary Fig. 7 Comprises all of the other data sets listed here. Supplementary Fig. 8; Supplementary Data 8,9) underlying datasets from NASA OSDR: OSD-336 (10.26030/qasa-rr29) For further information see Supplementary Information. Source data are provided with this paper.

## Code availability

Scripts used for the analysis in this manuscript can be found at the following GitHub sites: https://github.com/nestlerk/Kidney_Spaceflight, https://github.com/luslab/spatial-space-kidney and at https://github.com/ZWL-S/Cosmic-Kidney-Disease_LTC.
