# Supplementary material for: Cosmic kidney disease: an integrated pan-omic, physiological and morphological study into spaceflight-induced renal dysfunction
Source: Nat Commun. 2024 Jun 11;15:4923. doi: 10.1038/s41467-024-49212-1 (PMC11167060; doi:10.1038/s41467-024-49212-1)
Supplement: Supplementary file 1 — Supplementary Information [file 41467_2024_49212_MOESM1_ESM.pdf]

Supplementary Table 1. Exact p-values for figure 2.

URINE

Adjusted P-value	FE Ca	Oxalate	Uric Acid	Phosphate	Volume	Citrate	Sodium	Magnesium	TmP/GFR	pH	Osmolality	Free Water Clearance
**FD15**	0.003370	>0.999999	0.056845	0.002058	0.000821	0.810013	>0.999999	0.000048	0.024698	>0.999999	0.079428	0.004040
**FD30**	0.000140	>0.999999	>0.999999	0.057221	0.000509	0.066749	>0.999999	0.024692	0.000111	>0.999999	0.125996	0.013957
**FD60**	0.004693	0.754059	0.343394	0.010473	0.097799	0.426127	>0.999999	0.018395	0.006865	>0.999999	0.360883	0.021602
**FD120**	0.806656	0.342796	>0.999999	0.032661	0.559921	>0.999999	>0.999999	0.016368	0.000555	>0.999999	0.860033	0.213379
**FD180**	>0.999999	0.017162	0.026069	0.000329	0.514957	>0.999999	>0.999999	0.027750	0.001692	>0.999999	0.236701	0.037563
**R+0**	>0.999999	>0.999999	0.015445	0.000002	0.505191	>0.999999	0.000568	0.007798	0.000711	>0.999999	>0.999999	>0.999999
**R+1**		>0.999999								0.019312		
**R+30**	>0.999999	>0.999999	>0.999999	>0.999999	0.866086	>0.999999	>0.999999	>0.999999	>0.999999	>0.999999	>0.999999	>0.999999
**R+31**		>0.999999								>0.999999		
**ANOVA summary**	<0.0001	0.001424	<0.000001	<0.000001	0.001594	0.014056	0.000034	<0.000001	0.000011	0.012060	0.008156	0.000023

BLOOD

Adjusted P-value	1,25-Dihydroxyvitamin D3	PTH	FGF-23	Renin	Aldosterone	Atrial Natriuretic Peptide
**FD15**	>0.999999	0.015767	>0.999999	0.011353	>0.999999	0.033014
**FD30**	0.013625	0.006488	>0.999999	0.002332	0.881279	0.003087
**FD60**	0.528434	0.133337	>0.999999	0.036261	>0.999999	0.003087
**FD120**	0.319539	0.822117	>0.999999	0.008913	>0.999999	0.000194
**FD180**	>0.999999	>0.999999	>0.999999	0.006545	>0.999999	0.015679
**R+0**	0.000028	>0.999999	0.198271	>0.999999	>0.999999	>0.999999
**R+1**	>0.999999					
**R+30**		<0.000001	>0.999999	>0.999999	>0.999999	>0.999999
**ANOVA summary**	<0.000001	<0.000001	0.075601	0.000181	0.207730	<0.000001

Supplementary Figure 1: Human plasma and urine physiological measurements

a, Urinary and **b,** plasma physiological measurements from NASA astronauts (n=66) exposed to spaceflight up to 180 days and **c,** plasma physiological measurements from Inspiration4 SpaceX astronauts exposed to spaceflight for 3 days. Values were measured pre-flight, during (FD = flight day) and after returning (R). Dashed lines represent upper and lower normal clinical values, or upper limit where only a single line is present. Data are presented as mean ± SD. Boxed P-values report the repeated measure one-way ANOVA result; all timepoints were compared to pre-flight by pairwise multiple comparison Bonferroni corrected post-hoc tests (* $p < 0.05$, ** $p < 0.01$, *** $p < 0.001$, **** $p < 0.0001$). FE; fraction excretion. TTKG; trans-tubular potassium gradient. Corrected; corrected for albumin. Na; sodium. K; potassium. Mg; magnesium. Cl; chloride. P_{O4}; phosphate. H2O; water. eGFR; estimated glomerular filtration rate corrected for body surface area (BSA). 25-hydroxyvitamin D3; calcifediol. BUN; blood urea nitrogen.

a

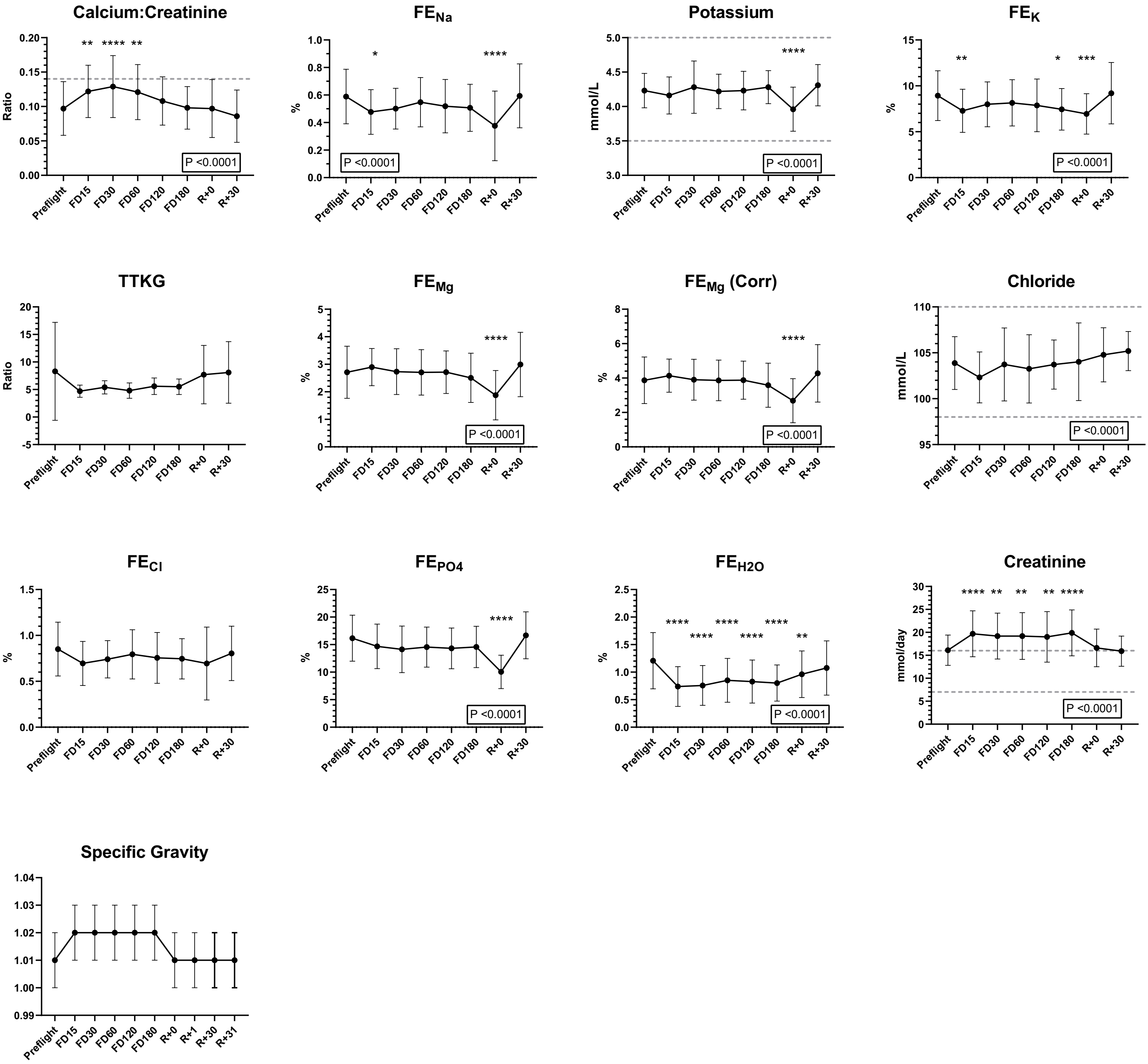
Calcium:Creatinine
Ratio
P <0.0001
FE$_{Na}$
%
P <0.0001
Potassium
mmol/L
P <0.0001
FE$_{K}$
%
P <0.0001
TTKG
Ratio
FE$_{Mg}$
%
P <0.0001
FE$_{Mg}$ (Corr)
%
P <0.0001
Chloride
mmol/L
P <0.0001
FE$_{Cl}$
%
FE$_{PO4}$
%
P <0.0001
FE$_{H2O}$
%
P <0.0001
Creatinine
mmol/day
P <0.0001
Specific Gravity
Preflight
FD15
FD30
FD60
FD120
FD180
R+0
R+1
R+30
R+31

b

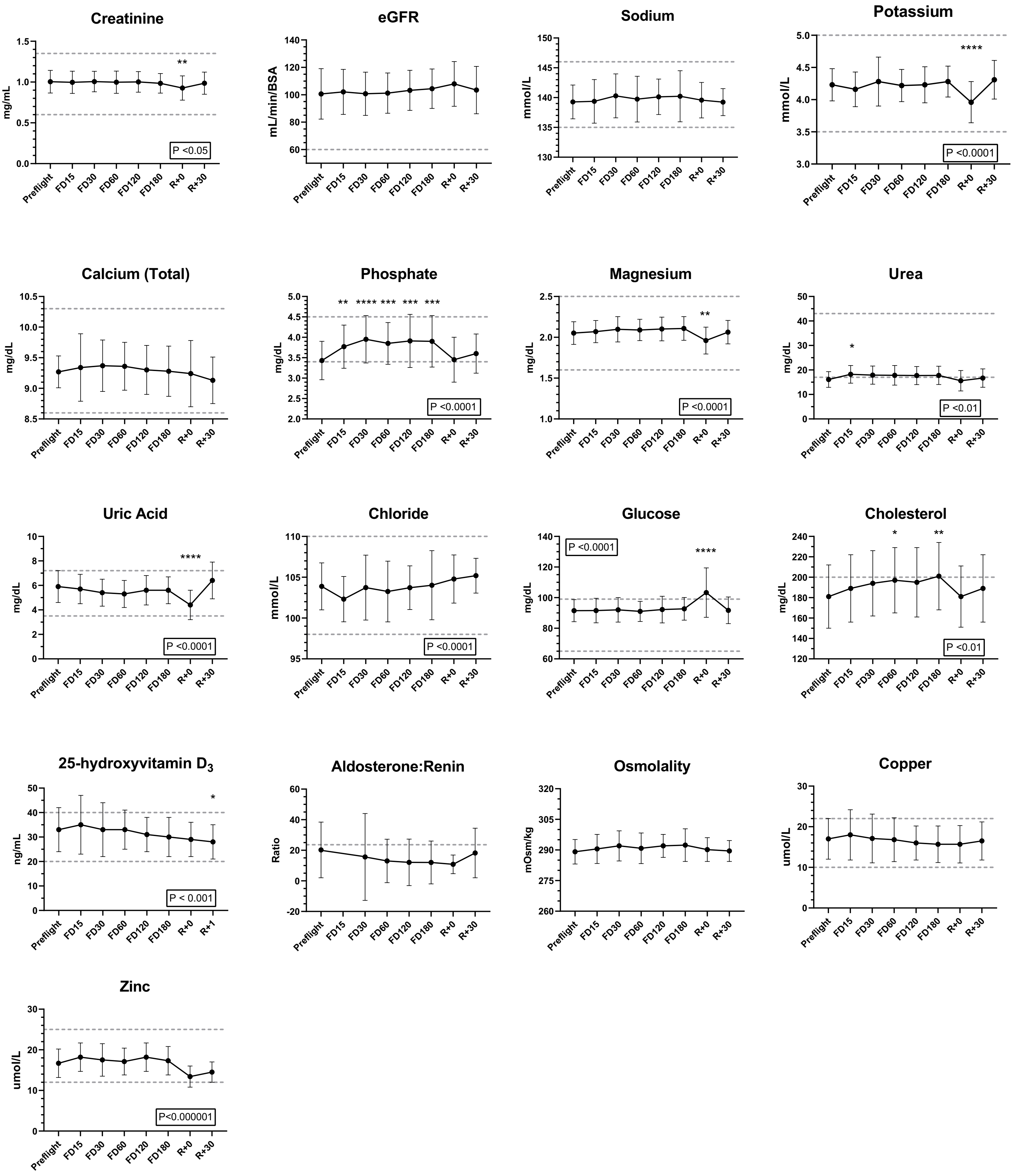

Creatinine
mg/mL
P <0.05
**
eGFR
mL/min/BSA
Sodium
mmol/L
Potassium
mmol/L
****
P <0.0001
Calcium (Total)
mg/dL
Phosphate
mg/dL
** **** *** *** ***
P <0.0001
Magnesium
mg/dL
**
P <0.0001
Urea
mg/dL
*
P <0.01
Uric Acid
mg/dL
****
P <0.0001
Chloride
mmol/L
P <0.0001
Glucose
mg/dL
P <0.0001
****
Cholesterol
mg/dL
* **
P <0.01
25-hydroxyvitamin D3
ng/mL
*
P < 0.001
Aldosterone:Renin
Ratio
Osmolality
mOsm/kg
Copper
umol/L
Zinc
umol/L
P<0.000001
Preflight
FD15
FD30
FD60
FD120
FD180
R+0
R+30
R+1

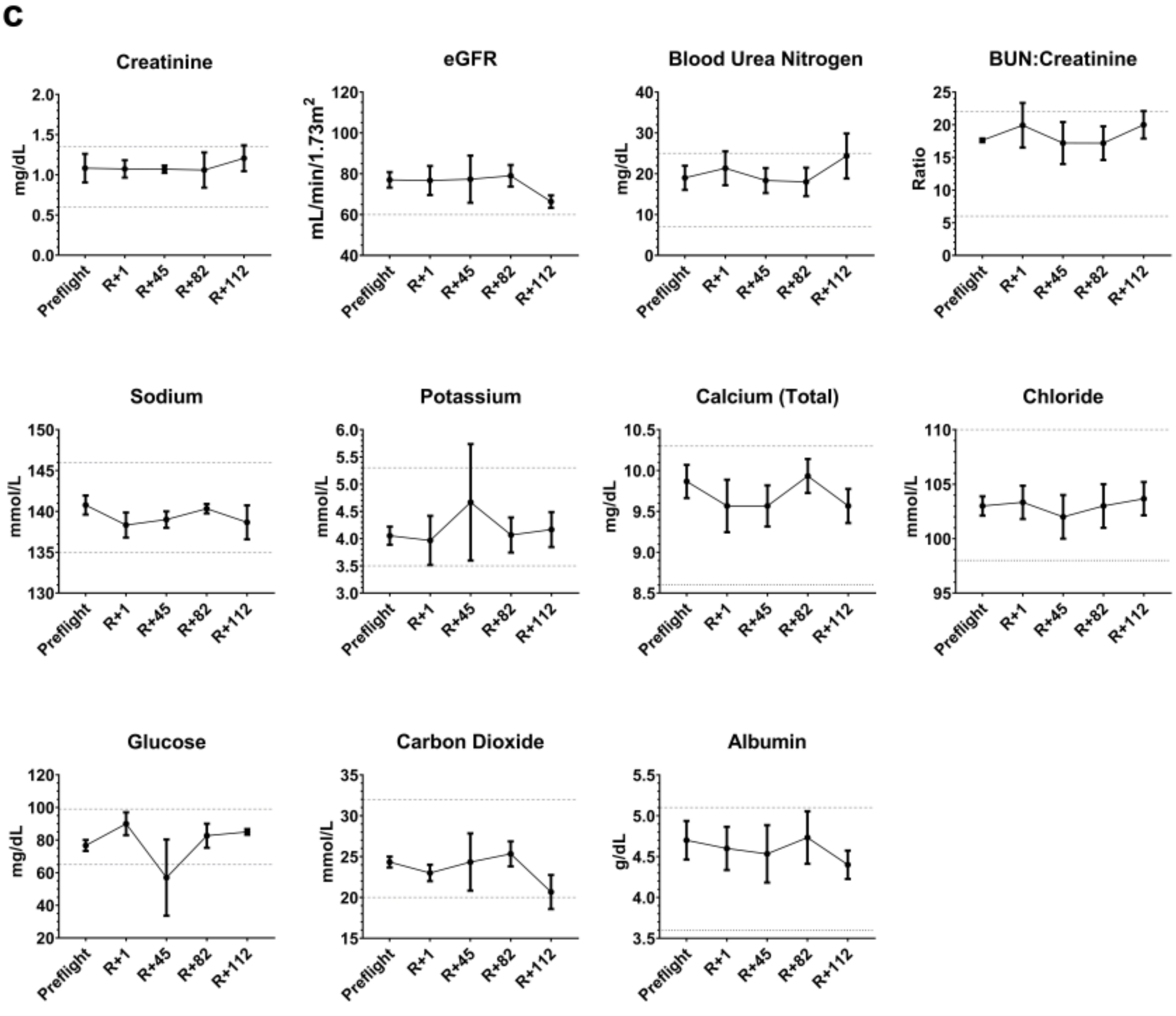
c
Creatinine
mg/dL
2.0
1.5
1.0
0.5
0.0
eGFR
mL/min/1.73m2
120
100
80
60
40
Blood Urea Nitrogen
mg/dL
40
30
20
10
0
BUN:Creatinine
Ratio
25
20
15
10
5
0
Sodium
mmol/L
150
145
140
135
130
Potassium
mmol/L
6.0
5.5
5.0
4.5
4.0
3.5
3.0
Calcium (Total)
mg/dL
10.5
10.0
9.5
9.0
8.5
Chloride
mmol/L
110
105
100
95
Glucose
mg/dL
120
100
80
60
40
20
Carbon Dioxide
mmol/L
35
30
25
20
15
Albumin
g/dL
5.5
5.0
4.5
4.0
3.5
Preflight
R+1
R+45
R+82
R+112

Supplementary Figure 2: Multi-omic DisGeNET over-representation analysis top 100 results by category grouping

The top 100 enriched gene-disease associations are presented for DisGeNET ontological terms relating to **a**, kidney health [kidney; urological; electrolyte; mitochondrial (general); complement; urinary system cancers; blood pressure (renal)], **b**, closely related to kidney injury and disease [Vascular/endothelial; cardiovascular; autonomic dysfunction; diabetes; blood pressure (general)], **c**, general diseases known to impact the kidney [non-urinary systems cancers; aging; inflammation; liver], **d**, no clear connection to the kidney. These were ranked and represented in descending order using the following rules: 1) No. of mission datasets it replicated in; 2) most significant p-value; 3) greatest enrichment. To integrate datasets from different omics modalities, species, missions and tissues, all biomolecules (e.g. phosphopeptides, proteins, transcripts and methylated DNA) were converted to the human orthologs where necessary and linked back to their HGNC gene symbol, aggregated and collapsed to single genes (e.g. multiple phosphosites, isoforms, CpG sites). A Log10(P-value) of 2 was considered significant for ontological term enrichment. Enrichment ratio; the number of differentially regulated hits in a dataset that belong to a given ontological term, normalised to the total number of statistically significant hits in the respective dataset.

a

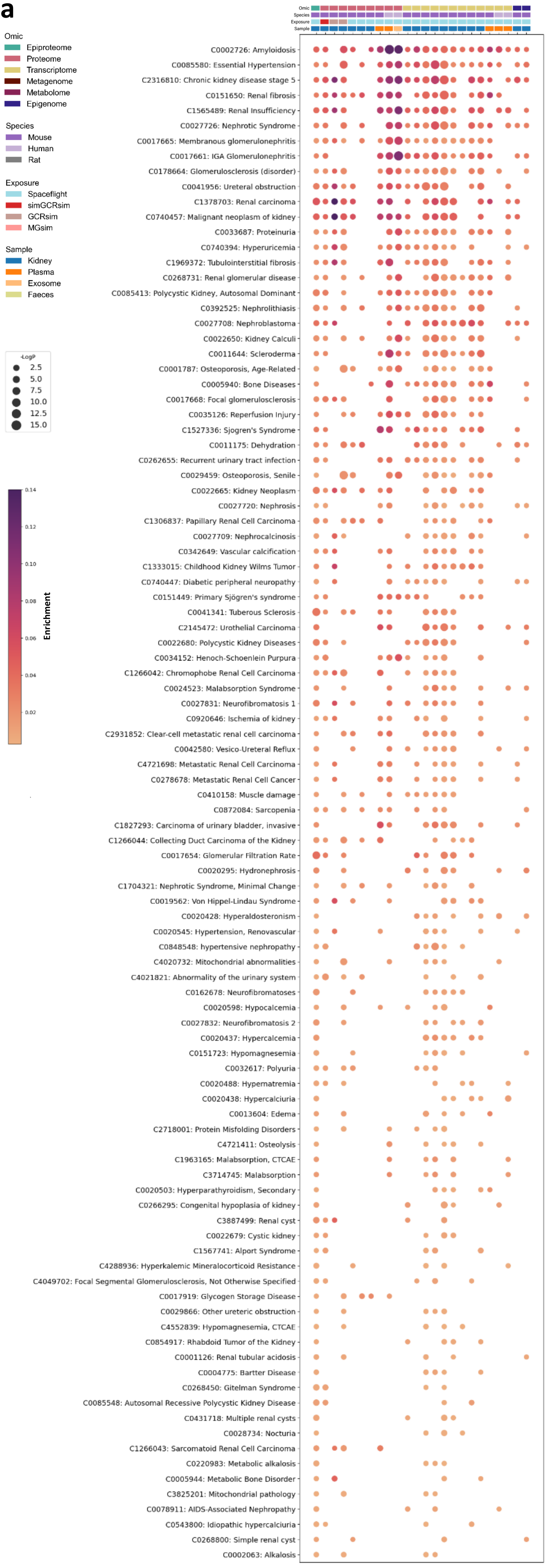
Omic
Species
Exposure
Sample
Omic
Epiproteome
Proteome
Transcriptome
Metagenome
Metabolome
Epigenome
Species
Mouse
Human
Rat
Exposure
Spaceflight
simGCRsim
GCRsim
MGsim
Sample
Kidney
Plasma
Exosome
Faeces
-LogP
2.5
5.0
7.5
10.0
12.5
15.0
Enrichment
0.14
0.12
0.10
0.08
0.06
0.04
0.02
C0002726: Amyloidosis
C0085580: Essential Hypertension
C2316810: Chronic kidney disease stage 5
C0151650: Renal fibrosis
C1565489: Renal Insufficiency
C0027726: Nephrotic Syndrome
C0017665: Membranous glomerulonephritis
C0017661: IGA Glomerulonephritis
C0178664: Glomerulosclerosis (disorder)
C0041956: Ureteral obstruction
C1378703: Renal carcinoma
C0740457: Malignant neoplasm of kidney
C0033687: Proteinuria
C0740394: Hyperuricemia
C1969372: Tubulointerstitial fibrosis
C0268731: Renal glomerular disease
C0085413: Polycystic Kidney, Autosomal Dominant
C0392525: Nephrolithiasis
C0027708: Nephroblastoma
C0022650: Kidney Calculi
C0011644: Scleroderma
C0001787: Osteoporosis, Age-Related
C0005940: Bone Diseases
C0017668: Focal glomerulosclerosis
C0035126: Reperfusion Injury
C1527336: Sjogren's Syndrome
C0011175: Dehydration
C0262655: Recurrent urinary tract infection
C0029459: Osteoporosis, Senile
C0022665: Kidney Neoplasm
C0027720: Nephrosis
C1306837: Papillary Renal Cell Carcinoma
C0027709: Nephrocalcinosis
C0342649: Vascular calcification
C1333015: Childhood Kidney Wilms Tumor
C0740447: Diabetic peripheral neuropathy
C0151449: Primary Sjögren's syndrome
C0041341: Tuberous Sclerosis
C2145472: Urothelial Carcinoma
C0022680: Polycystic Kidney Diseases
C0034152: Henoch-Schoenlein Purpura
C1266042: Chromophobe Renal Cell Carcinoma
C0024523: Malabsorption Syndrome
C0027831: Neurofibromatosis 1
C0920646: Ischemia of kidney
C2931852: Clear-cell metastatic renal cell carcinoma
C0042580: Vesico-Ureteral Reflux
C4721698: Metastatic Renal Cell Carcinoma
C0278678: Metastatic Renal Cell Cancer
C0410158: Muscle damage
C0872084: Sarcopenia
C1827293: Carcinoma of urinary bladder, invasive
C1266044: Collecting Duct Carcinoma of the Kidney
C0017654: Glomerular Filtration Rate
C0020295: Hydronephrosis
C1704321: Nephrotic Syndrome, Minimal Change
C0019562: Von Hippel-Lindau Syndrome
C0020428: Hyperaldosteronism
C0020545: Hypertension, Renovascular
C0848548: hypertensive nephropathy
C4020732: Mitochondrial abnormalities
C4021821: Abnormality of the urinary system
C0162678: Neurofibromatoses
C0020598: Hypocalcemia
C0027832: Neurofibromatosis 2
C0020437: Hypercalcemia
C0151723: Hypomagnesemia
C0032617: Polyuria
C0020488: Hypernatremia
C0020438: Hypercalciuria
C0013604: Edema
C2718001: Protein Misfolding Disorders
C4721411: Osteolysis
C1963165: Malabsorption, CTCAE
C3714745: Malabsorption
C0020503: Hyperparathyroidism, Secondary
C0266295: Congenital hypoplasia of kidney
C3887499: Renal cyst
C0022679: Cystic kidney
C1567741: Alport Syndrome
C4288936: Hyperkalemic Mineralocorticoid Resistance
C4049702: Focal Segmental Glomerulosclerosis, Not Otherwise Specified
C0017919: Glycogen Storage Disease
C0029866: Other ureteric obstruction
C4552839: Hypomagnesemia, CTCAE
C0854917: Rhabdoid Tumor of the Kidney
C0001126: Renal tubular acidosis
C0004775: Bartter Disease
C0268450: Gitelman Syndrome
C0085548: Autosomal Recessive Polycystic Kidney Disease
C0431718: Multiple renal cysts
C0028734: Nocturia
C1266043: Sarcomatoid Renal Cell Carcinoma
C0220983: Metabolic alkalosis
C0005944: Metabolic Bone Disorder
C3825201: Mitochondrial pathology
C0078911: AIDS-Associated Nephropathy
C0543800: Idiopathic hypercalciuria
C0268800: Simple renal cyst
C0002063: Alkalosis

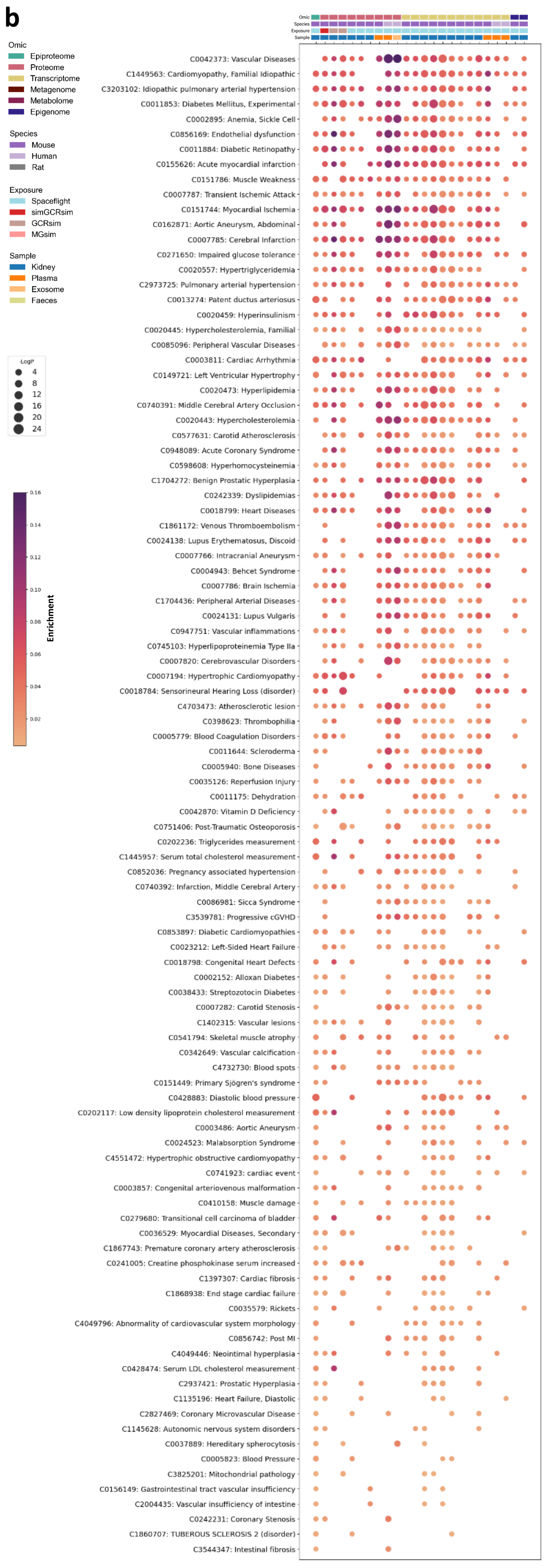
b
Omic
Epiproteome
Proteome
Transcriptome
Metagenome
Metabolome
Epigenome
Species
Mouse
Human
Rat
Exposure
Spaceflight
simGCRsim
GCRsim
MGsim
Sample
Kidney
Plasma
Exosome
Faeces
-LogP
4
8
12
16
20
24
Enrichment
0.16
0.14
0.12
0.10
0.08
0.06
0.04
0.02
Omic
Species
Exposure
Sample
C0042373: Vascular Diseases
C1449563: Cardiomyopathy, Familial Idiopathic
C3203102: Idiopathic pulmonary arterial hypertension
C0011853: Diabetes Mellitus, Experimental
C0002895: Anemia, Sickle Cell
C0856169: Endothelial dysfunction
C0011884: Diabetic Retinopathy
C0155626: Acute myocardial infarction
C0151786: Muscle Weakness
C0007787: Transient Ischemic Attack
C0151744: Myocardial Ischemia
C0162871: Aortic Aneurysm, Abdominal
C0007785: Cerebral Infarction
C0271650: Impaired glucose tolerance
C0020557: Hypertriglyceridemia
C2973725: Pulmonary arterial hypertension
C0013274: Patent ductus arteriosus
C0020459: Hyperinsulinism
C0020445: Hypercholesterolemia, Familial
C0085096: Peripheral Vascular Diseases
C0003811: Cardiac Arrhythmia
C0149721: Left Ventricular Hypertrophy
C0020473: Hyperlipidemia
C0740391: Middle Cerebral Artery Occlusion
C0020443: Hypercholesterolemia
C0577631: Carotid Atherosclerosis
C0948089: Acute Coronary Syndrome
C0598608: Hyperhomocysteinemia
C1704272: Benign Prostatic Hyperplasia
C0242339: Dyslipidemias
C0018799: Heart Diseases
C1861172: Venous Thromboembolism
C0024138: Lupus Erythematosus, Discoid
C0007766: Intracranial Aneurysm
C0004943: Behcet Syndrome
C0007786: Brain Ischemia
C1704436: Peripheral Arterial Diseases
C0024131: Lupus Vulgaris
C0947751: Vascular inflammations
C0745103: Hyperlipoproteinemia Type IIa
C0007820: Cerebrovascular Disorders
C0007194: Hypertrophic Cardiomyopathy
C0018784: Sensorineural Hearing Loss (disorder)
C4703473: Atherosclerotic lesion
C0398623: Thrombophilia
C0005779: Blood Coagulation Disorders
C0011644: Scleroderma
C0005940: Bone Diseases
C0035126: Reperfusion Injury
C0011175: Dehydration
C0042870: Vitamin D Deficiency
C0751406: Post-Traumatic Osteoporosis
C0202236: Triglycerides measurement
C1445957: Serum total cholesterol measurement
C0852036: Pregnancy associated hypertension
C0740392: Infarction, Middle Cerebral Artery
C0086981: Sicca Syndrome
C3539781: Progressive cGVHD
C0853897: Diabetic Cardiomyopathies
C0023212: Left-Sided Heart Failure
C0018798: Congenital Heart Defects
C0002152: Alloxan Diabetes
C0038433: Streptozotocin Diabetes
C0007282: Carotid Stenosis
C1402315: Vascular lesions
C0541794: Skeletal muscle atrophy
C0342649: Vascular calcification
C4732730: Blood spots
C0151449: Primary Sjögren's syndrome
C0428883: Diastolic blood pressure
C0202117: Low density lipoprotein cholesterol measurement
C0003486: Aortic Aneurysm
C0024523: Malabsorption Syndrome
C4551472: Hypertrophic obstructive cardiomyopathy
C0741923: cardiac event
C0003857: Congenital arteriovenous malformation
C0410158: Muscle damage
C0279680: Transitional cell carcinoma of bladder
C0036529: Myocardial Diseases, Secondary
C1867743: Premature coronary artery atherosclerosis
C0241005: Creatine phosphokinase serum increased
C1397307: Cardiac fibrosis
C1868938: End stage cardiac failure
C0035579: Rickets
C4049796: Abnormality of cardiovascular system morphology
C0856742: Post MI
C4049446: Neointimal hyperplasia
C0428474: Serum LDL cholesterol measurement
C2937421: Prostatic Hyperplasia
C1135196: Heart Failure, Diastolic
C2827469: Coronary Microvascular Disease
C1145628: Autonomic nervous system disorders
C0037889: Hereditary spherocytosis
C0005823: Blood Pressure
C3825201: Mitochondrial pathology
C0156149: Gastrointestinal tract vascular insufficiency
C2004435: Vascular insufficiency of intestine
C0242231: Coronary Stenosis
C1860707: TUBEROUS SCLEROSIS 2 (disorder)
C3544347: Intestinal fibrosis

c

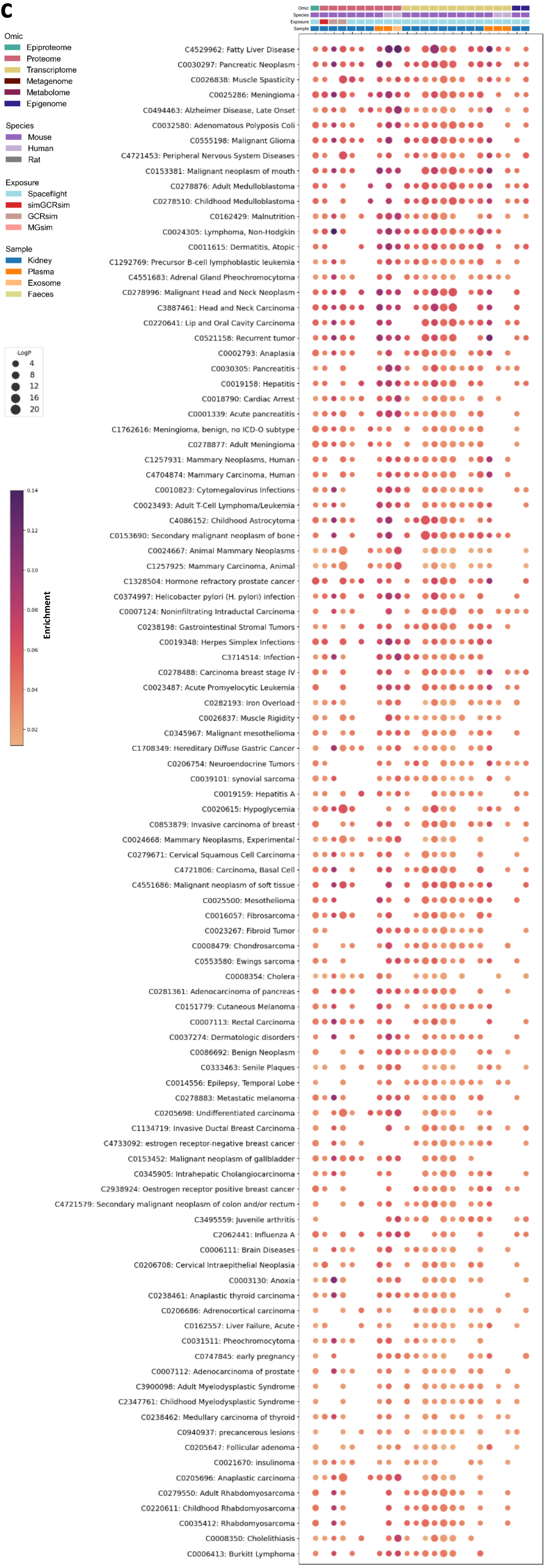
Omic
Species
Exposure
Sample
Omic
Epiproteome
Proteome
Transcriptome
Metagenome
Metabolome
Epigenome
Species
Mouse
Human
Rat
Exposure
Spaceflight
simGCRsim
GCRsim
MGsim
Sample
Kidney
Plasma
Exosome
Faeces
-LogP
4
8
12
16
20
Enrichment
0.14
0.12
0.10
0.08
0.06
0.04
0.02
C4529962: Fatty Liver Disease
C0030297: Pancreatic Neoplasm
C0026838: Muscle Spasticity
C0025286: Meningioma
C0494463: Alzheimer Disease, Late Onset
C0032580: Adenomatous Polyposis Coli
C0555198: Malignant Glioma
C4721453: Peripheral Nervous System Diseases
C0153381: Malignant neoplasm of mouth
C0278876: Adult Medulloblastoma
C0278510: Childhood Medulloblastoma
C0162429: Malnutrition
C0024305: Lymphoma, Non-Hodgkin
C0011615: Dermatitis, Atopic
C1292769: Precursor B-cell lymphoblastic leukemia
C4551683: Adrenal Gland Pheochromocytoma
C0278996: Malignant Head and Neck Neoplasm
C3887461: Head and Neck Carcinoma
C0220641: Lip and Oral Cavity Carcinoma
C0521158: Recurrent tumor
C0002793: Anaplasia
C0030305: Pancreatitis
C0019158: Hepatitis
C0018790: Cardiac Arrest
C0001339: Acute pancreatitis
C1762616: Meningioma, benign, no ICD-O subtype
C0278877: Adult Meningioma
C1257931: Mammary Neoplasms, Human
C4704874: Mammary Carcinoma, Human
C0010823: Cytomegalovirus Infections
C0023493: Adult T-Cell Lymphoma/Leukemia
C4086152: Childhood Astrocytoma
C0153690: Secondary malignant neoplasm of bone
C0024667: Animal Mammary Neoplasms
C1257925: Mammary Carcinoma, Animal
C1328504: Hormone refractory prostate cancer
C0374997: Helicobacter pylori (H. pylori) infection
C0007124: Noninfiltrating Intraductal Carcinoma
C0238198: Gastrointestinal Stromal Tumors
C0019348: Herpes Simplex Infections
C3714514: Infection
C0278488: Carcinoma breast stage IV
C0023487: Acute Promyelocytic Leukemia
C0282193: Iron Overload
C0026837: Muscle Rigidity
C0345967: Malignant mesothelioma
C1708349: Hereditary Diffuse Gastric Cancer
C0206754: Neuroendocrine Tumors
C0039101: synovial sarcoma
C0019159: Hepatitis A
C0020615: Hypoglycemia
C0853879: Invasive carcinoma of breast
C0024668: Mammary Neoplasms, Experimental
C0279671: Cervical Squamous Cell Carcinoma
C4721806: Carcinoma, Basal Cell
C4551686: Malignant neoplasm of soft tissue
C0025500: Mesothelioma
C0016057: Fibrosarcoma
C0023267: Fibroid Tumor
C0008479: Chondrosarcoma
C0553580: Ewings sarcoma
C0008354: Cholera
C0281361: Adenocarcinoma of pancreas
C0151779: Cutaneous Melanoma
C0007113: Rectal Carcinoma
C0037274: Dermatologic disorders
C0086692: Benign Neoplasm
C0333463: Senile Plaques
C0014556: Epilepsy, Temporal Lobe
C0278883: Metastatic melanoma
C0205698: Undifferentiated carcinoma
C1134719: Invasive Ductal Breast Carcinoma
C4733092: estrogen receptor-negative breast cancer
C0153452: Malignant neoplasm of gallbladder
C0345905: Intrahepatic Cholangiocarcinoma
C2938924: Oestrogen receptor positive breast cancer
C4721579: Secondary malignant neoplasm of colon and/or rectum
C3495559: Juvenile arthritis
C2062441: Influenza A
C0006111: Brain Diseases
C0206708: Cervical Intraepithelial Neoplasia
C0003130: Anoxia
C0238461: Anaplastic thyroid carcinoma
C0206686: Adrenocortical carcinoma
C0162557: Liver Failure, Acute
C0031511: Pheochromocytoma
C0747845: early pregnancy
C0007112: Adenocarcinoma of prostate
C3900098: Adult Myelodysplastic Syndrome
C2347761: Childhood Myelodysplastic Syndrome
C0238462: Medullary carcinoma of thyroid
C0940937: precancerous lesions
C0205647: Follicular adenoma
C0021670: insulinoma
C0205696: Anaplastic carcinoma
C0279550: Adult Rhabdomyosarcoma
C0220611: Childhood Rhabdomyosarcoma
C0035412: Rhabdomyosarcoma
C0008350: Cholelithiasis
C0006413: Burkitt Lymphoma

d

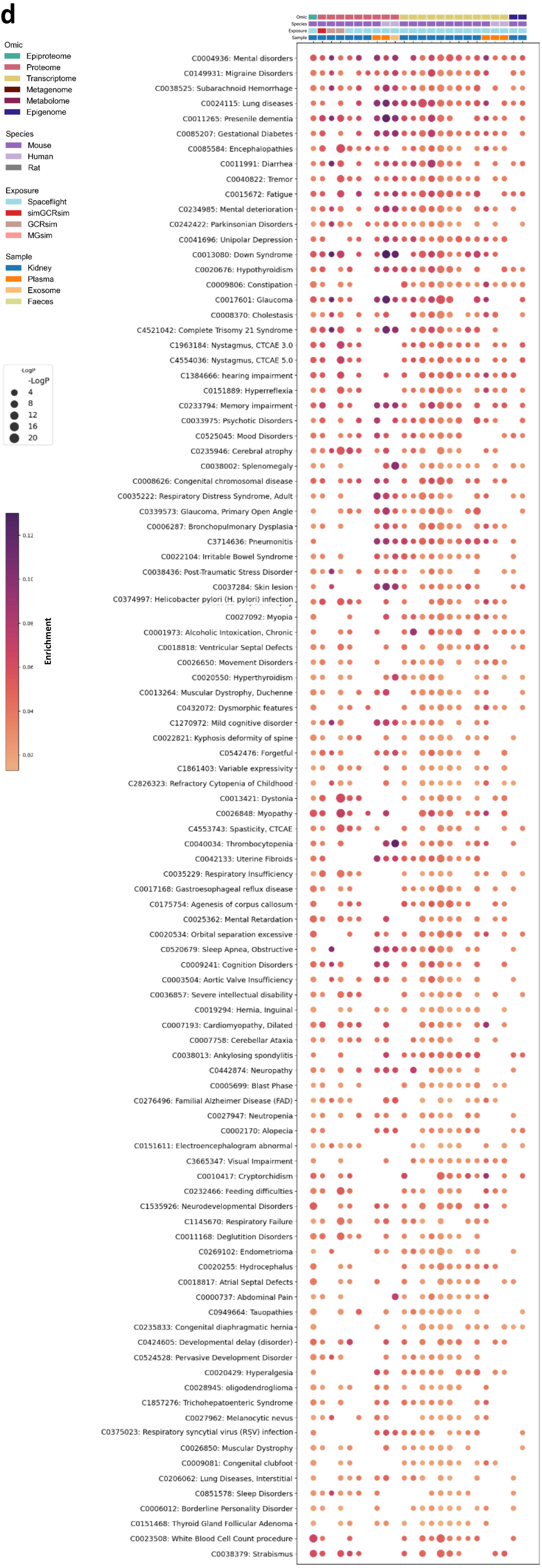
Omic
Species
Exposure
Sample
Omic
Epiproteome
Proteome
Transcriptome
Metagenome
Metabolome
Epigenome
Species
Mouse
Human
Rat
Exposure
Spaceflight
simGCRsim
GCRsim
MGsim
Sample
Kidney
Plasma
Exosome
Faeces
-LogP
4
8
12
16
20
Enrichment
0.12
0.10
0.08
0.06
0.04
0.02
C0004936: Mental disorders
C0149931: Migraine Disorders
C0038525: Subarachnoid Hemorrhage
C0024115: Lung diseases
C0011265: Presenile dementia
C0085207: Gestational Diabetes
C0085584: Encephalopathies
C0011991: Diarrhea
C0040822: Tremor
C0015672: Fatigue
C0234985: Mental deterioration
C0242422: Parkinsonian Disorders
C0041696: Unipolar Depression
C0013080: Down Syndrome
C0020676: Hypothyroidism
C0009806: Constipation
C0017601: Glaucoma
C0008370: Cholestasis
C4521042: Complete Trisomy 21 Syndrome
C1963184: Nystagmus, CTCAE 3.0
C4554036: Nystagmus, CTCAE 5.0
C1384666: hearing impairment
C0151889: Hyperreflexia
C0233794: Memory impairment
C0033975: Psychotic Disorders
C0525045: Mood Disorders
C0235946: Cerebral atrophy
C0038002: Splenomegaly
C0008626: Congenital chromosomal disease
C0035222: Respiratory Distress Syndrome, Adult
C0339573: Glaucoma, Primary Open Angle
C0006287: Bronchopulmonary Dysplasia
C3714636: Pneumonitis
C0022104: Irritable Bowel Syndrome
C0038436: Post-Traumatic Stress Disorder
C0037284: Skin lesion
C0374997: Helicobacter pylori (H. pylori) infection
C0027092: Myopia
C0001973: Alcoholic Intoxication, Chronic
C0018818: Ventricular Septal Defects
C0026650: Movement Disorders
C0020550: Hyperthyroidism
C0013264: Muscular Dystrophy, Duchenne
C0432072: Dysmorphic features
C1270972: Mild cognitive disorder
C0022821: Kyphosis deformity of spine
C0542476: Forgetful
C1861403: Variable expressivity
C2826323: Refractory Cytopenia of Childhood
C0013421: Dystonia
C0026848: Myopathy
C4553743: Spasticity, CTCAE
C0040034: Thrombocytopenia
C0042133: Uterine Fibroids
C0035229: Respiratory Insufficiency
C0017168: Gastroesophageal reflux disease
C0175754: Agenesis of corpus callosum
C0025362: Mental Retardation
C0020534: Orbital separation excessive
C0520679: Sleep Apnea, Obstructive
C0009241: Cognition Disorders
C0003504: Aortic Valve Insufficiency
C0036857: Severe intellectual disability
C0019294: Hernia, Inguinal
C0007193: Cardiomyopathy, Dilated
C0007758: Cerebellar Ataxia
C0038013: Ankylosing spondylitis
C0442874: Neuropathy
C0005699: Blast Phase
C0276496: Familial Alzheimer Disease (FAD)
C0027947: Neutropenia
C0002170: Alopecia
C0151611: Electroencephalogram abnormal
C3665347: Visual Impairment
C0010417: Cryptorchidism
C0232466: Feeding difficulties
C1535926: Neurodevelopmental Disorders
C1145670: Respiratory Failure
C0011168: Deglutition Disorders
C0269102: Endometrioma
C0020255: Hydrocephalus
C0018817: Atrial Septal Defects
C0000737: Abdominal Pain
C0949664: Tauopathies
C0235833: Congenital diaphragmatic hernia
C0424605: Developmental delay (disorder)
C0524528: Pervasive Development Disorder
C0020429: Hyperalgesia
C0028945: oligodendroglioma
C1857276: Trichohepatoenteric Syndrome
C0027962: Melanocytic nevus
C0375023: Respiratory syncytial virus (RSV) infection
C0026850: Muscular Dystrophy
C0009081: Congenital clubfoot
C0206062: Lung Diseases, Interstitial
C0851578: Sleep Disorders
C0006012: Borderline Personality Disorder
C0151468: Thyroid Gland Follicular Adenoma
C0023508: White Blood Cell Count procedure
C0038379: Strabismus

Supplementary Figure 3: Faecal Microbiome KEGG pathway over-representation analysis results

The top enriched KEGG microbiome pathway ontological terms. These were ranked and represented in descending order using the following rules: 1) No. of mission datasets it replicated in; 2) most significant p-value (no enrichment scores could be calculated). A Log10(P-value) of 1.3 was considered significant for ontological term enrichment and had to replicate in at least two datasets to be plotted. Plant-related terms removed from the display.

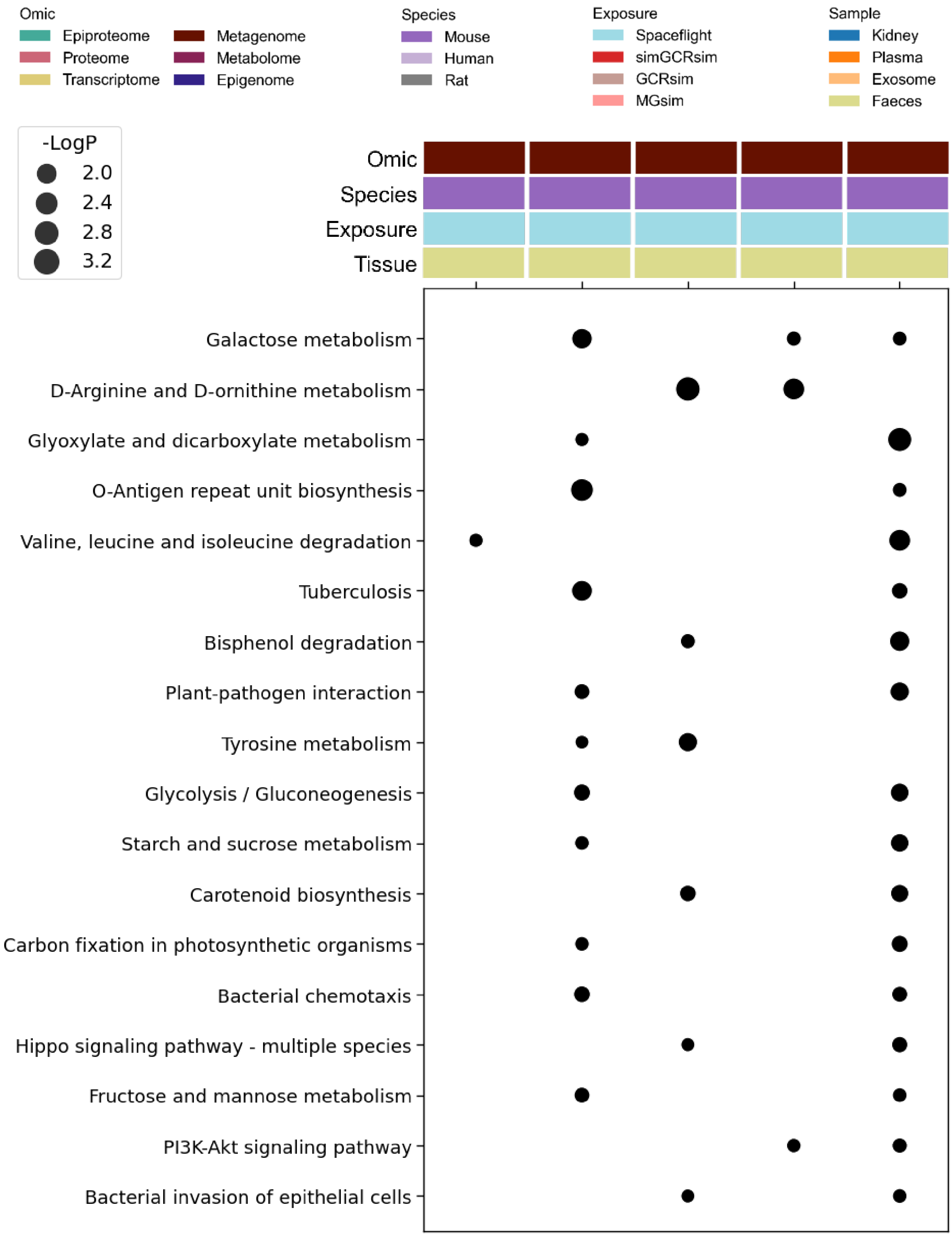

Omic
Epiproteome
Proteome
Transcriptome
Metagenome
Metabolome
Epigenome
Species
Mouse
Human
Rat
Exposure
Spaceflight
simGCRsim
GCRsim
MGsim
Sample
Kidney
Plasma
Exosome
Faeces
-LogP
2.0
2.4
2.8
3.2
Omic
Species
Exposure
Tissue
Galactose metabolism
D-Arginine and D-ornithine metabolism
Glyoxylate and dicarboxylate metabolism
O-Antigen repeat unit biosynthesis
Valine, leucine and isoleucine degradation
Tuberculosis
Bisphenol degradation
Plant-pathogen interaction
Tyrosine metabolism
Glycolysis / Gluconeogenesis
Starch and sucrose metabolism
Carotenoid biosynthesis
Carbon fixation in photosynthetic organisms
Bacterial chemotaxis
Hippo signaling pathway - multiple species
Fructose and mannose metabolism
PI3K-Akt signaling pathway
Bacterial invasion of epithelial cells

Supplementary Figure 4: Multi-omic KEGG pathway over-representation analysis top results by category grouping

The top 100 enriched KEGG pathway ontological terms relating to **a**, kidney health or cellular injury/stress, **b**, closely related to kidney injury and disease [vasculature; blood pressure; bone; muscle; diabetes] remodelling, **c**, no clear connection to the kidney, are presented. These were ranked and represented in descending order using the following rules: 1) No. of mission datasets it replicated in; 2) most significant p-value; 3) greatest enrichment. To integrate datasets from different omics modalities, species, missions and tissues, all biomolecules (e.g. phosphopeptides, proteins, transcripts and methylated DNA) were converted to the human orthologs where necessary and linked back to their HGNC gene symbol, aggregated and collapsed to single genes (e.g. multiple phosphosites, isoforms, CpG sites). A Log10(P-value) of 2 was considered significant for ontological term enrichment and had to replicate in at least two datasets to be plotted. Enrichment ratio; the number of differentially regulated hits in a dataset that belong to a given ontological term, normalised to the total number of statistically significant hits in the respective dataset.

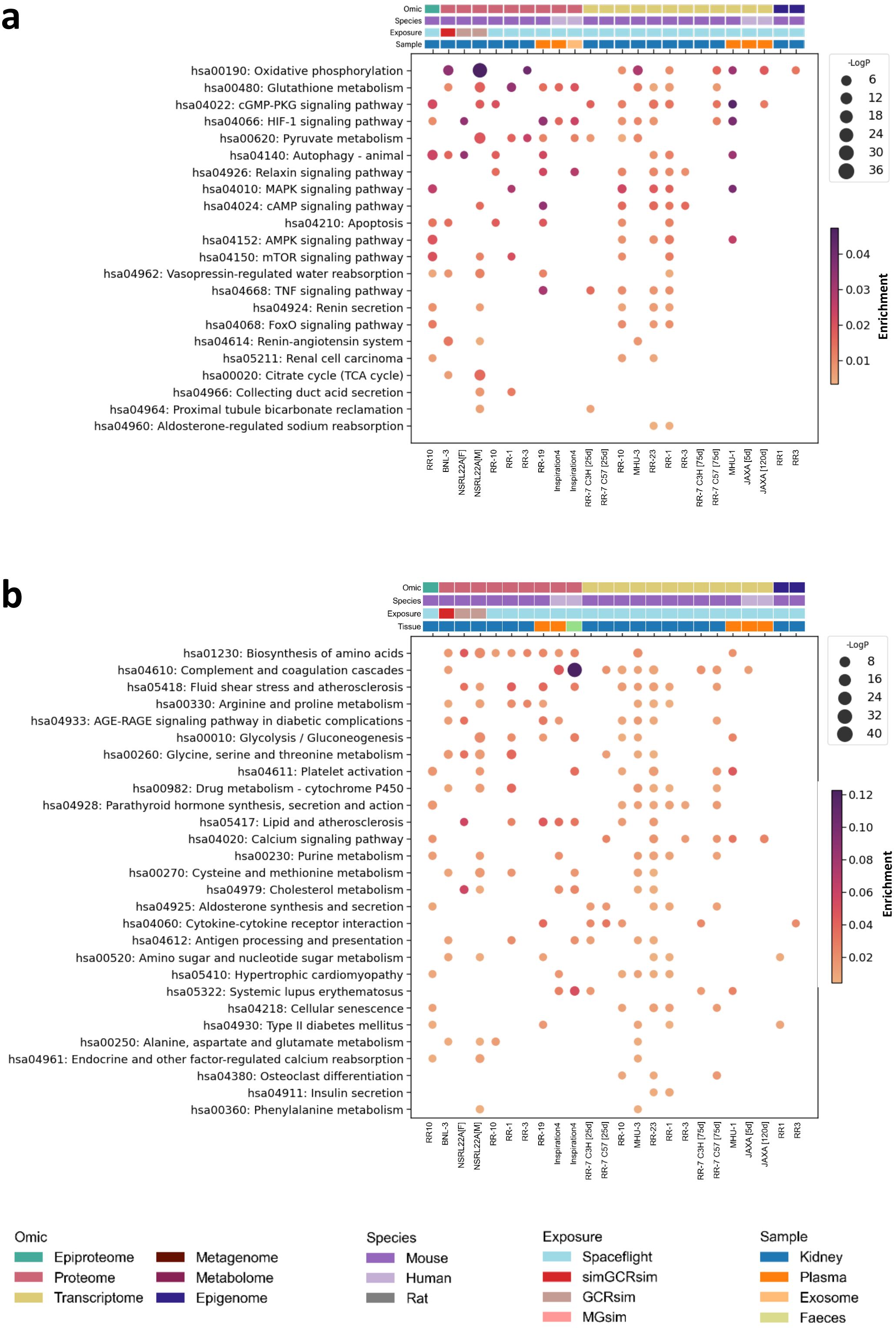

a
Omic
Species
Exposure
Sample
hsa00190: Oxidative phosphorylation
hsa00480: Glutathione metabolism
hsa04022: cGMP-PKG signaling pathway
hsa04066: HIF-1 signaling pathway
hsa00620: Pyruvate metabolism
hsa04140: Autophagy - animal
hsa04926: Relaxin signaling pathway
hsa04010: MAPK signaling pathway
hsa04024: cAMP signaling pathway
hsa04210: Apoptosis
hsa04152: AMPK signaling pathway
hsa04150: mTOR signaling pathway
hsa04962: Vasopressin-regulated water reabsorption
hsa04668: TNF signaling pathway
hsa04924: Renin secretion
hsa04068: FoxO signaling pathway
hsa04614: Renin-angiotensin system
hsa05211: Renal cell carcinoma
hsa00020: Citrate cycle (TCA cycle)
hsa04966: Collecting duct acid secretion
hsa04964: Proximal tubule bicarbonate reclamation
hsa04960: Aldosterone-regulated sodium reabsorption
RR10
BNL-3
NSRL22A[F]
NSRL22A[M]
RR-10
RR-1
RR-3
RR-19
Inspiration4
Inspiration4
RR-7 C3H [25d]
RR-7 C57 [25d]
RR-10
MHU-3
RR-23
RR-1
RR-3
RR-7 C3H [75d]
RR-7 C57 [75d]
MHU-1
JAXA [5d]
JAXA [120d]
RR1
RR3
-LogP
6
12
18
24
30
36
0.04
0.03
0.02
0.01
Enrichment
b
Omic
Species
Exposure
Tissue
hsa01230: Biosynthesis of amino acids
hsa04610: Complement and coagulation cascades
hsa05418: Fluid shear stress and atherosclerosis
hsa00330: Arginine and proline metabolism
hsa04933: AGE-RAGE signaling pathway in diabetic complications
hsa00010: Glycolysis / Gluconeogenesis
hsa00260: Glycine, serine and threonine metabolism
hsa04611: Platelet activation
hsa00982: Drug metabolism - cytochrome P450
hsa04928: Parathyroid hormone synthesis, secretion and action
hsa05417: Lipid and atherosclerosis
hsa04020: Calcium signaling pathway
hsa00230: Purine metabolism
hsa00270: Cysteine and methionine metabolism
hsa04979: Cholesterol metabolism
hsa04925: Aldosterone synthesis and secretion
hsa04060: Cytokine-cytokine receptor interaction
hsa04612: Antigen processing and presentation
hsa00520: Amino sugar and nucleotide sugar metabolism
hsa05410: Hypertrophic cardiomyopathy
hsa05322: Systemic lupus erythematosus
hsa04218: Cellular senescence
hsa04930: Type II diabetes mellitus
hsa00250: Alanine, aspartate and glutamate metabolism
hsa04961: Endocrine and other factor-regulated calcium reabsorption
hsa04380: Osteoclast differentiation
hsa04911: Insulin secretion
hsa00360: Phenylalanine metabolism
RR10
BNL-3
NSRL22A[F]
NSRL22A[M]
RR-10
RR-1
RR-3
RR-19
Inspiration4
Inspiration4
RR-7 C3H [25d]
RR-7 C57 [25d]
RR-10
MHU-3
RR-23
RR-1
RR-3
RR-7 C3H [75d]
RR-7 C57 [75d]
MHU-1
JAXA [5d]
JAXA [120d]
RR1
RR3
-LogP
8
16
24
32
40
0.12
0.10
0.08
0.06
0.04
0.02
Enrichment
Omic
Epiproteome
Proteome
Transcriptome
Metagenome
Metabolome
Epigenome
Species
Mouse
Human
Rat
Exposure
Spaceflight
simGCRsim
GCRsim
MGsim
Sample
Kidney
Plasma
Exosome
Faeces

c

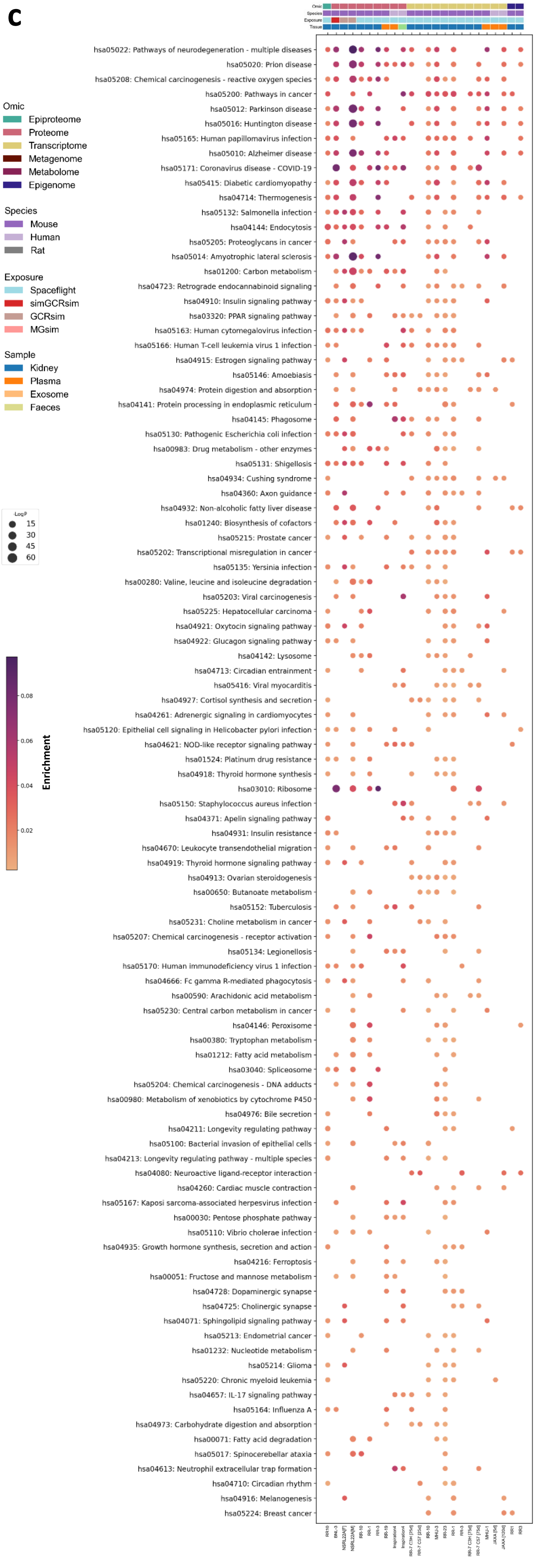
Omic
Species
Exposure
Tissue
Omic
Epiproteome
Proteome
Transcriptome
Metagenome
Metabolome
Epigenome
Species
Mouse
Human
Rat
Exposure
Spaceflight
simGCRsim
GCRsim
MGsim
Sample
Kidney
Plasma
Exosome
Faeces
-LogP
15
30
45
60
0.08
0.06
0.04
0.02
Enrichment
hsa05022: Pathways of neurodegeneration - multiple diseases
hsa05020: Prion disease
hsa05208: Chemical carcinogenesis - reactive oxygen species
hsa05200: Pathways in cancer
hsa05012: Parkinson disease
hsa05016: Huntington disease
hsa05165: Human papillomavirus infection
hsa05010: Alzheimer disease
hsa05171: Coronavirus disease - COVID-19
hsa05415: Diabetic cardiomyopathy
hsa04714: Thermogenesis
hsa05132: Salmonella infection
hsa04144: Endocytosis
hsa05205: Proteoglycans in cancer
hsa05014: Amyotrophic lateral sclerosis
hsa01200: Carbon metabolism
hsa04723: Retrograde endocannabinoid signaling
hsa04910: Insulin signaling pathway
hsa03320: PPAR signaling pathway
hsa05163: Human cytomegalovirus infection
hsa05166: Human T-cell leukemia virus 1 infection
hsa04915: Estrogen signaling pathway
hsa05146: Amoebiasis
hsa04974: Protein digestion and absorption
hsa04141: Protein processing in endoplasmic reticulum
hsa04145: Phagosome
hsa05130: Pathogenic Escherichia coli infection
hsa00983: Drug metabolism - other enzymes
hsa05131: Shigellosis
hsa04934: Cushing syndrome
hsa04360: Axon guidance
hsa04932: Non-alcoholic fatty liver disease
hsa01240: Biosynthesis of cofactors
hsa05215: Prostate cancer
hsa05202: Transcriptional misregulation in cancer
hsa05135: Yersinia infection
hsa00280: Valine, leucine and isoleucine degradation
hsa05203: Viral carcinogenesis
hsa05225: Hepatocellular carcinoma
hsa04921: Oxytocin signaling pathway
hsa04922: Glucagon signaling pathway
hsa04142: Lysosome
hsa04713: Circadian entrainment
hsa05416: Viral myocarditis
hsa04927: Cortisol synthesis and secretion
hsa04261: Adrenergic signaling in cardiomyocytes
hsa05120: Epithelial cell signaling in Helicobacter pylori infection
hsa04621: NOD-like receptor signaling pathway
hsa01524: Platinum drug resistance
hsa04918: Thyroid hormone synthesis
hsa03010: Ribosome
hsa05150: Staphylococcus aureus infection
hsa04371: Apelin signaling pathway
hsa04931: Insulin resistance
hsa04670: Leukocyte transendothelial migration
hsa04919: Thyroid hormone signaling pathway
hsa04913: Ovarian steroidogenesis
hsa00650: Butanoate metabolism
hsa05152: Tuberculosis
hsa05231: Choline metabolism in cancer
hsa05207: Chemical carcinogenesis - receptor activation
hsa05134: Legionellosis
hsa05170: Human immunodeficiency virus 1 infection
hsa04666: Fc gamma R-mediated phagocytosis
hsa00590: Arachidonic acid metabolism
hsa05230: Central carbon metabolism in cancer
hsa04146: Peroxisome
hsa00380: Tryptophan metabolism
hsa01212: Fatty acid metabolism
hsa03040: Spliceosome
hsa05204: Chemical carcinogenesis - DNA adducts
hsa00980: Metabolism of xenobiotics by cytochrome P450
hsa04976: Bile secretion
hsa04211: Longevity regulating pathway
hsa05100: Bacterial invasion of epithelial cells
hsa04213: Longevity regulating pathway - multiple species
hsa04080: Neuroactive ligand-receptor interaction
hsa04260: Cardiac muscle contraction
hsa05167: Kaposi sarcoma-associated herpesvirus infection
hsa00030: Pentose phosphate pathway
hsa05110: Vibrio cholerae infection
hsa04935: Growth hormone synthesis, secretion and action
hsa04216: Ferroptosis
hsa00051: Fructose and mannose metabolism
hsa04728: Dopaminergic synapse
hsa04725: Cholinergic synapse
hsa04071: Sphingolipid signaling pathway
hsa05213: Endometrial cancer
hsa01232: Nucleotide metabolism
hsa05214: Glioma
hsa05220: Chronic myeloid leukemia
hsa04657: IL-17 signaling pathway
hsa05164: Influenza A
hsa04973: Carbohydrate digestion and absorption
hsa00071: Fatty acid degradation
hsa05017: Spinocerebellar ataxia
hsa04613: Neutrophil extracellular trap formation
hsa04710: Circadian rhythm
hsa04916: Melanogenesis
hsa05224: Breast cancer
RR10
BNL-3
NSRL22A[F]
NSRL22A[M]
RR-10
RR-1
RR-3
RR-19
Inspiration4
Inspiration4
RR-7 C3H [25d]
RR-7 C57 [25d]
RR-10
MHU-3
RR-23
RR-1
RR-3
RR-7 C3H [75d]
RR-7 C57 [75d]
MHU-1
JAXA [5d]
JAXA [120d]
RR1
RR3

Supplementary Figure 5: Multi-omic Gene Ontology (GO) over-representation analysis top results by category grouping

The top 100 enriched GO ontological terms relating to **a**, biological process, **b**, cellular component **c**, molecular function are presented. These were ranked and represented in descending order using the following rules: 1) No. of mission datasets it replicated in; 2) most significant p-value; 3) greatest enrichment. To integrate datasets from different omics modalities, species, missions and tissues, all biomolecules (e.g. phosphopeptides, proteins, transcripts and methylated DNA) were converted to the human orthologs where necessary and linked back to their HGNC gene symbol, aggregated and collapsed to single genes (e.g. multiple phosphosites, isoforms, CpG sites). A Log10(P-value) of 2 was considered significant for ontological term enrichment and had to replicate in at least two datasets to be plotted. Enrichment ratio; the number of differentially regulated hits in a dataset that belong to a given ontological term, normalised to the total number of statistically significant hits in the respective dataset.

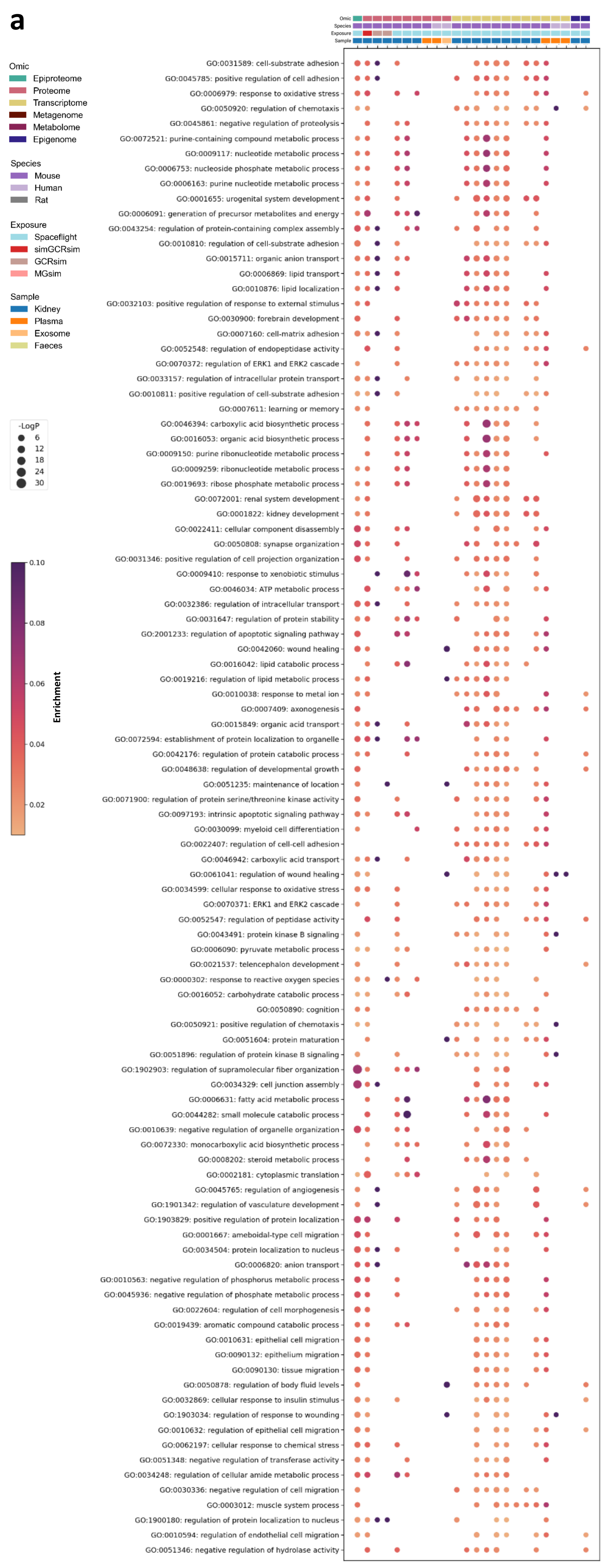
a
Omic
Species
Exposure
Sample
Omic
Epiproteome
Proteome
Transcriptome
Metagenome
Metabolome
Epigenome
Species
Mouse
Human
Rat
Exposure
Spaceflight
simGCRsim
GCRsim
MGsim
Sample
Kidney
Plasma
Exosome
Faeces
-LogP
6
12
18
24
30
Enrichment
0.10
0.08
0.06
0.04
0.02
GO:0031589: cell-substrate adhesion
GO:0045785: positive regulation of cell adhesion
GO:0006979: response to oxidative stress
GO:0050920: regulation of chemotaxis
GO:0045861: negative regulation of proteolysis
GO:0072521: purine-containing compound metabolic process
GO:0009117: nucleotide metabolic process
GO:0006753: nucleoside phosphate metabolic process
GO:0006163: purine nucleotide metabolic process
GO:0001655: urogenital system development
GO:0006091: generation of precursor metabolites and energy
GO:0043254: regulation of protein-containing complex assembly
GO:0010810: regulation of cell-substrate adhesion
GO:0015711: organic anion transport
GO:0006869: lipid transport
GO:0010876: lipid localization
GO:0032103: positive regulation of response to external stimulus
GO:0030900: forebrain development
GO:0007160: cell-matrix adhesion
GO:0052548: regulation of endopeptidase activity
GO:0070372: regulation of ERK1 and ERK2 cascade
GO:0033157: regulation of intracellular protein transport
GO:0010811: positive regulation of cell-substrate adhesion
GO:0007611: learning or memory
GO:0046394: carboxylic acid biosynthetic process
GO:0016053: organic acid biosynthetic process
GO:0009150: purine ribonucleotide metabolic process
GO:0009259: ribonucleotide metabolic process
GO:0019693: ribose phosphate metabolic process
GO:0072001: renal system development
GO:0001822: kidney development
GO:0022411: cellular component disassembly
GO:0050808: synapse organization
GO:0031346: positive regulation of cell projection organization
GO:0009410: response to xenobiotic stimulus
GO:0046034: ATP metabolic process
GO:0032386: regulation of intracellular transport
GO:0031647: regulation of protein stability
GO:2001233: regulation of apoptotic signaling pathway
GO:0042060: wound healing
GO:0016042: lipid catabolic process
GO:0019216: regulation of lipid metabolic process
GO:0010038: response to metal ion
GO:0007409: axonogenesis
GO:0015849: organic acid transport
GO:0072594: establishment of protein localization to organelle
GO:0042176: regulation of protein catabolic process
GO:0048638: regulation of developmental growth
GO:0051235: maintenance of location
GO:0071900: regulation of protein serine/threonine kinase activity
GO:0097193: intrinsic apoptotic signaling pathway
GO:0030099: myeloid cell differentiation
GO:0022407: regulation of cell-cell adhesion
GO:0046942: carboxylic acid transport
GO:0061041: regulation of wound healing
GO:0034599: cellular response to oxidative stress
GO:0070371: ERK1 and ERK2 cascade
GO:0052547: regulation of peptidase activity
GO:0043491: protein kinase B signaling
GO:0006090: pyruvate metabolic process
GO:0021537: telencephalon development
GO:0000302: response to reactive oxygen species
GO:0016052: carbohydrate catabolic process
GO:0050890: cognition
GO:0050921: positive regulation of chemotaxis
GO:0051604: protein maturation
GO:0051896: regulation of protein kinase B signaling
GO:1902903: regulation of supramolecular fiber organization
GO:0034329: cell junction assembly
GO:0006631: fatty acid metabolic process
GO:0044282: small molecule catabolic process
GO:0010639: negative regulation of organelle organization
GO:0072330: monocarboxylic acid biosynthetic process
GO:0008202: steroid metabolic process
GO:0002181: cytoplasmic translation
GO:0045765: regulation of angiogenesis
GO:1901342: regulation of vasculature development
GO:1903829: positive regulation of protein localization
GO:0001667: ameboidal-type cell migration
GO:0034504: protein localization to nucleus
GO:0006820: anion transport
GO:0010563: negative regulation of phosphorus metabolic process
GO:0045936: negative regulation of phosphate metabolic process
GO:0022604: regulation of cell morphogenesis
GO:0019439: aromatic compound catabolic process
GO:0010631: epithelial cell migration
GO:0090132: epithelium migration
GO:0090130: tissue migration
GO:0050878: regulation of body fluid levels
GO:0032869: cellular response to insulin stimulus
GO:1903034: regulation of response to wounding
GO:0010632: regulation of epithelial cell migration
GO:0062197: cellular response to chemical stress
GO:0051348: negative regulation of transferase activity
GO:0034248: regulation of cellular amide metabolic process
GO:0030336: negative regulation of cell migration
GO:0003012: muscle system process
GO:1900180: regulation of protein localization to nucleus
GO:0010594: regulation of endothelial cell migration
GO:0051346: negative regulation of hydrolase activity

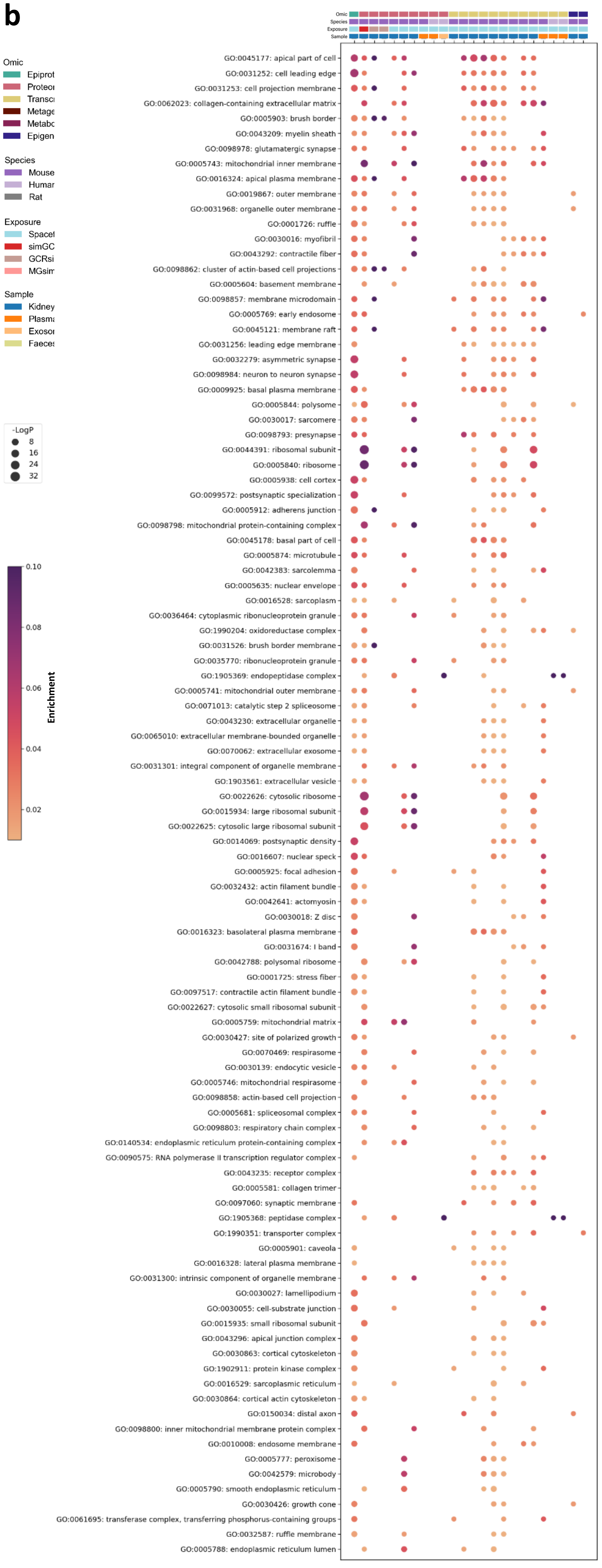
b
Omic
Species
Exposure
Sample
Omic
Epiprot
Proteo
Transc
Metage
Metabo
Epigen
Species
Mouse
Human
Rat
Exposure
Spacel
simGC
GCRsi
MGsim
Sample
Kidney
Plasma
Exosor
Faeces
-LogP
8
16
24
32
Enrichment
0.10
0.08
0.06
0.04
0.02
GO:0045177: apical part of cell
GO:0031252: cell leading edge
GO:0031253: cell projection membrane
GO:0062023: collagen-containing extracellular matrix
GO:0005903: brush border
GO:0043209: myelin sheath
GO:0098978: glutamatergic synapse
GO:0005743: mitochondrial inner membrane
GO:0016324: apical plasma membrane
GO:0019867: outer membrane
GO:0031968: organelle outer membrane
GO:0001726: ruffle
GO:0030016: myofibril
GO:0043292: contractile fiber
GO:0098862: cluster of actin-based cell projections
GO:0005604: basement membrane
GO:0098857: membrane microdomain
GO:0005769: early endosome
GO:0045121: membrane raft
GO:0031256: leading edge membrane
GO:0032279: asymmetric synapse
GO:0098984: neuron to neuron synapse
GO:0009925: basal plasma membrane
GO:0005844: polysome
GO:0030017: sarcomere
GO:0098793: presynapse
GO:0044391: ribosomal subunit
GO:0005840: ribosome
GO:0005938: cell cortex
GO:0099572: postsynaptic specialization
GO:0005912: adherens junction
GO:0098798: mitochondrial protein-containing complex
GO:0045178: basal part of cell
GO:0005874: microtubule
GO:0042383: sarcolemma
GO:0005635: nuclear envelope
GO:0016528: sarcoplasm
GO:0036464: cytoplasmic ribonucleoprotein granule
GO:1990204: oxidoreductase complex
GO:0031526: brush border membrane
GO:0035770: ribonucleoprotein granule
GO:1905369: endopeptidase complex
GO:0005741: mitochondrial outer membrane
GO:0071013: catalytic step 2 spliceosome
GO:0043230: extracellular organelle
GO:0065010: extracellular membrane-bounded organelle
GO:0070062: extracellular exosome
GO:0031301: integral component of organelle membrane
GO:1903561: extracellular vesicle
GO:0022626: cytosolic ribosome
GO:0015934: large ribosomal subunit
GO:0022625: cytosolic large ribosomal subunit
GO:0014069: postsynaptic density
GO:0016607: nuclear speck
GO:0005925: focal adhesion
GO:0032432: actin filament bundle
GO:0042641: actomyosin
GO:0030018: Z disc
GO:0016323: basolateral plasma membrane
GO:0031674: I band
GO:0042788: polysomal ribosome
GO:0001725: stress fiber
GO:0097517: contractile actin filament bundle
GO:0022627: cytosolic small ribosomal subunit
GO:0005759: mitochondrial matrix
GO:0030427: site of polarized growth
GO:0070469: respirasome
GO:0030139: endocytic vesicle
GO:0005746: mitochondrial respirasome
GO:0098858: actin-based cell projection
GO:0005681: spliceosomal complex
GO:0098803: respiratory chain complex
GO:0140534: endoplasmic reticulum protein-containing complex
GO:0090575: RNA polymerase II transcription regulator complex
GO:0043235: receptor complex
GO:0005581: collagen trimer
GO:0097060: synaptic membrane
GO:1905368: peptidase complex
GO:1990351: transporter complex
GO:0005901: caveola
GO:0016328: lateral plasma membrane
GO:0031300: intrinsic component of organelle membrane
GO:0030027: lamellipodium
GO:0030055: cell-substrate junction
GO:0015935: small ribosomal subunit
GO:0043296: apical junction complex
GO:0030863: cortical cytoskeleton
GO:1902911: protein kinase complex
GO:0016529: sarcoplasmic reticulum
GO:0030864: cortical actin cytoskeleton
GO:0150034: distal axon
GO:0098800: inner mitochondrial membrane protein complex
GO:0010008: endosome membrane
GO:0005777: peroxisome
GO:0042579: microbody
GO:0005790: smooth endoplasmic reticulum
GO:0030426: growth cone
GO:0061695: transferase complex, transferring phosphorus-containing groups
GO:0032587: ruffle membrane
GO:0005788: endoplasmic reticulum lumen

c

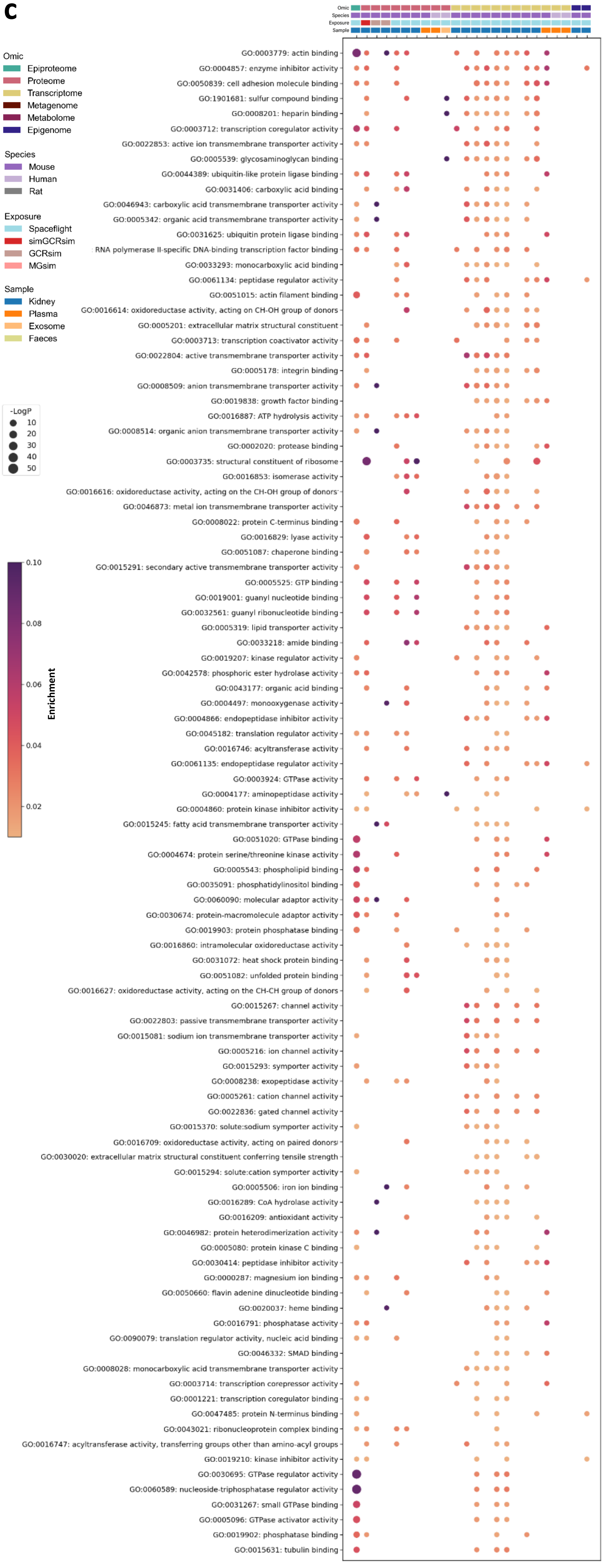
Omic
Species
Exposure
Sample
Omic
Epiproteome
Proteome
Transcriptome
Metagenome
Metabolome
Epigenome
Species
Mouse
Human
Rat
Exposure
Spaceflight
simGCRsim
GCRsim
MGsim
Sample
Kidney
Plasma
Exosome
Faeces
-LogP
10
20
30
40
50
Enrichment
0.10
0.08
0.06
0.04
0.02
GO:0003779: actin binding
GO:0004857: enzyme inhibitor activity
GO:0050839: cell adhesion molecule binding
GO:1901681: sulfur compound binding
GO:0008201: heparin binding
GO:0003712: transcription coregulator activity
GO:0022853: active ion transmembrane transporter activity
GO:0005539: glycosaminoglycan binding
GO:0044389: ubiquitin-like protein ligase binding
GO:0031406: carboxylic acid binding
GO:0046943: carboxylic acid transmembrane transporter activity
GO:0005342: organic acid transmembrane transporter activity
GO:0031625: ubiquitin protein ligase binding
RNA polymerase II-specific DNA-binding transcription factor binding
GO:0033293: monocarboxylic acid binding
GO:0061134: peptidase regulator activity
GO:0051015: actin filament binding
GO:0016614: oxidoreductase activity, acting on CH-OH group of donors
GO:0005201: extracellular matrix structural constituent
GO:0003713: transcription coactivator activity
GO:0022804: active transmembrane transporter activity
GO:0005178: integrin binding
GO:0008509: anion transmembrane transporter activity
GO:0019838: growth factor binding
GO:0016887: ATP hydrolysis activity
GO:0008514: organic anion transmembrane transporter activity
GO:0002020: protease binding
GO:0003735: structural constituent of ribosome
GO:0016853: isomerase activity
GO:0016616: oxidoreductase activity, acting on the CH-OH group of donors
GO:0046873: metal ion transmembrane transporter activity
GO:0008022: protein C-terminus binding
GO:0016829: lyase activity
GO:0051087: chaperone binding
GO:0015291: secondary active transmembrane transporter activity
GO:0005525: GTP binding
GO:0019001: guanyl nucleotide binding
GO:0032561: guanyl ribonucleotide binding
GO:0005319: lipid transporter activity
GO:0033218: amide binding
GO:0019207: kinase regulator activity
GO:0042578: phosphoric ester hydrolase activity
GO:0043177: organic acid binding
GO:0004497: monooxygenase activity
GO:0004866: endopeptidase inhibitor activity
GO:0045182: translation regulator activity
GO:0016746: acyltransferase activity
GO:0061135: endopeptidase regulator activity
GO:0003924: GTPase activity
GO:0004177: aminopeptidase activity
GO:0004860: protein kinase inhibitor activity
GO:0015245: fatty acid transmembrane transporter activity
GO:0051020: GTPase binding
GO:0004674: protein serine/threonine kinase activity
GO:0005543: phospholipid binding
GO:0035091: phosphatidylinositol binding
GO:0060090: molecular adaptor activity
GO:0030674: protein-macromolecule adaptor activity
GO:0019903: protein phosphatase binding
GO:0016860: intramolecular oxidoreductase activity
GO:0031072: heat shock protein binding
GO:0051082: unfolded protein binding
GO:0016627: oxidoreductase activity, acting on the CH-CH group of donors
GO:0015267: channel activity
GO:0022803: passive transmembrane transporter activity
GO:0015081: sodium ion transmembrane transporter activity
GO:0005216: ion channel activity
GO:0015293: symporter activity
GO:0008238: exopeptidase activity
GO:0005261: cation channel activity
GO:0022836: gated channel activity
GO:0015370: solute:sodium symporter activity
GO:0016709: oxidoreductase activity, acting on paired donors
GO:0030020: extracellular matrix structural constituent conferring tensile strength
GO:0015294: solute:cation symporter activity
GO:0005506: iron ion binding
GO:0016289: CoA hydrolase activity
GO:0016209: antioxidant activity
GO:0046982: protein heterodimerization activity
GO:0005080: protein kinase C binding
GO:0030414: peptidase inhibitor activity
GO:0000287: magnesium ion binding
GO:0050660: flavin adenine dinucleotide binding
GO:0020037: heme binding
GO:0016791: phosphatase activity
GO:0090079: translation regulator activity, nucleic acid binding
GO:0046332: SMAD binding
GO:0008028: monocarboxylic acid transmembrane transporter activity
GO:0003714: transcription corepressor activity
GO:0001221: transcription coregulator binding
GO:0047485: protein N-terminus binding
GO:0043021: ribonucleoprotein complex binding
GO:0016747: acyltransferase activity, transferring groups other than amino-acyl groups
GO:0019210: kinase inhibitor activity
GO:0030695: GTPase regulator activity
GO:0060589: nucleoside-triphosphatase regulator activity
GO:0031267: small GTPase binding
GO:0005096: GTPase activator activity
GO:0019902: phosphatase binding
GO:0015631: tubulin binding

Supplementary Figure 6: qPCR of mitochondrial fission and fusion markers

a, Heatmap of mitochondrial fission and fusion mRNA transcript markers from RR-10 spaceflight-exposed mice (28 days) kidney tissue. **b**, Relative change in the geometric means of all fission or fusion markers. Data are mean ± 95% CI. A P-value of <0.05 was considered significant. **c**, Stability ranking of reference genes. **d**, Individual normalisation factors by animal. **e**, Mean relative difference in normalisation factor for groups. Data are mean ± range. A P-value of <0.05 was considered significant. **f,** RNA input vs relative reference gene levels.

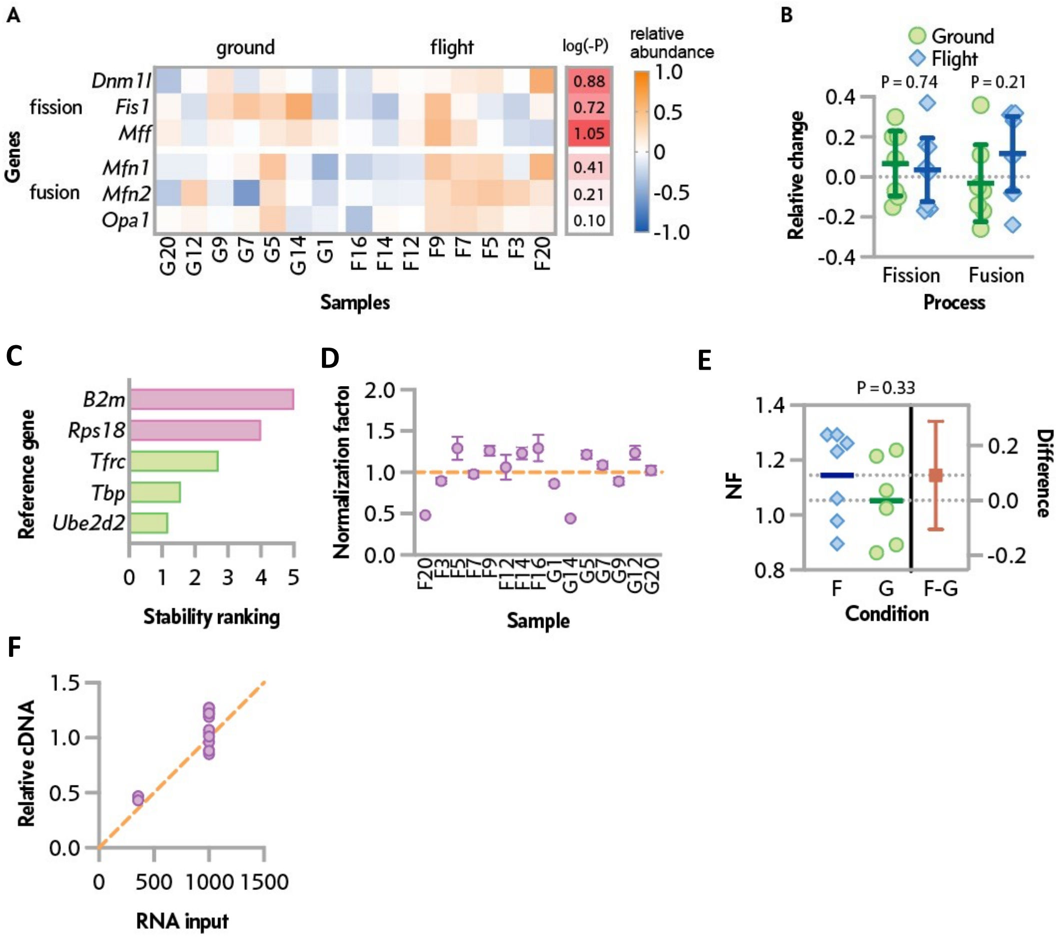
A
ground
flight
log(-P)
relative abundance
1.0
0.5
0
-0.5
-1.0
Dnm1l
fission
Fis1
Mff
Genes
Mfn1
fusion
Mfn2
Opa1
0.88
0.72
1.05
0.41
0.21
0.10
G20 G12 G9 G7 G5 G14 G1 F16 F14 F12 F9 F7 F5 F3 F20
Samples
B
Ground
Flight
P = 0.74
P = 0.21
Relative change
0.4
0.2
0.0
-0.2
-0.4
Fission
Fusion
Process
C
Reference gene
B2m
Rps18
Tfrc
Tbp
Ube2d2
0 1 2 3 4 5
Stability ranking
D
Normalization factor
2.0
1.5
1.0
0.5
0.0
F20 F3 F5 F7 F9 F12 F14 F16 G1 G14 G5 G7 G9 G12 G20
Sample
E
P = 0.33
NF
1.4
1.2
1.0
0.8
Difference
0.2
0.0
-0.2
F
G
F-G
Condition
F
Relative cDNA
1.5
1.0
0.5
0.0
0
500
1000
1500
RNA input

Supplementary Figure 7: SPOKE Integrated knowledge network analysis

SPOKE (Scalable Precision Medicine Open Knowledge Environment) was used to integrate the heterogeneous data from several missions, experimental conditions, tissues, species and assay modalities. **a,** SPOKE workflow from inputs through to disease and anatomical associations, via convergent entry nodes for protein, gene, compound and organism nodes. **b,** Meta pathways to kidney disease (red nodes on the far right). Note the abundance of nodes associated with all of the inputting nodes, implying orthogonal confirmation across the inputted 18 -omics datasets of spaceflight conditions and renal pathology.

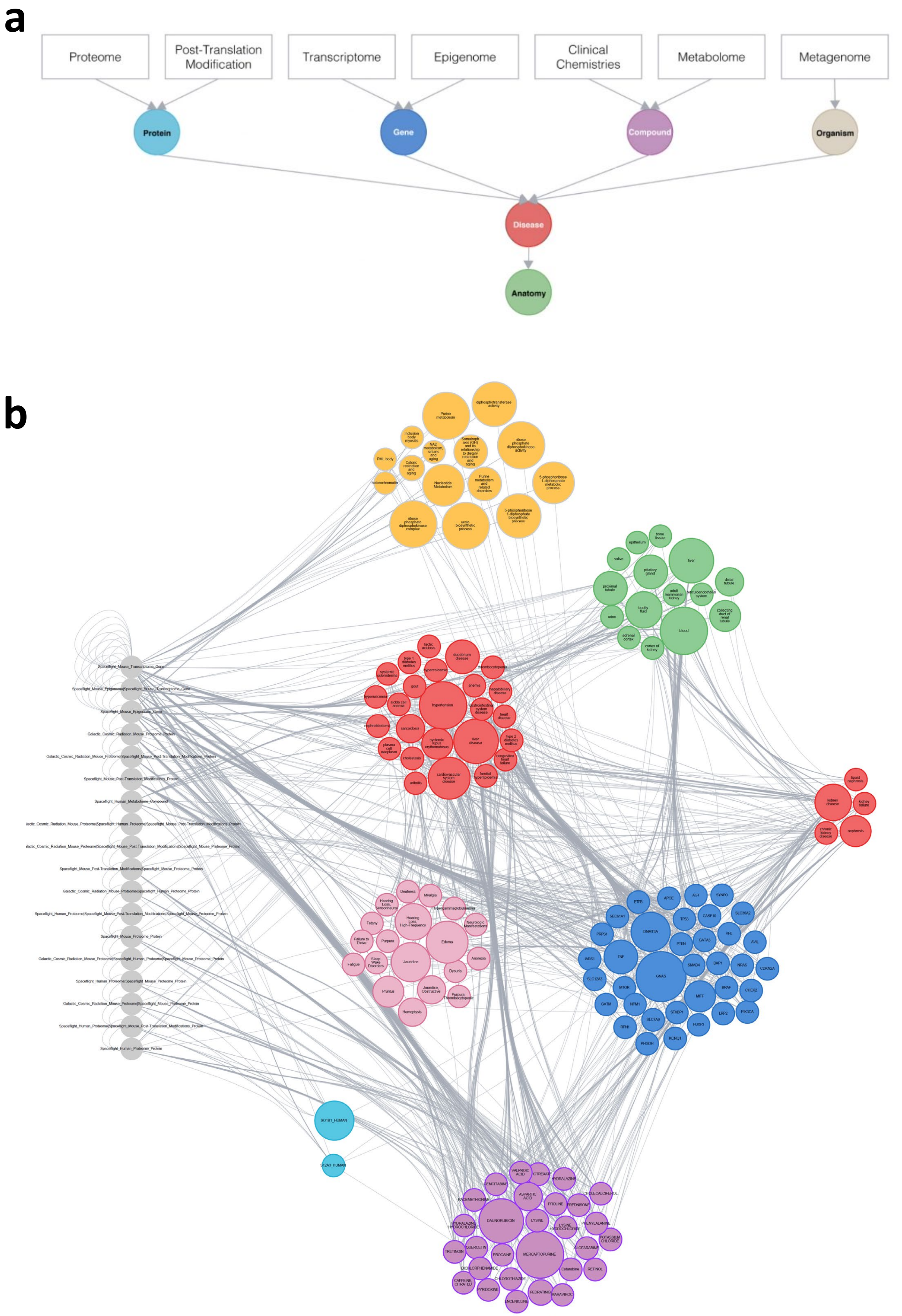
a
Proteome
Post-Translation Modification
Transcriptome
Epigenome
Clinical Chemistries
Metabolome
Metagenome
Protein
Gene
Compound
Organism
Disease
Anatomy
b

Supplementary Figure 8: Plasma miRNA Weighted Correlation Network Analysis (WGCNA)

Plasma miRNAs from BNL-1 simGCRsim-exposed mice (~1.5-year dose equivalent) for WGCNA. **a,** Dendrogram grouping miRNAs into three module-eigengenes (ME) according to co-expression profiles. **b,** Comparisons of ME's expression patterns in Sham vs GCR animals. Abundance heatmaps for constituent miRNAs in the ME are given in **c,** ME0, **d,** ME1, **e,** ME2.

a

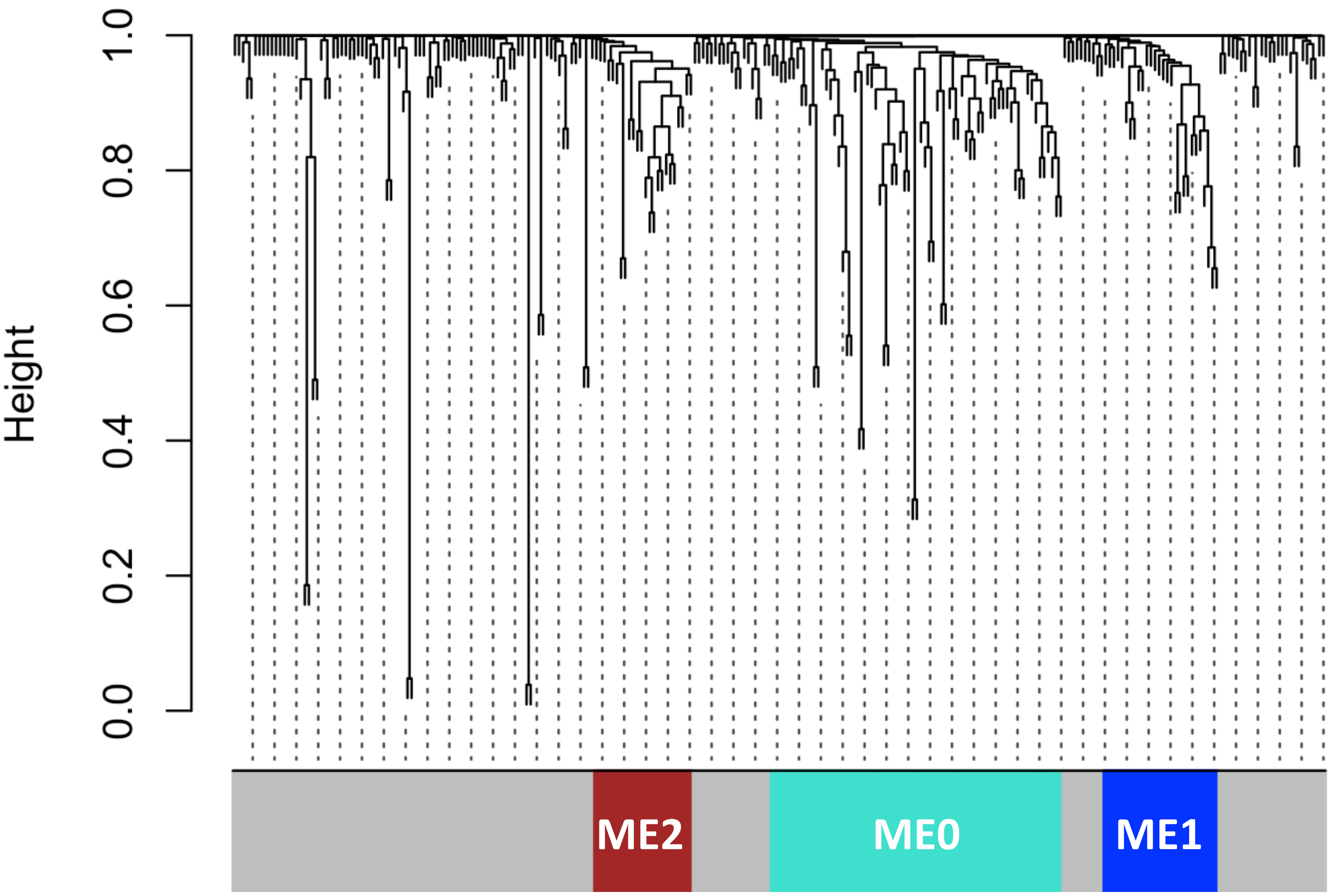

Height
1.0
0.8
0.6
0.4
0.2
0.0
ME2
ME0
ME1

b

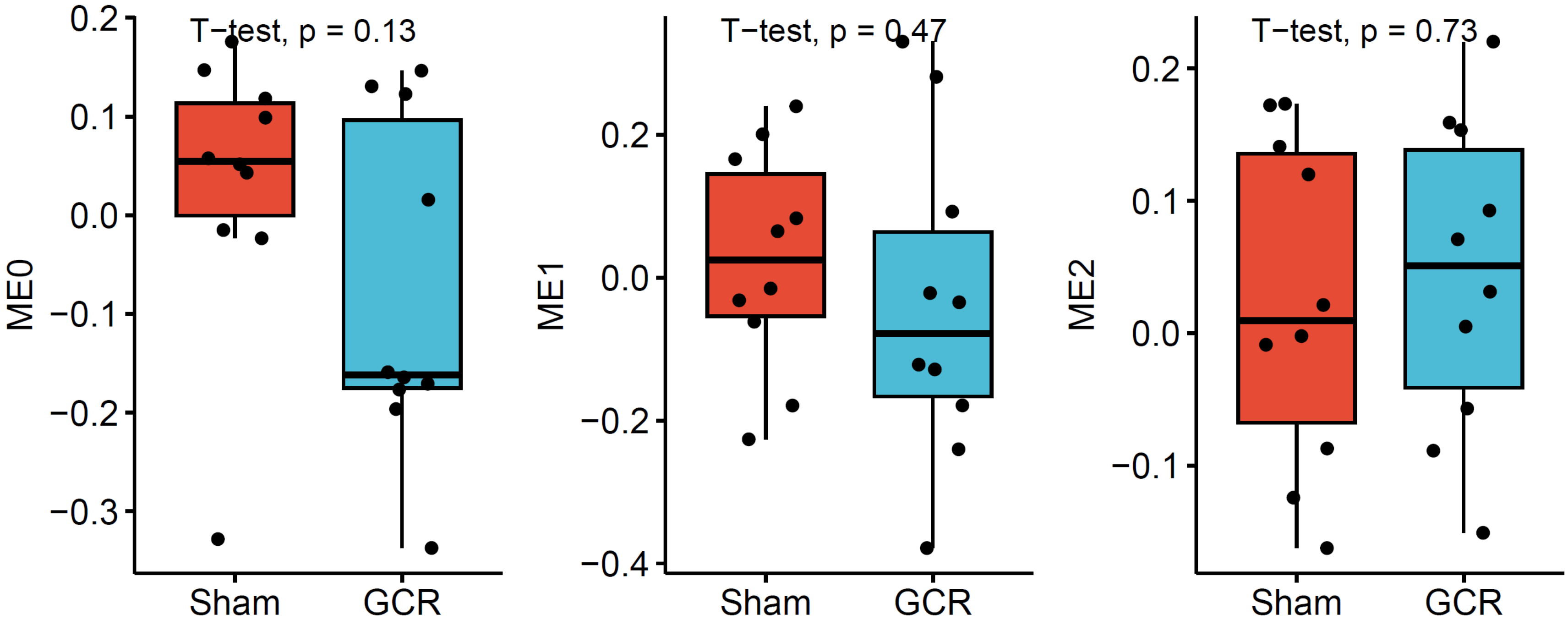

T−test, p = 0.13
ME0
0.2
0.1
0.0
−0.1
−0.2
−0.3
Sham
GCR
T−test, p = 0.47
ME1
0.2
0.0
−0.2
−0.4
Sham
GCR
T−test, p = 0.73
ME2
0.2
0.1
0.0
−0.1
Sham
GCR

c

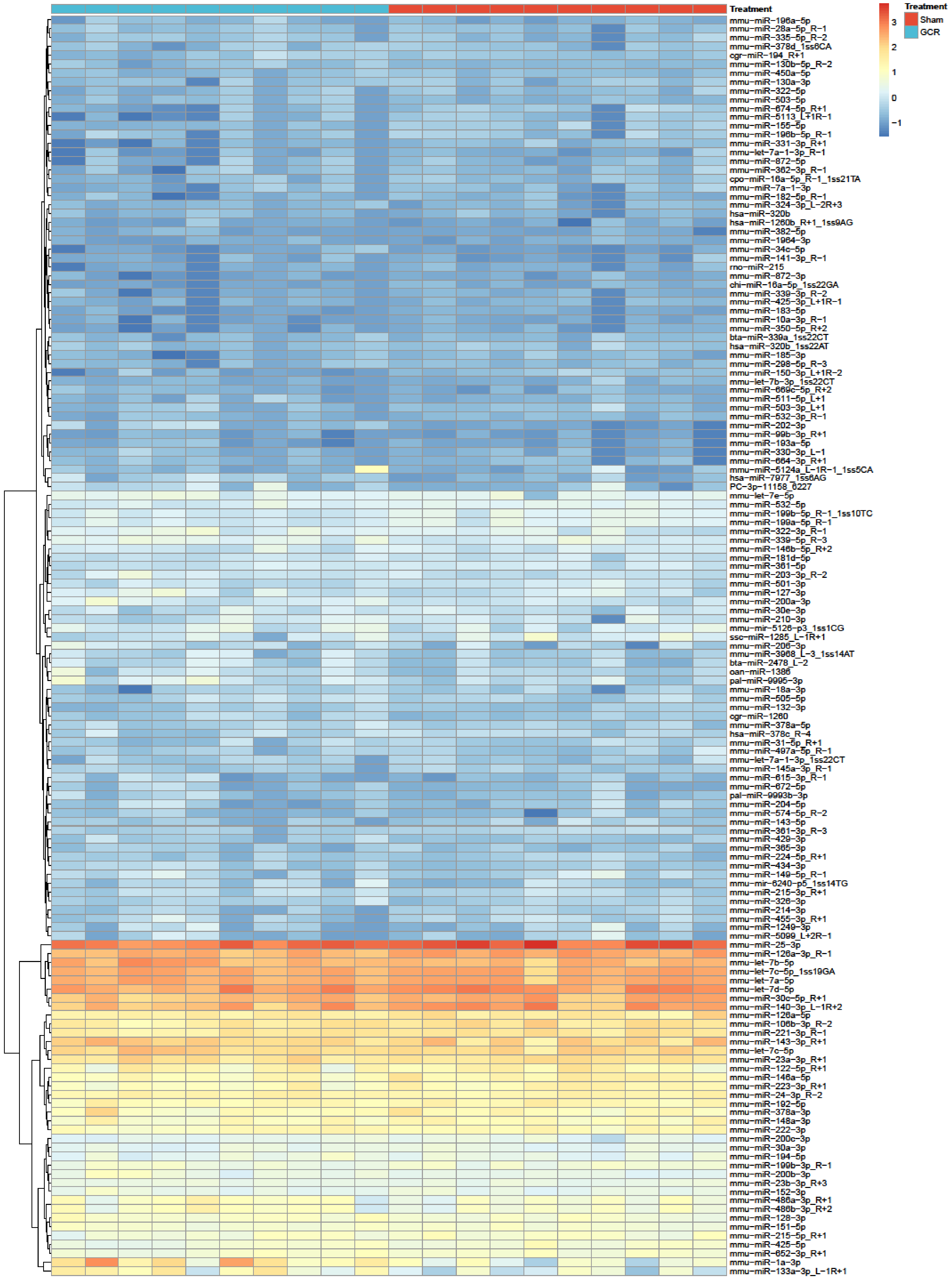
Treatment
Sham
GCR
3
2
1
0
-1
mmu-miR-196a-5p
mmu-miR-28a-5p_R-1
mmu-miR-335-5p_R-2
mmu-miR-378d_1ss6CA
cgr-miR-194_R+1
mmu-miR-130b-5p_R-2
mmu-miR-450a-5p
mmu-miR-130a-3p
mmu-miR-322-5p
mmu-miR-503-5p
mmu-miR-674-5p_R+1
mmu-miR-5113_L+1R-1
mmu-miR-155-5p
mmu-miR-196b-5p_R-1
mmu-miR-331-3p_R+1
mmu-let-7a-1-3p_R-1
mmu-miR-872-5p
mmu-miR-362-3p_R-1
cpo-miR-16a-5p_R-1_1ss21TA
mmu-miR-7a-1-3p
mmu-miR-182-5p_R-1
mmu-miR-324-3p_L-2R+3
hsa-miR-320b
hsa-miR-1260b_R+1_1ss9AG
mmu-miR-382-5p
mmu-miR-1964-3p
mmu-miR-34c-5p
mmu-miR-141-3p_R-1
rno-miR-215
mmu-miR-872-3p
chi-miR-16a-5p_1ss22GA
mmu-miR-339-3p_R-2
mmu-miR-425-3p_L+1R-1
mmu-miR-183-5p
mmu-miR-10a-3p_R-1
mmu-miR-350-5p_R+2
bta-miR-339a_1ss22CT
hsa-miR-320b_1ss22AT
mmu-miR-185-3p
mmu-miR-298-5p_R-3
mmu-miR-150-3p_L+1R-2
mmu-let-7b-3p_1ss22CT
mmu-miR-669c-5p_R+2
mmu-miR-511-5p_L+1
mmu-miR-503-3p_L+1
mmu-miR-532-3p_R-1
mmu-miR-202-3p
mmu-miR-99b-3p_R+1
mmu-miR-193a-5p
mmu-miR-330-3p_L-1
mmu-miR-664-3p_R+1
mmu-miR-5124a_L-1R-1_1ss5CA
hsa-miR-7977_1ss6AG
PC-3p-11158_6227
mmu-let-7e-5p
mmu-miR-532-5p
mmu-miR-199b-5p_R-1_1ss10TC
mmu-miR-199a-5p_R-1
mmu-miR-322-3p_R-1
mmu-miR-339-5p_R-3
mmu-miR-146b-5p_R+2
mmu-miR-181d-5p
mmu-miR-361-5p
mmu-miR-203-3p_R-2
mmu-miR-501-3p
mmu-miR-127-3p
mmu-miR-200a-3p
mmu-miR-30e-3p
mmu-miR-210-3p
mmu-mir-5126-p3_1ss1CG
ssc-miR-1285_L-1R+1
mmu-miR-206-3p
mmu-miR-3968_L-3_1ss14AT
bta-miR-2478_L-2
oan-miR-1386
pal-miR-9995-3p
mmu-miR-18a-3p
mmu-miR-505-5p
mmu-miR-132-3p
cgr-miR-1260
mmu-miR-378a-5p
hsa-miR-378c_R-4
mmu-miR-31-5p_R+1
mmu-miR-497a-5p_R-1
mmu-let-7a-1-3p_1ss22CT
mmu-miR-145a-3p_R-1
mmu-miR-615-3p_R-1
mmu-miR-672-5p
pal-miR-9993b-3p
mmu-miR-204-5p
mmu-miR-574-5p_R-2
mmu-miR-143-5p
mmu-miR-361-3p_R-3
mmu-miR-429-3p
mmu-miR-365-3p
mmu-miR-224-5p_R+1
mmu-miR-434-3p
mmu-miR-149-5p_R-1
mmu-mir-6240-p5_1ss14TG
mmu-miR-215-3p_R+1
mmu-miR-326-3p
mmu-miR-214-3p
mmu-miR-455-3p_R+1
mmu-miR-1249-3p
mmu-miR-5099_L+2R-1
mmu-miR-25-3p
mmu-miR-126a-3p_R-1
mmu-let-7b-5p
mmu-let-7c-5p_1ss19GA
mmu-let-7a-5p
mmu-let-7d-5p
mmu-miR-30c-5p_R+1
mmu-miR-140-3p_L-1R+2
mmu-miR-126a-5p
mmu-miR-106b-3p_R-2
mmu-miR-221-3p_R-1
mmu-miR-143-3p_R+1
mmu-let-7c-5p
mmu-miR-23a-3p_R+1
mmu-miR-122-5p_R+1
mmu-miR-146a-5p
mmu-miR-223-3p_R+1
mmu-miR-24-3p_R-2
mmu-miR-192-5p
mmu-miR-378a-3p
mmu-miR-148a-3p
mmu-miR-222-3p
mmu-miR-200c-3p
mmu-miR-30a-3p
mmu-miR-194-5p
mmu-miR-199b-3p_R-1
mmu-miR-200b-3p
mmu-miR-23b-3p_R+3
mmu-miR-152-3p
mmu-miR-486a-3p_R+1
mmu-miR-486b-3p_R+2
mmu-miR-128-3p
mmu-miR-151-5p
mmu-miR-215-5p_R+1
mmu-miR-425-5p
mmu-miR-652-3p_R+1
mmu-miR-1a-3p
mmu-miR-133a-3p_L-1R+1

d

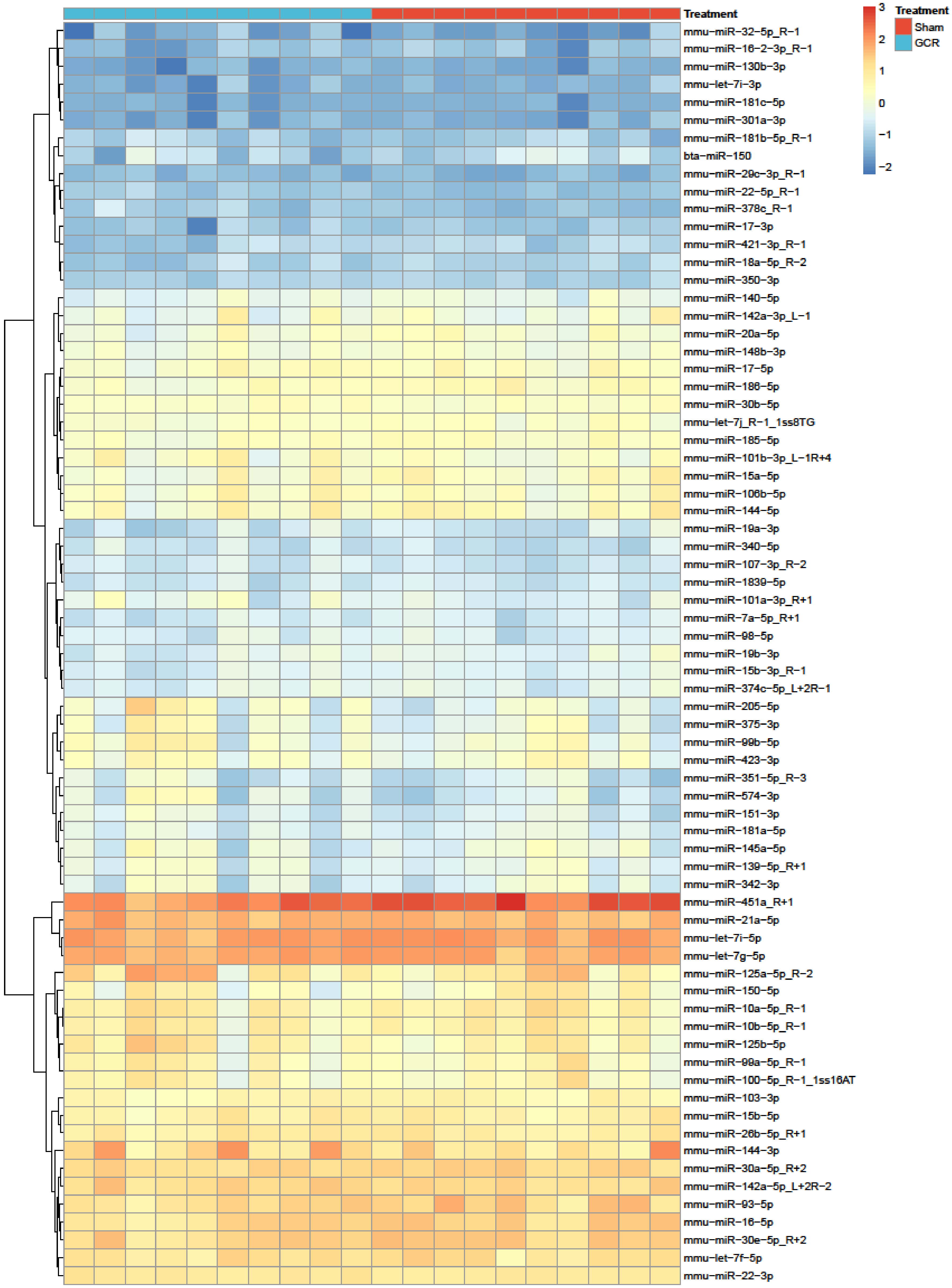
Treatment
Sham
GCR
3
2
1
0
−1
−2
mmu−miR−32−5p_R−1
mmu−miR−16−2−3p_R−1
mmu−miR−130b−3p
mmu−let−7i−3p
mmu−miR−181c−5p
mmu−miR−301a−3p
mmu−miR−181b−5p_R−1
bta−miR−150
mmu−miR−29c−3p_R−1
mmu−miR−22−5p_R−1
mmu−miR−378c_R−1
mmu−miR−17−3p
mmu−miR−421−3p_R−1
mmu−miR−18a−5p_R−2
mmu−miR−350−3p
mmu−miR−140−5p
mmu−miR−142a−3p_L−1
mmu−miR−20a−5p
mmu−miR−148b−3p
mmu−miR−17−5p
mmu−miR−186−5p
mmu−miR−30b−5p
mmu−let−7j_R−1_1ss8TG
mmu−miR−185−5p
mmu−miR−101b−3p_L−1R+4
mmu−miR−15a−5p
mmu−miR−106b−5p
mmu−miR−144−5p
mmu−miR−19a−3p
mmu−miR−340−5p
mmu−miR−107−3p_R−2
mmu−miR−1839−5p
mmu−miR−101a−3p_R+1
mmu−miR−7a−5p_R+1
mmu−miR−98−5p
mmu−miR−19b−3p
mmu−miR−15b−3p_R−1
mmu−miR−374c−5p_L+2R−1
mmu−miR−205−5p
mmu−miR−375−3p
mmu−miR−99b−5p
mmu−miR−423−3p
mmu−miR−351−5p_R−3
mmu−miR−574−3p
mmu−miR−151−3p
mmu−miR−181a−5p
mmu−miR−145a−5p
mmu−miR−139−5p_R+1
mmu−miR−342−3p
mmu−miR−451a_R+1
mmu−miR−21a−5p
mmu−let−7i−5p
mmu−let−7g−5p
mmu−miR−125a−5p_R−2
mmu−miR−150−5p
mmu−miR−10a−5p_R−1
mmu−miR−10b−5p_R−1
mmu−miR−125b−5p
mmu−miR−99a−5p_R−1
mmu−miR−100−5p_R−1_1ss16AT
mmu−miR−103−3p
mmu−miR−15b−5p
mmu−miR−26b−5p_R+1
mmu−miR−144−3p
mmu−miR−30a−5p_R+2
mmu−miR−142a−5p_L+2R−2
mmu−miR−93−5p
mmu−miR−16−5p
mmu−miR−30e−5p_R+2
mmu−let−7f−5p
mmu−miR−22−3p

e

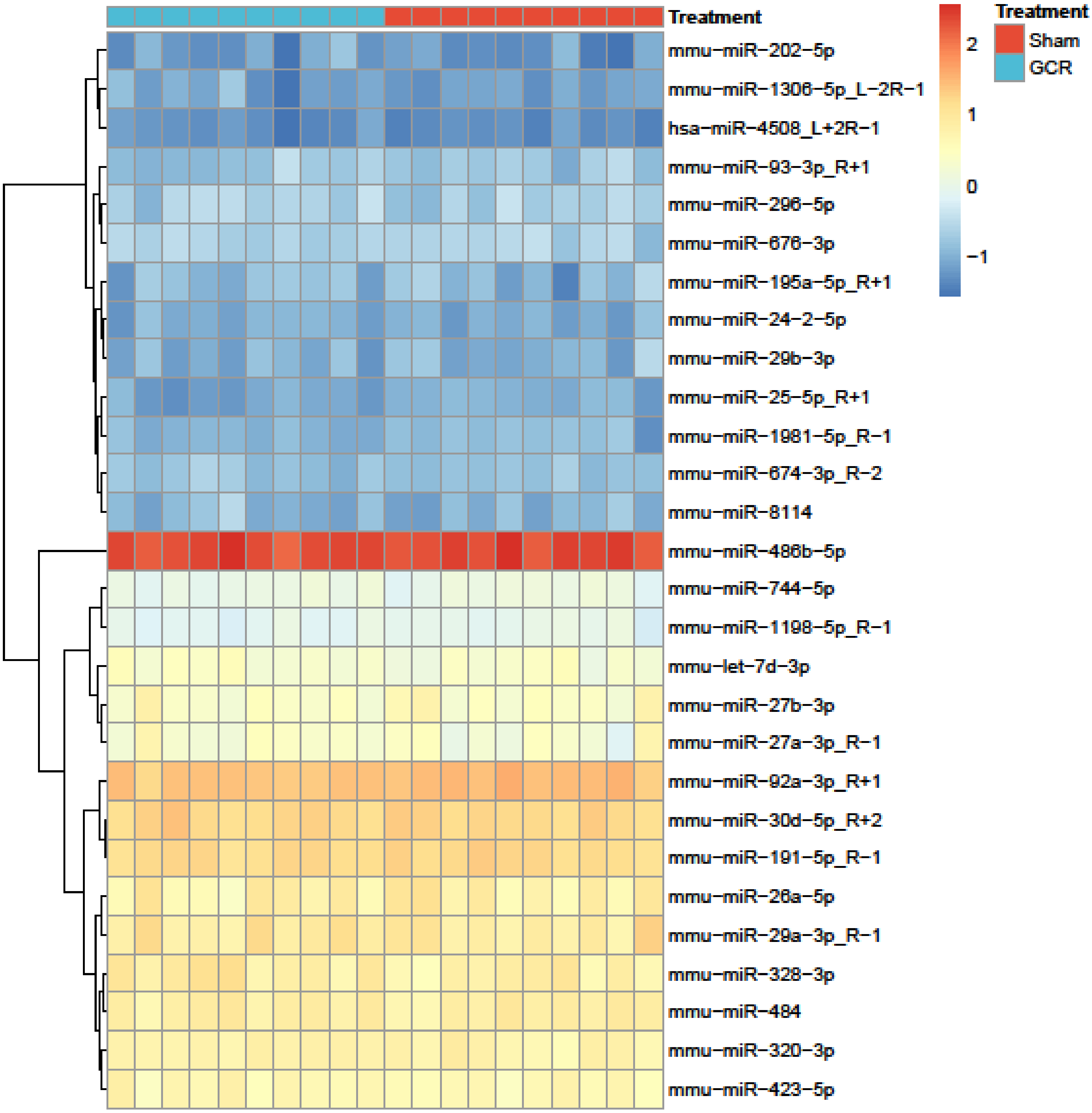
Treatment
mmu-miR-202-5p
mmu-miR-1306-5p_L-2R-1
hsa-miR-4508_L+2R-1
mmu-miR-93-3p_R+1
mmu-miR-296-5p
mmu-miR-676-3p
mmu-miR-195a-5p_R+1
mmu-miR-24-2-5p
mmu-miR-29b-3p
mmu-miR-25-5p_R+1
mmu-miR-1981-5p_R-1
mmu-miR-674-3p_R-2
mmu-miR-8114
mmu-miR-486b-5p
mmu-miR-744-5p
mmu-miR-1198-5p_R-1
mmu-let-7d-3p
mmu-miR-27b-3p
mmu-miR-27a-3p_R-1
mmu-miR-92a-3p_R+1
mmu-miR-30d-5p_R+2
mmu-miR-191-5p_R-1
mmu-miR-26a-5p
mmu-miR-29a-3p_R-1
mmu-miR-328-3p
mmu-miR-484
mmu-miR-320-3p
mmu-miR-423-5p
2
1
0
-1
Treatment
Sham
GCR

Supplementary Figure 9: Kidney miRNA quantitative ISH

BNL-3 simGCRsim-exposed mouse (~1.5-year dose equivalent) kidney sections were stained for **a,** miR-16 and **b,** Let-7a. Data are mean ± SD. A P-value of <0.05 was considered significant. OSOM; outer stripe of outer medulla. ISOM; inner stripe of outer medulla. IM; Inner medulla. Average; the simple arithmetic mean of the four anatomical regions.

a

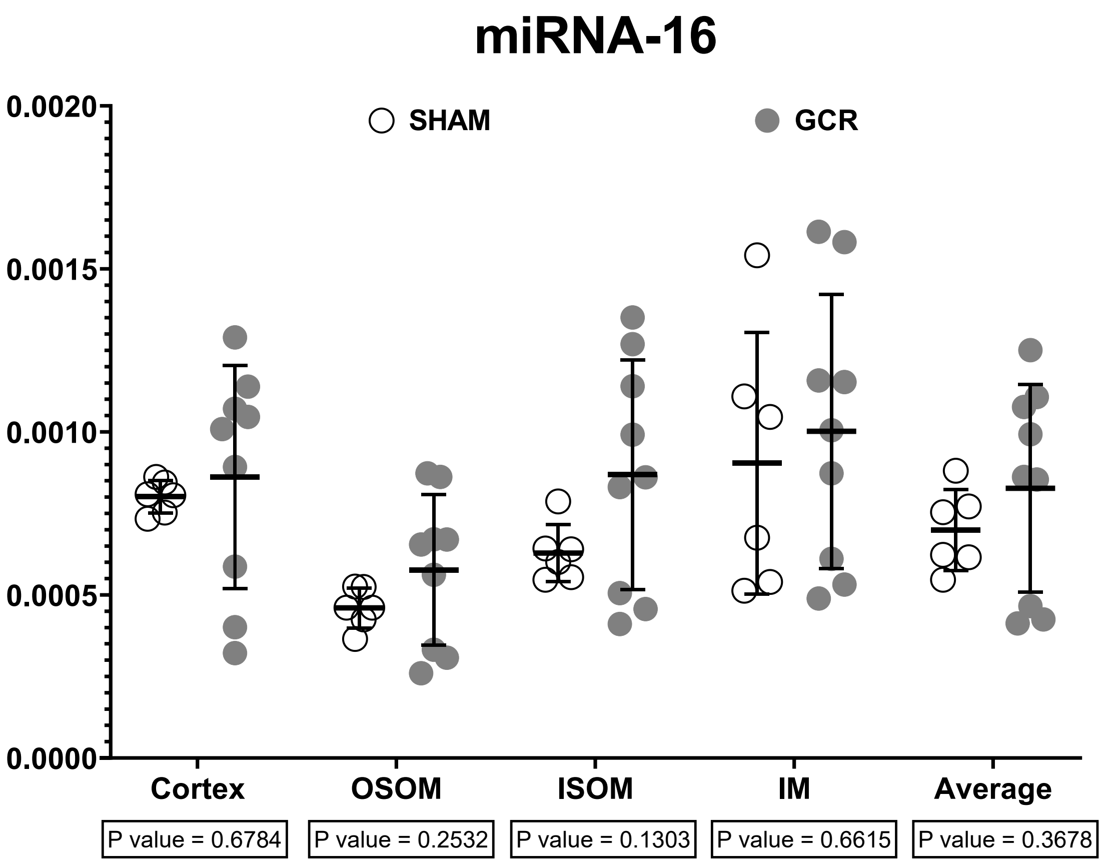

miRNA-16
SHAM
GCR
0.0020
0.0015
0.0010
0.0005
0.0000
Cortex
OSOM
ISOM
IM
Average
P value = 0.6784
P value = 0.2532
P value = 0.1303
P value = 0.6615
P value = 0.3678

b

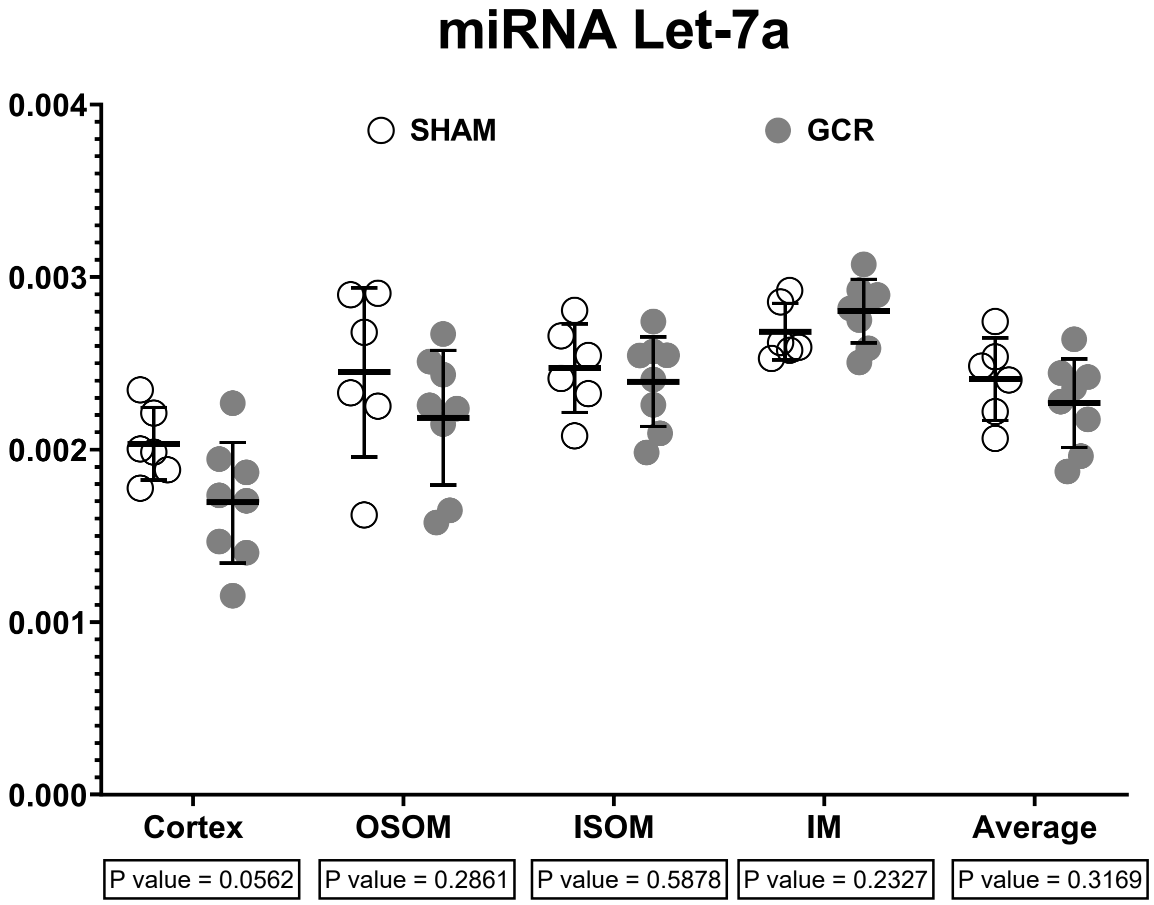

miRNA Let-7a
SHAM
GCR
0.004
0.003
0.002
0.001
0.000
Cortex
OSOM
ISOM
IM
Average
P value = 0.0562
P value = 0.2861
P value = 0.5878
P value = 0.2327
P value = 0.3169

Supplementary Figure 10: Mouse plasma and urine physiological measurements

a, Urinary and **b,** plasma physiological measurements from NSRL22A GCRsim-exposed mice (~2.5-year dose equivalent). Boxed P-values report the two-way ANOVA treatment group factor result. Data are mean ± SD. A P-value of <0.05 was considered significant. FE; fraction excretion. Corr; corrected for albumin. Cr; creatinine. K; potassium. Mg; magnesium. Cl; chloride. KIM-1; kidney injury marker-1.

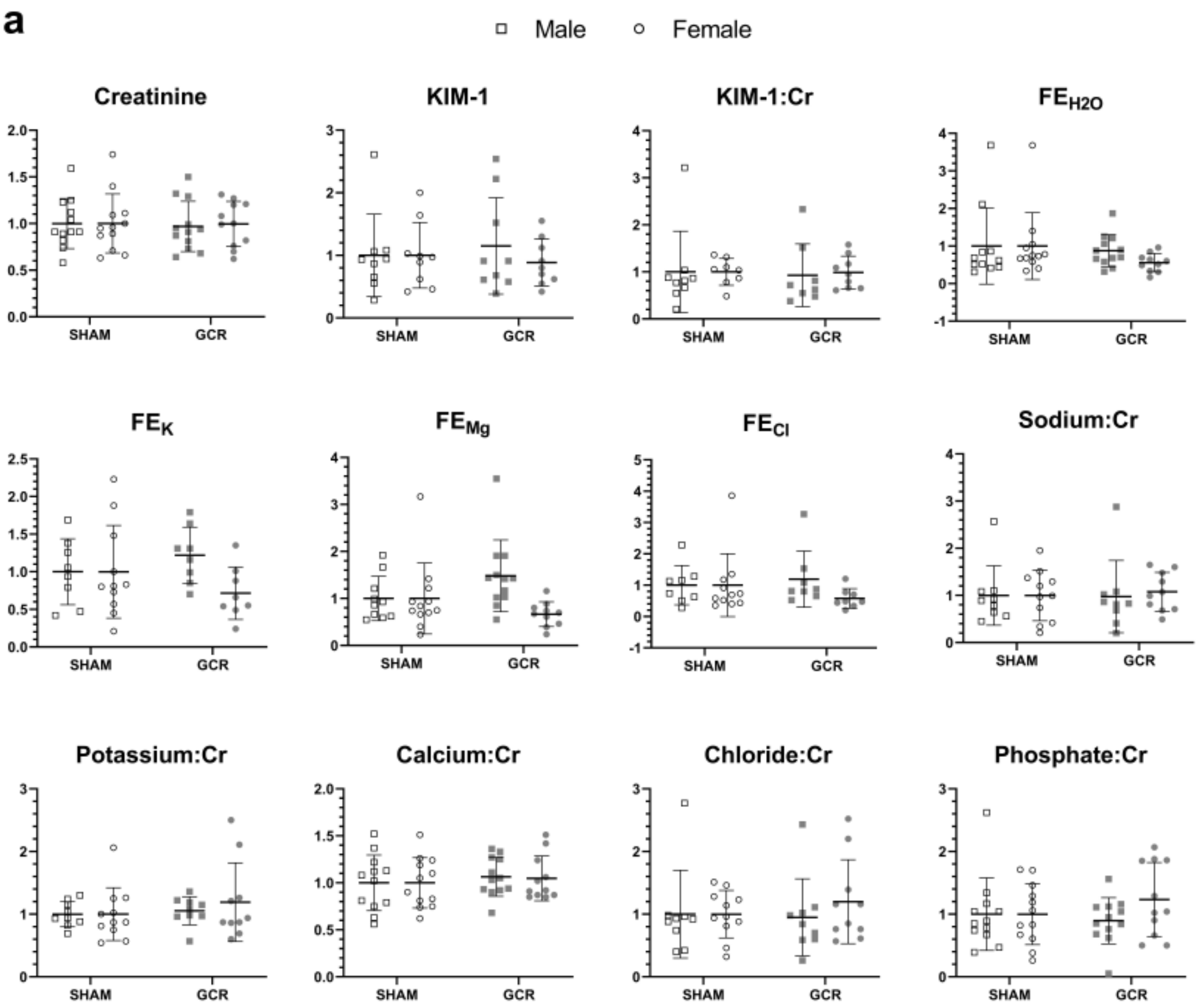

a
Male
Female
Creatinine
KIM-1
KIM-1:Cr
FE H2O
FE K
FE Mg
FE Cl
Sodium:Cr
Potassium:Cr
Calcium:Cr
Chloride:Cr
Phosphate:Cr
SHAM
GCR

b

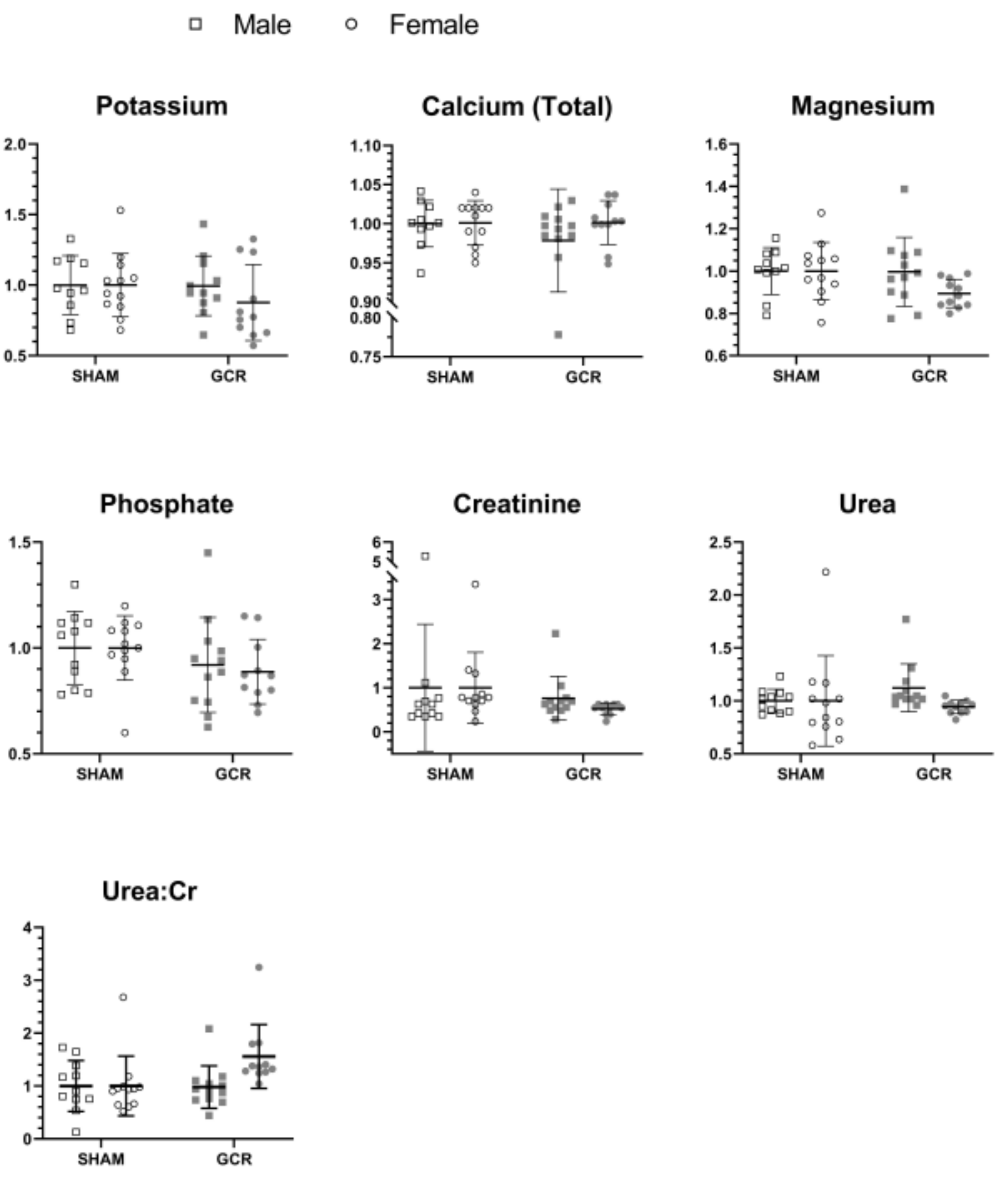
Male
Female
Potassium
2.0
1.5
1.0
0.5
SHAM
GCR
Calcium (Total)
1.10
1.05
1.00
0.95
0.90
0.80
0.75
SHAM
GCR
Magnesium
1.6
1.4
1.2
1.0
0.8
0.6
SHAM
GCR
Phosphate
1.5
1.0
0.5
SHAM
GCR
Creatinine
6
5
3
2
1
0
SHAM
GCR
Urea
2.5
2.0
1.5
1.0
0.5
SHAM
GCR
Urea:Cr
4
3
2
1
0
SHAM
GCR

Supplementary Methods

Experimental Design, Considerations and Limitations

For all animal studies, only wildtype and untreated/vehicle control animals were used for data analysis. To maintain consistency and comparability, when studies had serial longitudinal measurements in both control and exposure-treated animals, the control and treatment groups were directly compared rather than comparing the differences from baseline. Where feasible to do so, persons involved in data acquisition or analysis were kept blinded to group identity to minimise the risk of investigator bias.

In the absence of in-flight measurements (L+ or FD+) we chose to look at post-flight (R+) timepoints as close to splashdown as possible to capture spaceflight phenomena with minimal influence from readaptation to microgravity.

Most spaceflight harvested samples would have gone through freeze-thaw cycles, however the quality of data was assessed, and any unsuitable datasets or samples were excluded on this technical basis. Another caveat that should be noted is that kidney that omics from RR-1, RR-3, RR-7, RR-23 animals were performed on different 1/6th portions cut from the kidney. While these portions likely came from the same anatomical region for each omics modality, it is likely that this introduced increased intra-assay variability due to over or under representation of different anatomical regions arising from difficulties in dissecting perfectly reproducible 1/6th portions form the kidney. And as the kidney is relatively heterogeneous organ, it is likely inter-assay comparisons may be confounded by differences in anatomical regions present in different 1/6th portions from the same kidney. All other missions' whole or hemi-sected or hemisected kidneys were processed

under dry-ice/liquid nitrogen to generate a homogenised powder to ensure all anatomical regions were proportionally represented and results from assays could be more accurately compared to one another.

Ethics

All human and animal experiments were approved by institutional animal care and use committees (IACUC/AREC) and human research ethics committees (HREC). In line with the principles of the 3Rs, extant data and tissues were used where possible to reduce the use of additional animals. Human data was anonymised, and group data averages were used where possible to maintain anonymity.

Epigenome

Whole-genome bisulfite sequencing

Cohort	Specimen	Experiment	Raw Data	Processed	Analysis
RR-1	Kidney	OSD-102 [1]	OSD-102 [1]	Silveira *et al., 2020* [2]	Silveira *et al., 2020* [2]
RR-3	Kidney	OSD-163 [3]	OSD-163 [3]	Silveira *et al., 2020* [2]	Silveira *et al., 2020* [2]

Transcriptome

Bulk RNA sequencing

Cohort	Specimen	Experiment	Raw Data	Processed	Analysis
RR-1	Kidney	OSD-102 [1]	OSD-102 [1]	OSD-102 [1]	OSD-102 [1]
RR-3	Kidney	OSD-163 [3]	OSD-163 [3]	OSD-163 [3]	OSD-163 [3]
RR-7	Kidney	OSD-253 [4]	OSD-253 [4]	OSD-253 [4]	OSD-253 [4]
RR-10	Kidney	OSD-462 [5]	OSD-462 [5]	OSD-462 [5]	OSD-462 [5]
RR-23	Kidney	OSD-513 [6]	OSD-513 [6]	OSD-513 [6]	OSD-513 [6]

MHU-3	Kidney	Suzuki *et al., 2020* [7], OSD-457 [8]	OSD-457 [8]	GSE152382 [9]	See below
MHU-1	Plasma	Shiba *et al., 2017* [10], OSD-532 [11]	OSD-532 [11]	GSE213808 [12]	GSE213808 [12], See below
JAXA	Plasma	OSD-530 [13], *Muratani et al., 2024* [14]	Not available	OSD-530 [13], *Muratani et al., 2024* [14]	OSD-530 [13], *Muratani et al., 2024* [14], See below

Differential abundance analysis

MHU-3

Through the Jupyter platform, accessing the Science Managed Cloud Environment (SMCE) database, the default settings were used to generate differential expression analysis, normalization, and volcanoplot using the DESeq2 library (v 1.38.3) [15]in R language (v 4.2). Gene annotation for the differential expression analysis was performed with the bioMart library (v 3.16)[16,17] to access the Ensembl database, searching for mouse-specific genes (mmusculus_gene_ensembl).

MHU-1

Only ground control and microgravity groups were compared for the analysis.

JAXA

Reads in FASTQ files were imported into CLC Genomics Workbench (CLC-GW, ver.10.1.1, Qiagen), mapped to the human (hg19) reference genome, and quantified using a 57,773-gene (human) downloaded from the CLC-GW server to obtain the total count values, which were combined into a table. To plot normalized expression values, total counts were normalized by the scaling option in CLC-GW (normalization value = mean, reference = median mean, trimming 5%). Normalized total count values were log-2 transformed after adding a pseudocount of unity. ANOVAs and Empirical analyses of DGEs with pairwise EDGE test comparisons were performed

in CLC-GW to obtain the fold-change, weighted difference, nominal P-value and FDR-corrected P-value. For the analysis, samples from timepoints L-168d, L-112d and L-56d were pooled and averaged into “pre-flight” for pairwise comparison to either L+5d or L+120d.

Bulk small RNA sequencing

Cohort	Specimen	Experiment	Raw Data	Processed	Analysis
BNL-1	Plasma	*Malkani et al., 2020* [18], OSD-336 [19]	OSD-336 [19]	*Wuu et al., 2020* [20]	See below

miRNA WGCNA and target pathway analysis

The R package weighted correlation network analysis (WGCNA)[21] was used to find clusters (ME; module-eigengenes) of highly correlated plasma miRNA transcripts with a high likelihood of co-expression and similar biological function. Dendrograms and heatmaps were generated for all MEs and aggregate differential abundances were calculated for each ME comparing Sham to GCR-exposed animals.

The miRNAs in these MEs were then input into MicroRNA ENrichment TURned NETwork (MIENTURNET[22]) webtool to perform TargetScan-based miRNA-target Enrichment analysis using the default settings. This produced a list of mRNAs that were targeted by at least miRNAs within the MEs, and those with a nominal P-value <0.05 underwent DisGeNET[23] or KEGG [24]pathway using Metascape[25] with default settings. Mouse genes were imported as mouse and analysed as human. This was repeated for miR-125b and miR-16.

Spatial RNA sequencing

10um sections of fresh-frozen axial kidney sections from BNL-1 sham and GCR-exposed mice (n=3 per group) were processed according to the Slide-seqV2 protocol[26]. Samples were sequenced on an Illumina NovaSeq S2 flow cell 200 cycle kit with a read structure of 43 bases for read 1, 8 bases for the i7 index read and 60 bases for read 2. Each puck received ~340 million reads, corresponding to ~3,400 to ~5,100 reads per bead. Transcripts were then mapped back to bead coordinates and cell type classifications were assigned to each bead (and colour-coded) with the robust cell type decomposition (RCTD) method[27] using a pre-existing mouse kidney cell atlas[28].

For differential abundance analysis of transcripts per cell type, all beads of a given class were summed from across n=3 pucks per groups, means were then $\log_2$ transformed and DESeq2[15] was used to calculate log-fold change, nominal p-values and adjusted p-values. Those with adjusted p-values <0.05 were listed in **Fig.9D**, and then for each cell type those transcripts with a nominal P-value <0.05 underwent DisGeNET[23] or KEGG[24] pathway using Metascape[25] with default settings. Mouse genes were imported as mouse and analysed as human.

qPCR

Pre-validated SYBR® green PrimePCR™ primers (Bio-Rad) were used for qPCR analysis of reference genes (*B2m, Rps18, Tfrc, Tbp, Ube2d2*), mitochondrial fission markers (*Dnm1l, Fis1, Mff*) and fusion markers (*Mfn1, Mfn2, Opa1*) in RNA extracted from RR-10 whole kidney homogenate[5]. Reference gene stability and normalisation factor calculations were done in RefFinder[29]. Normalised relative abundances were then calculated for each of the fusion and fission markers for RR-10 ground control (n=7) vs spaceflight mice (n=8). To look for relative changes in all markers of fission and fusion, the geometric mean was calculated for both the fission and fusion markers, separately for each animal, and the arithmetic mean of these was then used to compare between groups in GraphPad Prism 9 (v.9.5.1).

The following primers were used:

Gene - assay ID:

Dnm1l - qMmuCID0021702

Fis1 - qMmuCID0020479

Mff - qMmuCID0017784

Mfn1 - qMmuCID0022027

Mfn2 - qMmuCID0023456

Opa1 - qMmuCID0010500

B2m - qMmuCID0040553

Rps18 - qRnoCID0057002

Tbp - qMmuCID0040542

Tfrc - qMmuCID0039655

Ube2d2 - qMmuCID0025334

Proteome

Quantitative fractionated TMT-labelled DDA LC-MS/MS

Cohort	Specimen	Experiment	Raw Data	Processed	Analysis
RR-1	Kidney	OSD-102 [1]	OSD-102 [1]	Silveira *et al., 2020* [2]	Silveira *et al., 2020* [2]
RR-3	Kidney	OSD-163 [3]	OSD-163 [3]	Silveira *et al., 2020* [2]	Silveira *et al., 2020* [2]
RR-10	Kidney	OSD-462 [5]	OSD-462 [5]	OSD-462 [5]	OSD-462 [5]

Data acquisition and processing

Either fresh-frozen whole (BNL-3) or axially hemisected kidneys (NSRL-22A), were homogenised in 1 mL of 1 ml lysis buffer (8M urea, 2M thiourea, Complete protease inhibitor (#1186153001) Phostop protease inhibitor (PHOSS-RO, both Merck)) using the Precellys 24

Tissue homogeniser (6500 rpm, 1x 15 secs) with Lysing Matrix beads (#6913100, MPI Bio). After centrifugation at 15,000 rpm for 5 mins to pellet cellular debris, supernatant was removed and stored at -80ºC. 0.5 mL of guanidine hydrochloride buffer (4M GuHCl, 50mM sodium acetate, 25 mM EDTA in ddH_2O, pH 5.8) was added to the pellet with a metal bead (#69989, 5mm, Qiagen) and vortexed at 1300 rpm for 1 hr at room temperature. Once the metal bead was removed, 1.5 ml of ice-cold ethanol was added to the supernatant, vortexed and incubated at -20ºC overnight. Precipitates were pelleted at 15,000 rpm for 5 mins, and the ethanol was carefully removed, before drying in a SpeedVac. The original supernatant was then thawed and combined with the dried precipitated pellet material, vortexed well to solubilise and centrifuged again at 15,000 rpm for 5 mins. Protein concentration was quantified with a Bradford assay.

In-solution reduction, alkylation and digestion with trypsin was performed according to a routine digestion protocol prior to subsequent analysis by mass spectrometry. Cysteine residues were reduced with dithiothreitol and derivatised by treatment with iodoacetamide to form stable carbamidomethyl derivatives. Trypsin digestion was carried out overnight at room temperature after initial incubation at 37ºC for 2 hours. The digested samples were cleaned up with PR-C18 resins. The cleaned peptide samples were resuspended in loading buffer for LC-MS/MS analysis.

For TMT labelling the cleaned peptide samples were resuspended in 100mM TEAB and labelled with *TMTpro* tags based on the user guide protocol. TMT tag labelling efficiency check showed >99%, and all 16plex tags were combined as one TMTpro set. Three TMTpro sets were generated, with a master pooled reference standard labelled with the 134N tag, allowing up to 45 experimental samples to be investigated that were randomised across the tags and sets (but allowing for equal proportions of each group per set).

In order to explore deeper into the proteome, a fractionation of TMTpro labelled peptide mix prior to LCMS analysis was performed using a high pH reversed phase HPLC. We used an Ultimate 3000 HPLC equipped with a degasser and a UV detector. Mobile phase A was 0.1%

triethylamine (aq), Mobile phase B was 0.1% triethylamine in acetonitrile. Peptides were subjected to a ZORBAX 300Extend-C18 column (4.6 mm inner diameter x 150 mm length, 3.5 μm particle size, part no. 763973-902, Agilent Technologies) and eluted out in a 20-min long two-step linear gradient from 0% to 50% for 9 min and from 50% to 90% buffer B for 1 min and keeping 90% buffer B for another 1 min, at a flow rate of 0.4 ml/min. The peptide mixture was fractionated into a total of 16 fractions, which were consolidated into 8 super-fractions. Peptide fractions were dried using a vacuum centrifuge and resuspended in 2% acetonitrile, 0.05% TFA (aq).

Chromatographic separation was performed using an Ultimate 3000 NanoLC system (ThermoFisherScientific, UK). Peptides were resolved by reversed phase chromatography on a 75μm*50cm C18 column using a three-step gradient of water in 0.1% formic acid (A) and 80% acetonitrile in 0.1% formic acid (B). The gradient was delivered to elute the peptides at a flow rate of 250 nl/min over 120 min. The eluate was ionised by electrospray ionisation using an Orbitrap Fusion Lumos (ThermoFisherScientific, UK) operating under Xcalibur v4.4. The instrument was programmed to acquire MS data using "Synchronous Precursor Selection with Multi-notch MS3" method (SPS MS3) by defining a 3s cycle time among a full MS scan, MS/MS fragmentation and MS3 fragmentation. We acquired one full-scan MS spectrum at a resolution of 120,000 at 200 m/z with 100% normalized AGC target and a scan range of 400~1500m/z and maximum injection time 100 ms. The MS/MS fragmentation was conducted using collision-induced dissociation (CID) and quadrupole ion trap analyzer. Parameters were set up as 100% normalized AGC target, NCE (normalized collision energy) 35, q-value 0.25, isolation window 1.2 Th and maximum injection time 50 ms. The MS3 scan was analyzed using higher-energy collision dissociation (HCD) and Orbitrap analyzer with a synchronous precursor selection. Parameters included number of SPS 10, NCE 65, 400% normalized AGC target, maximum injection time 105 ms, resolution 50,000 at 200 Th, isolation window 1.6. 2-6 charged states were defined within this method.

Raw mass spectrometry data were processed into peak list files using Proteome Discoverer (ThermoScientific; v2.5) (PD 2.4). Processed data was then searched using Sequest search

engine embedded in PD 2.5, against the current version of the reviewed Swissprot *Mouse* database downloaded from Uniprot[30]. The following parameters were used:

Spectrum Selector

Min. Precursor mass:	350 Da
Max. Precursor mass:	10000 Da
S/N Threshold (FT-only): 1.5	

Mascot/Sequest

Database:	Uniprot
Enzyme:	Trypsin
Missed cleavage:	2
Precursor mass tol:	10 ppm
Fragment mass tol:	0.6 Da
Dynamic modifications:	
	Carbamidomethyl (C)
	Oxidation (M)

Peptide validator

Target FDR (strict):	0.01
Target FDR (relaxed):	0.05

TMT Reporter Quantification

Co-Isolation Threshold.:	50
SPS Mass Matches[%] Threshold: 50 (65 as default)	
Average Reporter S/N Threshold: 10	

Quantitative label-free SEER DIA LC-MS/MS

Cohort	Specimen	Experiment	Raw Data	Processed	Analysis
Inspiration4	Plasma	OSD-571 [31]	By Request	OSD-571 [31]	OSD-571 [31], See “DAA” below
Inspiration4	Exosome	OSD-571 [32]	By Request	OSD-571 [32]	OSD-571 [32], See “DAA” below

Plasma Slow Off-Rate Modified Aptamers

Cohort	Specimen	Experiment	Raw Data	Processed	Analysis
RR-19	Plasma	*Lee et al., 2020* [33], OSD-342 [34]	OSD-342 [34]	OSD-342 [34]	See “DAA”

Shotgun LC-MS/MS

Cohort	Specimen	Experiment	Raw Data	Processed	Analysis
Cosmonauts	Urine	*Pastushkova et al., 2013* [35]	PASS00239 [36]	PASS00239 [36]	See below

<u>Network Analysis</u>

Cosmonaut (n=10 per timepoint) urinary shotgun proteomes pre-flight were compared with those measured post-flight R+1d and R+7d. Proteins that were absent pre-flight but detected after spaceflight were taken forward for network analysis in Cytoscape StringApp[37] using the latest version of the STRING[38] database. This was then visualised in Cytoscape using the yFiles Tree layout, excluding singletons.

Differential abundance analysis (DAA)

In-house R scripts were used for proteomics data processing and statistical analysis. The corrected reporter intensity values were used to analyse proteomics data. Protein groups containing matches to decoy database or contaminants were discarded. Total intensity for each reporter were calculated and matched to correct for the sample loads in each experiment. Only proteins that were quantified in the pooled samples were used for the analysis. Subsequently, internal reference scaling (IRS) method was employed to normalize protein intensities between different runs using common proteins in pooled internal standards. The data was log2 transformed and scaled by subtracting the median for each sample. LIMMA was employed to determine differentially abundant proteins between groups.

Inspiration4

For the analysis, samples from timepoints L-92d, L-44d and L-3d were pooled and averaged into "pre-flight" for comparison to immediate post-flight R+1d.

Epiproteome

Quantitative fractionated TMT-labelled DDA LC-MS/MS of phospho-enriched peptides

Cohort	Specimen	Experiment	Raw Data	Processed	Analysis
RR-10	Kidney	OSD-462 [5]	OSD-462 [5]	OSD-462 [5]	OSD-462 [5], See below

Differential abundance analysis

In-house R scripts were used for phosphoproteomics data processing and statistical analysis. Only phosphopeptides with a single modified residue were considered for further analysis. These phosphopeptides were mapped back to genes with the residue amino acid and position annotated. The data was log2 transformed and LIMMA was employed to determine differentially abundant phosphopeptides between groups. For analyses that required single entries for each gene, the phosphopeptides were collapsed into a single gene and their intensity scores aggregated to obtain a generalised change in the overall phospho-status of the protein encoded by that gene.

Metabolome

Quantitative label-free UHPLC-MS/MS

Cohort	Specimen	Experiment	Raw Data	Processed	Analysis
Inspiration4	Plasma	OSD-571 [39]	By Request	OSD-571 [39]	OSD-571 [39], See below
MHU-3	Plasma	*Suzuki et al., 2020* [7,] *Uruno et al., 2021* [40]	By Request	ibSLS [41]	See below
CNSA	Serum	*Zhang et al., 2020* [42]	By Request	*Zhang et al., 2020* [42]	*Zhang et al., 2020* [42], See below

Differential abundance analysis

Inspiration4

For the analysis, samples from timepoints L-92d, L-44d and L-3d were pooled and averaged into "pre-flight" for comparison to immediate post-flight R+1d.

MHU-3

For the analysis, samples from ground control and spaceflight animals were directly compared for timepoints L+18d and R+2d. Data were Log_2 transformed, fold changes were calculated, and nominal p-values and adjusted p-values were determined in GraphPad Prism 8 using the multiple tests option.

Over-representation analysis

Metabolites with a nominal P-value of <0.05 and which had a successful name check were taken forward for KEGG module pathway analysis (targeted) using MetaboAnalyst 5.0[43] with the default settings.

Microbiome

Whole metagenome shotgun sequencing

Cohort	Specimen	Experiment	Raw Data	Processed	Analysis
RR-6	Faeces	OSD-249 [44]	OSD-249 [44]	See "DP"	See "DAA"
RR-9	Faeces	OSD-250 [45]	OSD-250 [45]	See "DP"	See "DAA"
RR-10	Faeces	OSD-466 [46]	OSD-465 [46]	See "DP"	See "DAA"
RR-23	Faeces	OSD-465 [47]	OSD-465 [47]	See "DP"	See "DAA"

16S rRNA gene amplicon sequencing

Cohort	Specimen	Experiment	Raw Data	Processed	Analysis
RR-1	Faeces	*Jiang et al., 2019* [48]	OSD-212 [49]	*Jiang et al., 2019* [48]	*Jiang et al., 2019* [48]
STS-135	Faeces	*Ritchie et al., 2015* [50]	OSD-72 [51]	*Ritchie et al., 2015* [50]	*Ritchie et al., 2015* [50]

Data processing (DP) and differential abundance analysis (DAA)

Sequence data were processed using the Nephele platform[52], provided by the National Institute of Allergy and Infectious Diseases (NIAID) Office of Cyber Infrastructure and Computational Biology (OCICB) in Bethesda, MD (1). The Whole metaGenome Sequence Assembly pipeline, version 2 (WGSA2), was employed to assess whole-genome shotgun sequencing data. Additionally, pre-processed amplicon sequencing taxa abundance data from RR-1 and STS-135 were obtained from previous publications.

Data analysis was conducted using R (Version 4.2.1). Centred log-ratio transformed abundance values of the observed taxa detected in over 10% of the samples were calculated. Differential abundance between mission-matched Flight and Ground Control groups was evaluated using a linear model, followed by a Benjamini-Hochberg False Discovery Rate correction. Relevant R libraries utilised in this analysis can be found online[53,54].

For a comprehensive multi-omics analysis, total microbiome differential abundance results were employed. A kidney stone (KS) relevant microbe list was compiled from existing literature, which

was then used for targeted differential abundance assessment. Directionality, degree, and significance of difference measures between groups for each mission were compiled for comparative purposes.

Physiology

Sample collection and processing

NSRL-22A

Urine collection was performed in awake mice to obtain spontaneous and uncontaminated samples. Mice were gently handled over a sheet of Saran® wrap or Parafilm® to facilitate micturition. Upon collection, each urine sample was carefully divided into two aliquots. One aliquot was immediately acidified with HNO3 to achieve a final concentration of 1% v/v, preventing precipitation of electrolytes and maintaining sample integrity. Both aliquots were then promptly stored at −80°C to preserve their biochemical constituents until further analysis. For blood collection, mice were subjected to terminal anaesthesia to minimize distress and ensure compliance with ethical guidelines. Blood samples were drawn from the vena cava using a fine needle and syringe. Plasma was separated by transferring the blood into Microvette® CB 300 LH (Sarstedt) tubes and centrifuging at 2000 g for 5 min. The resulting plasma supernatant was carefully aspirated and stored at −80°C for future analysis. Both plasma and non-acidified urine samples were analysed for creatinine levels by employing either the standard Jaffe reaction or the enzymatic method, depending on the investigator's preference and laboratory resources. Electrolyte levels, biochemistries and biomarkers were assessed using the Siemens Dimension RxL Max Integrated Chemistry System, at the Core Biochemical Assay Laboratory at Addenbrooke's Hospital, Cambridge, UK.

Inspiration4

Plasma samples collected from astronauts at timepoints L-92d, L-44d and L-3d were grouped and averaged into “pre-flight” for comparison against individual post-flight timepoints R+1d, R+45d, R+82d and R+194d. These samples were analysed by Quest Diagnostics using the Comprehensive metabolic panel [CMP; CPT code 80053].

NASA

A historical collection of archived astronaut frozen plasma and urine aliquots were collected and stored at -80C over many years. Clinical chemistries, endocrine profiles and biomarkers were obtained at the Nutritional Biochemistry Laboratory at NASA Johnson Space Center. Timepoints L-180d and L-45d were grouped and averaged as “pre-flight” for comparison to individual timepoints L+15d, L+30d, L+60d, L+120d, L+180d, R+0d and R+30d.

Functional calculations

eGFR was calculated using the new 2021 CKD-EPI creatinine (2009 CKD-EPI creatinine fit without race) [new eGFRcr(AS) equation[55]]. The fractional excretions of electrolytes were determined by (Urine solute x Plasma creatinine) ÷ (Urine creatinine x Plasma solute). A corrected FE_{Mg} was determined, as ~30% of magnesium is protein bound and is not filtered, and therefore the plasma magnesium must be multiplied by 0.7 i.e. [pCr × uMg] / [(0.7 × sMg) × pCr][56]. The FE_{H2O} is the volume of water that appears as urine compared to the amount filtered. Thus, FE_{H2O} = V / GFR. Since GFR = UCr x V / PCr. FE_{H2O} = V x PCr / UCr x V. Simplifying, FE_{H2O} = PCr / UCr (then multiply by 100 to express as a percentage[57]). The ratio of tubular maximum reabsorption of phosphate (TmP) to GFR is used to evaluate renal phosphate transport. TmP/GFR is calculated using the following steps: 1) Calculate the ratio of phosphate clearance to creatinine clearance (CP/CCr) CP/CCr = serum creatinine x Urine phosphate / Urine creatinine x Serum phosphate (This ratio is normally less than 0.15 and is often elevated in primary hyperparathyroidism). 2) Subtract this fraction from 1.0 to give the fractional tubular reabsorption

of phosphate (TRP). TRP = 1 - serum creatinine x Urine phosphate / Urine creatinine x Serum phosphate. 3) If TRP is $\leq$ 0.86 then phosphate reabsorption is maximal and there is a linear relationship between plasma phosphate concentration and excretion and TmP/GFR which is calculated by: TmP/GFR = TRP x serum phosphate. 4) If TRP is > 0.86 relationship between plasma phosphate concentration and excretion is curvilinear and TmP/GFR is defined as follows: TmP/GFR = α x serum phosphate, where α = 0.3 x TRP 1- (0.8 x TRP)[58]. The transtubular potassium gradient (TTKG) is used to gauge renal potassium secretion by the cortical collecting duct, indirectly assessing mineralocorticoid bioactivity in patients. TTKG = $U_K/P_K \times P_{Osm}/U_{Osm}$[59]. Estimated serum osmolality was determined using the Khajuria and Krahn equation: OSMc = 1.86(Na + K) + 1.15(Glu / 18) + (Urea / 6) + 14[60]. Free water clearance is calculated as $C_{H2O} = V(1-U_{Osm}/P_{Osm}$[61]).

Multi-omic integrated pathway over-representation analyses

To integrate datasets from across different omics, species, missions and tissues, all biomolecules (e.g. phosphoproteins, proteins, transcripts and methylated DNA) were linked back to their HGNC gene symbol, aggregated and collapsed to single genes (e.g. multiple phosphosites, isoforms, CpG sites) and converted to the human orthologs where necessary. To perform over-representation analysis in Metascape[25] using any of the pathway databases, the less stringent nominal p-value of $P<0.05$ was used to threshold differentially expressed hits within each dataset for analysis (with the exception of RR-23 where the adjusted p-value was used to threshold the number of hits to a manageable number), with a $-\text{Log}_{10}$(P-value) of 2 being considered significant for ontological term enrichment. This approach reduces the risk of false-negatives due to potentially overly stringent multiple comparison adjustment of the p-value at an individual dataset level, while mitigating the risk of false-positives at the convergence of hits during ontological term enrichment. The degree of confidence in the validity of results can then be drawn from the robustness of replication across orthogonal and/or independent datasets.

DisGeNET[23] analysis was performed using Metascape with default settings. Differentially expressed gene lists (p.value <0.05) from each dataset were imported into Metascape. Mouse genes were imported as mouse and analyzed as human. DisGeNET outputs containing enriched human disease terms (-LogP >2) were downloaded and visualized using Python's seaborn and matplotlib libraries. The circle size represents –LogP values and the circle color represents enrichment ratio.

KEGG pathway analysis[24] was performed using Metascape with default settings. Differentially expressed gene lists (p. value < 0.05) from each dataset were imported into Metascape. Mouse genes were imported as mouse and analyzed as human. KEGG outputs containing enriched KEGG pathways (-LogP >2) were downloaded and visualized using Python's seaborn and matplotlib libraries. The circle size represents –LogP values and the circle colour represents enrichment ratio.

Enriched GO[62] terms were obtained from clusterProfiler outputs. GO enrichments containing biological process ontologies were visualized using Python's seaborn and matplotlib libraries. The circle size represents –LogP values and the circle color represents enrichement ratio.

For analysis of significant genes (p. value < 0.05) that had a consensus in directionality, we only used kidney proteomic and transcriptomic datasets and ranked according to the $\log_2$ fold change direction and the number of times observed across all datasets. The highest ranked upregulated and downregulated genes were plotted using CoMut[63].

Integration of multi-omics and physiological data

The Scalable Precision Medicine Open Knowledge Engine (SPOKE)[64] is a population-level heterogeneous knowledge graph. SPOKE has distilled and connected information from over 40 databases into a structured graph with 21 different node types and 55 edge types. The databases

cover numerous biomedical domains, from basic science to clinical research. Previously, SPOKE was leveraged to analyse GeneLab gene expression data from mice flown in space. Using only data from mice, this study uncovered biological and clinical changes experienced by human astronauts.

Due to the heterogeneous structure of SPOKE, it is possible to harmonise multi-omics datasets. This is achieved by identifying “entry points” for each dataset. Entry points represent the logical overlap between SPOKE nodes and the entity being quantified in the dataset. Here entry points consisted of Gene (transcriptome and epigenome), Protein (proteome and post-translation modification), and Compound (metabolome) nodes. Once the external datasets were connected to SPOKE, Degree Weighted Path Count (DWPC) was used to score the connectivity between each entry node and the nodes in SPOKE. The top 5% of nodes from each entry point were merged into a single graph. The interconnected paths between the entry points provide a system-level view of how space flight impacts the kidneys. The resulting knowledge map figures highlight shared paths that traverse nodes involved in kidney morphogenesis, kidney stones, and chronic kidney disease.

Imaging

Sample fixation and processing

Mouse kidney samples were harvested and fixed through immersion in freshly prepared 4% (w/v) formaldehyde-PBS solution (pH 6.9) for 16 hours at 37°C. Subsequently, the samples were washed three times with PBS with 0.02% Na-Azide and stored at 4°C until they were embedded in paraffin.

Histopathology and Brightfield WSI

2-3 µm FFPE sections from NSRL-22A kidneys were prepared, deparaffinised using Histoclear (National Diagnostics), and rehydrated through a series of graded methanol steps. These were then stained for either Haematoxylin & Eosin (#ab245880, Abcam) or Masson's Trichrome (#ab150686, Abcam) according to the manufacturers guidelines.

Sections underwent brightfield whole slide imaging with 20X objective tiled and stitched focus stacks taken for each section on a Zeiss Axio Scan.Z1 slide scanner. The resultant images were viewed in QuPath (v0.4.3) and semi-quantitatively scored for any histopathological findings.

Immunostaining and Confocal WSI

5 µm FFPE sections from RR-10 kidneys were prepared, deparaffinised using Histoclear (National Diagnostics), and rehydrated through a series of graded methanol steps. Antigen retrieval was performed using [1X] R-Universal buffer (AP0530) in a 2100 antigen retriever for a single heat-pressure cycle (Aptum Biologics). Sections were then permeabilized with 0.05% (v/v) Triton X-100-PBS solution for 20 minutes and incubated with Section Block 'ready-to-use' (AP0471; Aptum Biologics) for 30 mins at RT. Primary antibodies were incubated overnight at 4°C for 16 hours at specified concentrations below, diluted in Antibody Diluent 'FF/PE Sections' (AP0472; Aptum Biologics). For phospho-specific antibodies, 10 µg/ml of the non-phospho peptide used to raise the antibody was added per 2 µg/ml of the antibody used. Negative control samples omitted the primary antibody and were processed simultaneously. Following incubation, slides were washed by dipping slides 50x times in 0.05% (v/v) Triton X-100-PBS for 3 rounds, and incubated with secondary antibodies for 1 hour at RT. Pre-absorbed fluorochrome conjugated secondary antibodies were utilised at the concentrations below, diluted in Antibody Diluent 'FF/PE Sections' (AP0472; Aptum Biologics). After washing, slides were mounted using Prolong Gold antifade (#P36930, Life Technologies), permitted to cure for 48-72hrs, sealed with CoverGrip Coverslip Sealant (#23005; Biotium) and protected from light exposure.

Immunofluorescent images were captured using either a Zeiss LSM700 LED laser-scanning confocal microscope or Leica SP8 white-light laser-scanning confocal microscope using 488-nm, 555-nm and 639-nm laser lines, employing a 10X/0.3NA (Zeiss) or 10X/0.5NA (Leica) objective. Zeiss acquisition parameters included single field of view, 8-bit resolution, 1844 x 1844 pixels, 1x digital zoom, 3.85μs pixel dwell time, 4-line Kalman filtering, sequential (by line) channel imaging, and a 4-slice z-stack with a 6 μm thickness to account for chromatic aberration.

Leica acquisition parameters included tile scanning with a 10% overlap, 8-bit resolution, 1024 × 1024 pixels, 0.75x digital zoom, 600 Hz scan speed, 6-line Kalman filtering, sequential (by line) channel imaging, and a 3-slice z-stack with a 5 μm thickness to account for chromatic aberration.

Images were processed using FIJI image analysis software (v.1.53p). Fluorescent z-stacks underwent background subtraction (200 px radius rolling ball) and maximum intensity z-projection. Brightness and contrast adjustments were made using linear histogram stretching to enhance visibility.

PRIMARIES

Target: anti-rat total NCC (tNCC) [cross reacts with mouse and human total NCC]

Host species: Rabbit

Clonality: pAb full IgG

Product: Abcam (ab95302)

Lot #: GR3274565-9

Working Concentration: @2ug/mL

Validation: Previously published in two models that increase or decrease NCC expression (https://doi.org/10.1093/hmg/ddv185 and https://doi.org/10.15252/emmm.201505444)

Target: anti-human phospho-NCC (pNCC) Thr46, Thr50, Thr55 [cross reacts with mouse pNCC Thr44, Thr48, Thr53]

Host species: Sheep

Clonality: pAb full IgG

Product: MRC PPU Reagents and Services (S908B)

Lot #: 2nd Bleed

Working Concentration: @2ug/mL (+ 10ug/mL non-phosphopeptide to ensure specificity)

Validation: Previously published in model that decreases NCC phosphorylation (https://doi.org/10.1093/hmg/ddv185)

Target: anti-human phospho-NCC (pNCC) Thr60 [cross reacts with mouse pNCC Thr58]

Host species: Sheep

Clonality: pAb full IgG

Product: MRC PPU Reagents and Services (S995B)

Lot #: 1st Bleed

Working Concentration: @2ug/mL (+ 10ug/mL non-phosphopeptide to ensure specificity)

Validation: Previously published in model that increase or decreases NCC phosphorylation (https://doi.org/10.1093/hmg/ddv185 and https://doi.org/10.15252/emmm.201505444)

SECONDARIES

Target: Alexa Fluor 647 anti-goat IgG (cross reacts with sheep IgG)

Host species: Donkey

Clonality: pAb IgG Fab Fragment

Product: Jackson ImmunoResearch (711-607-003)

Working Concentration: @8ug/mL

Validation: (lots of citations) https://www.jacksonimmuno.com/catalog/products/711-607-003

Target: Alexa Fluor 647+ anti-Goat IgG (cross reacts with sheep IgG)

Host species: Donkey

Clonality: pAb full IgG

Product: ThermoFisher (A32849)

Working Concentration: @10ug/mL

Validation: (lots of citations) https://www.thermofisher.com/antibody/product/Donkey-anti-Goat-IgG-H-L-Highly-Cross-Adsorbed-Secondary-Antibody-Polyclonal/A32849

Target: Alexa Fluor 555+ anti-rabbit IgG

Host species: Donkey

Clonality: pAb full IgG

Product: ThermoFisher (A32794)

Working Concentration: @10ug/mL

Validation: (lots of citations) https://www.thermofisher.com/antibody/product/Goat-anti-Rabbit-IgG-H-L-Highly-Cross-Adsorbed-Secondary-Antibody-Polyclonal/A32794

Target: Alexa Fluor 488 anti-rabbit IgG

Host species: Donkey

Clonality: pAb IgG Fab Fragment

Product: Jackson ImmunoResearch (711-547-003)

Working Concentration: @8ug/mL

Validation: (lots of citations) https://www.jacksonimmuno.com/catalog/products/711-547-003

miRNA in situ hybridisation and brightfield WSI

FFPE blocks for BNL-3 kidneys were trimmed to remove tissue exposed to air, and fresh 5μm sections were taken. These were not baked onto the slide and stored with desiccant instead. Samples were then processed using the miRNAscope™ HD RED assay (#324531 and #324500,

ACD-Biotechne) with the FFPE tissue section workflow according to the manufacturers guidelines. miRNAscope™ Probes against the following were used: mmu-miR-125b-5p (#1082311-S1), mmu-let-7a-5p (#727761-S1) and mmu-miR-16-5p (729031-S1).

Sections underwent brightfield whole slide imaging with 20X objective tiled and stitched focus stacks (with extended depth of focus) taken for each section on a Zeiss Axio Scan.Z1 slide scanner. Using QuPath (v0.4.3) the cortex, outer stripe of the outer medulla, inner stripe of the outer medulla and inner medulla were manually annotated with the aid of a Wacom cintiq pro 32 to create a segmentation mask of the anatomical regions for area calculation. Ilastik (v1.4.0)[65] automated (supervised) pixel-level classification was then trained to identify positive miRNAscope probe staining. A custom in-house python script was then used to count all positive staining and calculate the area of all anatomical regions, so that the density of each miRNA per unit area of each region could be determined for each kidney section.

Optical Clearing and 3D imaging

<u>Tissue transformation, delipidation and refractive index matching</u>

Quartered formaldehyde-fixed kidneys from RR-10 mice were tissue transformed using the SHIELD protocol[66] and reagents from Lifecanvas technologies (MA, USA) based on (10.1038/nbt.4281). SHIELD-transformed kidneys were delipidated (Lifecanvas Technologies; Full Passive Pipeline Protocol v4.06) for approximately 10–14 days at 37°C in 40 mL of passive delipidation buffer with gentle agitation in EasyClear device. Following delipidation, tissues were washed in several rounds of PBS at 37˚C overnight to remove any delipidation buffer. Before imaging tissues were immersed in Incubated in EasyIndex RI 1.53 refractive index matching solution as per protocol. Tissues were then embedded in 2% w/v ultra-low melting point agarose (Sigma A5030) blocks made up with EasyIndex solution to immobilise the samples. These were then stored in EasyIndex in airtight containers shielded from light prior to imaging.

MesoSPIM imaging

Samples were mounted in quartz cuvettes and immersion oil with refractive indices matching that of EasyIndex, and then suspended and aligned for light-sheet fluorescence imaging with a MesoSPIM[67]. Images were captured at 16-bit for 1-channel dual laser lightsheet illumination (488 nm excitations) to obtain autofluorescence emissions at 2048 x 2048 pixels in the XY, with a Z depth range of 700-1200 pixels, giving a pixel resolution of 3.26 μm (XY) and 4.0 μm (Z).

Image analysis

To evaluate any qualitative changes in gross morphology images were imported into Syglass [68] (v.1.7.2-79; https://www.syglass.io/; RRID: SCR_017961) for visual investigation by nephrologists and histopathologists in 3D virtual space using Meta Quest 2 VR headsets. Images were also imported into Imaris [69](v10.0) for visualisation on a Wacom cintiq pro 32” 4K touchscreen monitor. 3D video renders were later generated with Syglass (v.2.0.0) and Z-slice video created with FIJI (ImageJ; v.1.54h).

Morphometry

Normalised kidney weights

PI and NASA GeneLab records for all animals included in the study were examined for bodyweight and kidney wet weight measurements. Only BNL-1, BNL-2, BNL-3 and RR-23 missions had complete kidney and body weight information available. Where available, the weights of both left and right kidneys were averaged, and the kidney weights were normalised against bodyweight for the same animal and expressed as a percentage. All ground control and

sham animals that received no exposure treatment were grouped into control for pairwise comparisons against GCR (animals that only received either full or simplified galactic cosmic radiation simulations) or MG (animals that only underwent hindlimb unloading microgravity simulation) or GCR + MG (animals that underwent a combination of GCR and MG or were exposed to spaceflight).

Histomorphometry

Whole slide images of tiled immunofluorescent confocal images taken from RR-10 were used for analysis. Using QuPath (v0.4.3) the cortex was manually annotated with the aid of a Wacom cintiq pro 32 to create a segmentation mask of the major anatomical regions for area calculation (e.g. cortex vs medulla). Similarly distal convoluted tubules (DCT) labelled with antibodies against total NCC / phospho-NCC, the canonical DCT marker, were annotated. Initially, only a handful of tubules were annotated from each slide, and these were then used to train Ilastik (v1.4.0)[65] for automated (supervised) pixel-level classification of DCTs. These were then further manually refined to remove/include any false-positive/negatives. A custom in-house python script was then used to compute the number of discrete tubules positive for DCT markers, the corresponding area of each of these as well as the total cortex area, such that average tubule area and DCT density per area of cortex could be determined for each kidney section.

REFERENCES

1. NASA OSDR: Open Science for Life in Space. https://osdr.nasa.gov/bio/repo/data/studies/OSD-102.

2. Silveira, W. A. da *et al.* Comprehensive Multi-omics Analysis Reveals Mitochondrial Stress as a Central Biological Hub for Spaceflight Impact. *Cell* **183**, 1185-1201.e20 (2020).
3. NASA OSDR: Open Science for Life in Space. https://osdr.nasa.gov/bio/repo/data/studies/OSD-163.
4. NASA OSDR: Open Science for Life in Space. https://osdr.nasa.gov/bio/repo/data/studies/OSD-253.
5. NASA OSDR: Open Science for Life in Space. https://osdr.nasa.gov/bio/repo/data/studies/OSD-462.
6. NASA OSDR: Open Science for Life in Space. https://osdr.nasa.gov/bio/repo/data/studies/OSD-513.
7. Suzuki, T. *et al.* Nrf2 contributes to the weight gain of mice during space travel. *Commun. Biol.* **3**, 1–14 (2020).
8. NASA OSDR: Open Science for Life in Space. https://osdr.nasa.gov/bio/repo/data/studies/OSD-457.
9. GEO Accession viewer. https://www.ncbi.nlm.nih.gov/geo/query/acc.cgi?acc=GSE152382.
10. Shiba, D. *et al.* Development of new experimental platform 'MARS'—Multiple Artificial-gravity Research System—to elucidate the impacts of micro/partial gravity on mice. *Sci. Rep.* **7**, 10837 (2017).
11. NASA OSDR: Open Science for Life in Space. https://osdr.nasa.gov/bio/repo/data/studies/OSD-532.
12. Masafumi, M. GEO Accession viewer. https://www.ncbi.nlm.nih.gov/geo/query/acc.cgi?acc=GSE213808.
13. NASA OSDR: Open Science for Life in Space. https://osdr.nasa.gov/bio/repo/data/studies/OSD-530.
14. Masafumi, M. CD36-associated extracellular mitochondria as a hallmark of space response.

15. Love, M. I., Huber, W. & Anders, S. Moderated estimation of fold change and dispersion for RNA-seq data with DESeq2. *Genome Biol.* **15**, 550 (2014).
16. Durinck, S., Spellman, P. T., Birney, E. & Huber, W. Mapping identifiers for the integration of genomic datasets with the R/Bioconductor package biomaRt. *Nat. Protoc.* **4**, 1184–1191 (2009).
17. Durinck, S. *et al.* BioMart and Bioconductor: a powerful link between biological databases and microarray data analysis. *Bioinformatics* **21**, 3439–3440 (2005).
18. Malkani, S. *et al.* Circulating miRNA Spaceflight Signature Reveals Targets for Countermeasure Development. *Cell Rep.* **33**, 108448 (2020).
19. NASA OSDR: Open Science for Life in Space. https://osdr.nasa.gov/bio/repo/data/studies/OSD-336.
20. Wuu, Y.-R. *et al.* LET-Dependent Low Dose and Synergistic Inhibition of Human Angiogenesis by Charged Particles: Validation of miRNAs that Drive Inhibition. *iScience* **23**, (2020).
21. Langfelder, P. & Horvath, S. WGCNA: an R package for weighted correlation network analysis. *BMC Bioinformatics* **9**, 559 (2008).
22. Licursi, V., Conte, F., Fiscon, G. & Paci, P. MIENTURNET: an interactive web tool for microRNA-target enrichment and network-based analysis. *BMC Bioinformatics* **20**, 545 (2019).
23. Piñero, J. *et al.* The DisGeNET knowledge platform for disease genomics: 2019 update. *Nucleic Acids Res.* **48**, D845–D855 (2020).
24. Kanehisa, M. & Goto, S. KEGG: Kyoto Encyclopedia of Genes and Genomes. *Nucleic Acids Res.* **28**, 27–30 (2000).
25. Zhou, Y. *et al.* Metascape provides a biologist-oriented resource for the analysis of systems-level datasets. *Nat. Commun.* **10**, 1523 (2019).
26. Stickels, R. R. *et al.* Highly sensitive spatial transcriptomics at near-cellular resolution with Slide-seqV2. *Nat. Biotechnol.* **39**, 313–319 (2021).

27. Cable, D. M. *et al.* Robust decomposition of cell type mixtures in spatial transcriptomics. *Nat. Biotechnol.* **40**, 517–526 (2022).

28. Miao, Z. *et al.* Single cell regulatory landscape of the mouse kidney highlights cellular differentiation programs and disease targets. *Nat. Commun.* **12**, 2277 (2021).

29. Xie, F., Wang, J. & Zhang, B. RefFinder: a web-based tool for comprehensively analyzing and identifying reference genes. *Funct. Integr. Genomics* **23**, 125 (2023).

30. The UniProt Consortium. UniProt: the Universal Protein Knowledgebase in 2023. *Nucleic Acids Res.* **51**, D523–D531 (2023).

31. Mason, C. E. Inspiration4 plasma proteomics. (2023).

32. Mason, C. E. Inspiration4 exosome proteomics. (2023).

33. Lee, S.-J. *et al.* Targeting myostatin/activin A protects against skeletal muscle and bone loss during spaceflight. *Proc. Natl. Acad. Sci.* **117**, 23942–23951 (2020).

34. NASA OSDR: Open Science for Life in Space. https://osdr.nasa.gov/bio/repo/data/studies/OSD-342.

35. Pastushkova, L. Kh. *et al.* Changes of Protein Profile of Human Urine after Long-Term Orbital Flights. *Bull. Exp. Biol. Med.* **156**, 201–204 (2013).

36. View a PASSEL Dataset. https://db.systemsbiology.net/sbeams/cgi/PeptideAtlas/PASS_View?identifier=PASS00239.

37. Doncheva, N. T., Morris, J. H., Gorodkin, J. & Jensen, L. J. Cytoscape StringApp: Network Analysis and Visualization of Proteomics Data. *J. Proteome Res.* **18**, 623–632 (2019).

38. Szklarczyk, D. *et al.* The STRING database in 2023: protein–protein association networks and functional enrichment analyses for any sequenced genome of interest. *Nucleic Acids Res.* **51**, D638–D646 (2023).

39. Mason, C. E. 39. Inspiration4 plasma metabolomics. (2023).

40. Uruno, A. *et al.* Nrf2 plays a critical role in the metabolic response during and after spaceflight. *Commun. Biol.* **4**, 1–18 (2021).

41. ibSLS - Metabolite List -. https://ibsls.megabank.tohoku.ac.jp/metabolite-list.
42. Zhang, H. *et al.* Serum Metabolomics Associating With Circulating MicroRNA Profiles Reveal the Role of miR-383-5p in Rat Hippocampus Under Simulated Microgravity. *Front. Physiol.* **11**, (2020).
43. Pang, Z. *et al.* MetaboAnalyst 5.0: narrowing the gap between raw spectra and functional insights. *Nucleic Acids Res.* **49**, W388–W396 (2021).
44. NASA OSDR: Open Science for Life in Space. https://osdr.nasa.gov/bio/repo/data/studies/OSD-249.
45. NASA OSDR: Open Science for Life in Space. https://osdr.nasa.gov/bio/repo/data/studies/OSD-250.
46. NASA OSDR: Open Science for Life in Space. https://osdr.nasa.gov/bio/repo/data/studies/OSD-466.
47. NASA OSDR: Open Science for Life in Space. https://osdr.nasa.gov/bio/repo/data/studies/OSD-465.
48. Jiang, P., Green, S. J., Chlipala, G. E., Turek, F. W. & Vitaterna, M. H. Reproducible changes in the gut microbiome suggest a shift in microbial and host metabolism during spaceflight. *Microbiome* **7**, 113 (2019).
49. NASA OSDR: Open Science for Life in Space. https://osdr.nasa.gov/bio/repo/data/studies/OSD-212.
50. Ritchie, L. E. *et al.* Space Environmental Factor Impacts upon Murine Colon Microbiota and Mucosal Homeostasis. *PLOS ONE* **10**, e0125792 (2015).
51. NASA OSDR: Open Science for Life in Space. https://osdr.nasa.gov/bio/repo/data/studies/OSD-72.
52. Weber, N. *et al.* Nephele: a cloud platform for simplified, standardized and reproducible microbiome data analysis. *Bioinformatics* **34**, 1411–1413 (2018).
53. thomazbastiaanssen. 0. Introduction. (2023).

54. Bastiaanssen, T. F. S., Quinn, T. P. & Loughman, A. Bugs as Features (Part I): Concepts and Foundations for the Compositional Data Analysis of the Microbiome-Gut-Brain Axis. (2022) doi:10.48550/arXiv.2207.12475.

55. Inker, L. A. *et al.* New Creatinine- and Cystatin C–Based Equations to Estimate GFR without Race. *N. Engl. J. Med.* **385**, 1737–1749 (2021).

56. Topf, J. M. & Murray, P. T. Hypomagnesemia and Hypermagnesemia. *Rev. Endocr. Metab. Disord.* **4**, 195–206 (2003).

57. Coulthard, M. G. & Haycock, G. B. Distinguishing between salt poisoning and hypernatraemic dehydration in children. *BMJ* **326**, 157–160 (2003).

58. Stark, H., Eisenstein, B., Tieder, M., Rachmel, A. & Alpert, G. Direct Measurement of TP/GFR: A Simple and Reliable Parameter of Renal Phosphate Handling. *Nephron* **44**, 125–128 (2008).

59. Choi, M. J. & Ziyadeh, F. N. The Utility of the Transtubular Potassium Gradient in the Evaluation of Hyperkalemia. *J. Am. Soc. Nephrol.* **19**, 424–426 (2008).

60. Martín-Calderón, J. L. *et al.* Choice of the best equation for plasma osmolality calculation: Comparison of fourteen formulae. *Clin. Biochem.* **48**, 529–533 (2015).

61. Free water clearance. *Wikipedia* (2021).

62. The Gene Ontology Consortium. The Gene Ontology resource: enriching a GOld mine. *Nucleic Acids Res.* **49**, D325–D334 (2021).

63. Crowdis, J., He, M. X., Reardon, B. & Van Allen, E. M. CoMut: visualizing integrated molecular information with comutation plots. *Bioinformatics* **36**, 4348–4349 (2020).

64. Morris, J. H. *et al.* The scalable precision medicine open knowledge engine (SPOKE): a massive knowledge graph of biomedical information. *Bioinformatics* **39**, btad080 (2023).

65. Berg, S. *et al.* ilastik: interactive machine learning for (bio)image analysis. *Nat. Methods* **16**, 1226–1232 (2019).

66. Park, Y.-G. *et al.* Protection of tissue physicochemical properties using polyfunctional crosslinkers. *Nat. Biotechnol.* **37**, 73–83 (2019).

67. Voigt, F. F. *et al.* The mesoSPIM initiative: open-source light-sheet microscopes for imaging cleared tissue. *Nat. Methods* **16**, 1105–1108 (2019).
68. Pidhorskyi, S., Morehead, M., Jones, Q., Spirou, G. & Doretto, G. syGlass: Interactive Exploration of Multidimensional Images Using Virtual Reality Head-mounted Displays. Preprint at https://doi.org/10.48550/arXiv.1804.08197 (2018).
69. Imaris Microscopy Image Analysis Software | Comparison - Imaris. *Oxford Instruments* https://imaris.oxinst.com/packages.
