# Supplementary material for: Cosmic kidney disease: an integrated pan-omic, physiological and morphological study into spaceflight-induced renal dysfunction
Source: Nat Commun. 2024 Jun 11;15:4923. doi: 10.1038/s41467-024-49212-1 (PMC11167060; doi:10.1038/s41467-024-49212-1)
Supplement: Supplementary file 3 — Description of Additional Supplementary Files [file 41467_2024_49212_MOESM3_ESM.pdf]

Description of Additional Supplementary Files

Supplementary Data 1: Kidney differentially abundant phosphosites and their functional annotations

Phosphopeptide-enriched phosphoproteomics from RR-10 spaceflight-exposed mice (28 days) kidney tissue, with functional annotations and inferences from PhosphoSitePlus and the literature. An adjusted P-value of <0.05 was considered significant.

Supplementary Data 2: Multi-mission multi-omic DisGeNET over-representation analysis results

Full list of enriched gene-disease associations are presented for DisGeNET ontological terms relating to a, Rank 1 - kidney health [kidney; urological; electrolyte; mitochondrial (general); complement; urinary system cancers; blood pressure (renal)], b, Rank 2 - closely related to kidney injury and disease [Vascular/endothelial; cardiovascular; autonomic dysfunction; diabetes; blood pressure (general)], c, Rank 3 - general diseases known to impact the kidney [non-urinary systems cancers; aging; inflammation; liver], d, Rank 4 - no clear connection to the kidney. To integrate datasets from different omics modalities, species, missions and tissues, all biomolecules (e.g. phosphopeptides, proteins, transcripts and methylated DNA) were converted to the human orthologs where necessary and linked back to their HGNC gene symbol, aggregated and collapsed to single genes (e.g. multiple phosphosites, isoforms, CpG sites). A Log10(P-value) of 2 was considered significant for ontological term enrichment and had to replicate in at least two datasets to be included. Enrichment (gene) ratio; the number of differentially regulated hits in a dataset that belong to a given ontological term, normalised to the total number of statistically significant hits in the respective dataset.

Supplementary Data 3: Up and down regulated kidney gene products and their functional annotations

Full list of upregulated and downregulated gene products in exposure groups (e.g. spaceflight, GCRsim) compared to control groups (e.g. ground control, sham). To integrate datasets from different omics modalities, species, missions and tissues, all biomolecules (e.g. phosphopeptides, proteins, transcripts and methylated DNA) were converted to the human orthologs where necessary and linked back to their HGNC gene symbol, aggregated and collapsed to single genes (e.g. multiple phosphosites, isoforms, CpG sites). These differentially regulated gene products (DRGP) were scored using the following rules: 1) only DRGPs with a P-value <0.05 were counted as significant and plotted; 2) Each DRGP was assigned a score of +1 each time it was upregulated or -1 each time it was downregulated; 3) only DRGPs observed in proteome and transcriptome kidney-specific datasets were used for the calculation of the ranking score to avoid confounders, although additional datasets (PTM and

epigenome and plasma/exosomes) were plotted for visualisation purposes; 4) The resulting sum of scores for each DRGP was then calculated and multiplied by the number of times the DRGP was observed in the kidney-specific proteome and transcriptome datasets; 5) Only product scores of absolute value 4 or higher were included plotted as these will have a directionality consensus of at least 2 datasets above the number of disagreeing datasets. The highest scoring genes have functional annotations and inferences about their consequences detailed.

Supplementary Data 4: Nephrolithiasis-related faecal microbiome differential abundances

Supplementary Data 5: Plasma Metabolome KEGG module metabolic pathway over-representation analysis results

The top enriched KEGG module pathway ontological terms. These were ranked and represented in descending order using the following rules: 1) No. of mission datasets it replicated in; 2) most significant p-value (no enrichment scores could be calculated). A Log10(P-value) of 1.3 was considered significant for ontological term enrichment and had to replicate in at least two datasets to be plotted. Plant-related terms removed from the display.

Supplementary Data 6: Multi-mission multi-omic Gene Ontology (GO) over-representation analysis top results

Full list of enriched GO ontological terms relating to a, biological process, b, cellular component c, molecular function are presented. To integrate datasets from different omics modalities, species, missions and tissues, all biomolecules (e.g. phosphopeptides, proteins, transcripts and methylated DNA) were converted to the human orthologs where necessary and linked back to their HGNC gene symbol, aggregated and collapsed to single genes (e.g. multiple phosphosites, isoforms, CpG sites). A Log10(P-value) of 2 was considered significant for ontological term enrichment and had to replicate in at least two datasets to be plotted. Enrichment (gene) ratio; the number of differentially regulated hits in a dataset that belong to a given ontological term, normalised to the total number of statistically significant hits in the respective dataset.

Supplementary Data 7: Multi-mission multi-omic KEGG pathway over-representation analysis results

Full list of enriched KEGG pathway ontological terms relating to a, kidney health or cellular injury/stress, b, closely related to kidney injury and disease [vasculature; blood pressure; bone; muscle; diabetes], c, no clear connection to the kidney, are presented. To integrate datasets from different omics modalities, species, missions and tissues, all biomolecules (e.g. phosphopeptides, proteins, transcripts and

methylated DNA) were converted to the human orthologs where necessary and linked back to their HGNC gene symbol, aggregated and collapsed to single genes (e.g. multiple phosphosites, isoforms, CpG sites). A Log10(P-value) of 2 was considered significant for ontological term enrichment and had to replicate in at least two datasets to be plotted. Enrichment (gene) ratio; the number of differentially regulated hits in a dataset that belong to a given ontological term, normalised to the total number of statistically significant hits in the respective dataset.

Supplementary Data 8: Plasma miRNA predicted mRNA targets and DisGeNET over-representation analysis results

WGCNA calculated plasma miRNA MEs from BNL-1 simGCRsim-exposed mice (~1.5-year dose equivalent) were used to generate a list of predicted mRNA targets using Mienturnet – TargetScan, and the subsequent outputs that met the threshold of a p-value <0.05 were used to run a DisGeNET over-representation analysis. A Log10(P-value) of 2 was considered significant for ontological term enrichment.

Supplementary Data 9: Kidney miRNA predicted mRNA targets and DisGeNET over-representation analysis

miR-125b and miR-16 used to generate a list of predicted mRNA targets using Mienturnet – TargetScan, and the subsequent outputs that met the threshold of a p-value <0.05 were used to run a DisGeNET over-representation analysis. A Log10(P-value) of 2 was considered significant for ontological term enrichment.

Supplementary Data 10: Kidney Spatial transcriptomics differential abundances, DisGeNET & KEGG over-representation analyses

BNL-1 simGCRsim-exposed mouse (~1.5-year dose equivalent) kidney sections were subjected to Slide-seq, and spatially mapped beads with several thousand transcripts were assigned a cell type classification. Differential abundances lists were generated for each cell type: DCT; distal convoluted tubule. Early PT; early proximal tubule S1. LOH; thick ascending limb of the loop of Henle. PC; principal cell. PCT; proximal convoluted tubule S1 + S2; Podo; podocyte. PST; proximal straight tubule S3. A p-value of <0.05 was considered significant, and these mRNA transcripts were taken forward for DisGeNET & KEGG over-representation analyses for each cell type. A Log10(P-value) of 2 was considered significant for ontological term enrichment.

Supplementary Movie 1: Gross kidney morphology in 3D

SHIELD-preserved kidneys from RR-10 spaceflight-exposed mice (28 days) were optically cleared (delipidated and refractive index matched). Presented are representative 3D video renders of mesoSPIM lightsheet 488-nm stimulated autofluorescence images of an exemplar ground control (left) and spaceflight (right)

sample. Initially these are displayed as mean intensity gradient projections pseudocoloured with a heatmap, these later transition into a grayscale absorption opacity model as an alternative visualisation of structures. Some artefacts (e.g. tissue splitting) due to mechanical damage sustained during post-mortem dissections are visible in both samples.

Supplementary Movie 2: Kidney nephron morphology viewed in the XY through Z-slices

SHIELD-preserved kidneys from RR-10 spaceflight-exposed mice (28 days) were optically cleared (delipidated and refractive index matched). Presented are representative 2D Z-stacks of mesoSPIM lightsheet 488-nm stimulated autofluorescence images of an exemplar ground control and spaceflight sample. Some artefacts (e.g. tissue splitting) due to mechanical damage sustained during post-mortem dissections are visible in both samples.
