# Supplementary material for: Cosmic kidney disease: an integrated pan-omic, physiological and morphological study into spaceflight-induced renal dysfunction
Source: Nat Commun. 2024 Jun 11;15:4923. doi: 10.1038/s41467-024-49212-1 (PMC11167060; doi:10.1038/s41467-024-49212-1)
Supplement: Supplementary file 16 — Reporting Summary [file 41467_2024_49212_MOESM16_ESM.pdf]

nature portfolio

Corresponding author(s): Keith Siew, Stephen Walsh

Last updated by author(s): 2024/05/18

Reporting Summary

Nature Portfolio wishes to improve the reproducibility of the work that we publish. This form provides structure for consistency and transparency in reporting. For further information on Nature Portfolio policies, see our Editorial Policies and the Editorial Policy Checklist.

Please do not complete any field with "not applicable" or n/a. Refer to the help text for what text to use if an item is not relevant to your study.
For final submission: please carefully check your responses for accuracy; you will not be able to make changes later.

Statistics

For all statistical analyses, confirm that the following items are present in the figure legend, table legend, main text, or Methods section.

n/a	Confirmed	
☐	☒	The exact sample size (*n*) for each experimental group/condition, given as a discrete number and unit of measurement
☐	☒	A statement on whether measurements were taken from distinct samples or whether the same sample was measured repeatedly
☐	☒	The statistical test(s) used AND whether they are one- or two-sided<br>*Only common tests should be described solely by name; describe more complex techniques in the Methods section.*
☐	☒	A description of all covariates tested
☐	☒	A description of any assumptions or corrections, such as tests of normality and adjustment for multiple comparisons
☐	☒	A full description of the statistical parameters including central tendency (e.g. means) or other basic estimates (e.g. regression coefficient) AND variation (e.g. standard deviation) or associated estimates of uncertainty (e.g. confidence intervals)
☐	☒	For null hypothesis testing, the test statistic (e.g. *F*, *t*, *r*) with confidence intervals, effect sizes, degrees of freedom and *P* value noted<br>*Give P values as exact values whenever suitable.*
☒	☐	For Bayesian analysis, information on the choice of priors and Markov chain Monte Carlo settings
☒	☐	For hierarchical and complex designs, identification of the appropriate level for tests and full reporting of outcomes
☒	☐	Estimates of effect sizes (e.g. Cohen's *d*, Pearson's *r*), indicating how they were calculated

Our web collection on statistics for biologists contains articles on many of the points above.

Software and code

Policy information about availability of computer code

Data collection	No software was used for data collection
Data analysis	DESeq2 library (v 1.38.3); R language (v 4.2); bioMart library (v 3.16); (CLC-GW, ver.10.1.1, Ojagen); WGCNA; MIENTURNET; GraphPad Prism 9 (v.9.5.1); Proteome Discoverer (ThermoScientific; v2.5) (PD 2.4); Cytoscape StringApp; MetaboAnalyst 5.0; Whole metaGenome Sequence Assembly pipeline, version 2 (WGSA2); MetaScape; Scalable Precision Medicine Open Knowledge Engine (SPOKE); QuPath (v0.4.3); Ilastik (v1.4.0); Syglass (v.1.7.2-79); Imaris (v10.0)

For manuscripts utilizing custom algorithms or software that are central to the research but not yet described in published literature, software must be made available to editors and reviewers. We strongly encourage code deposition in a community repository (e.g. GitHub). See the Nature Portfolio guidelines for submitting code & software for further information.

Data

Policy information about availability of data

All manuscripts must include a data availability statement. This statement should provide the following information, where applicable:

- Accession codes, unique identifiers, or web links for publicly available datasets
- A description of any restrictions on data availability
- For clinical datasets or third party data, please ensure that the statement adheres to our policy

Source data are provided with this paper.
Figure 1. A,B Aggregated from NASA OSDR (https://osdr.nasa.gov/bio/)

Figure 2. A,B and Supplementary Figure 1. A,B. The data included in these figures are available under restricted access and deidentified in order to preserve participants anonymity, access can be obtained by contacting Scott Smith (scott.m.smith@nasa.gov).

Fig. 3. A,B,C; Fig.7 B,C; Supplementary Fig. 6; Supplementary Data 1,8,9) underlying data from NASA OSDR (https://osdr.nasa.gov/bio/): OSD-462 (10.26030/8g1a-3041)

Fig 4. A; Fig5; Fig 6. A,B; Fig 8; Supplementary Fig. 2,4,5,7; Supplementary Data 2,3,6,7) underlying datasets from NASA OSDR (https://osdr.nasa.gov/bio/): OSD-102 (10.26030/yn9m-2d19), 163 (10.26030/q8vt-7p92), 253 (10.26030/4mx6-5x80), 336 (10.26030/qasa-rr29), 342 (10.26030/v2ak-0y21), 462 (10.26030/8g1a-3041), 513 (10.26030/pprb-6227), 457 (10.26030/yyce-8y73), 530 (10.26030/r2xr-h714), 532 (10.26030/j15f-vj38), 571 (10.26030/h3p5-tc29), 708 (10.26030/r4pv-hw21), 709 (10.26030/fhs1-z519)

Fig 4. B; Supplementary Fig.3,7; Supplementary data 4) underlying datasets from NASA OSDR (https://osdr.nasa.gov/bio/): OSD-72 (10.26030/qyw7-qn34), 212 (10.26030/8vac-wb94), 249 (10.26030/h713-bd02), 250 (10.26030/rj1y-dq03), 465 (10.26030/vqhs-qq63), 466 (10.26030/6axn-0058)

Fig 4. C; Supplementary Fig. 7; Supplementary Data 5) Rat data at request of CNSA authors (10.3389/fphys.2020.00939), LL MHU-3 data at request from authors (https://ibsls.megabank.tohoku.ac.jp/metabolite-list) a NASA OSDR (https://osdr.nasa.gov/bio/): OSD-571 (10.26030/h3p5-tc29)

Fig. 6C) data available from Peptide Atlas (https://peptideatlas.org/). Dataset identifier: PASS00239

Fig. 7A) kidney and bodyweight data are available on request to abehesht@broadinstitute.org for BNL-11213 mice and to valery.boyko@nasa.gov and NASA OSDR (https://osdr.nasa.gov/bio/): OSD-513 (10.26030/pprb-6227), 462 (10.26030/8g1a-3041), 710 (10.26030/r1hh-ev67), 712 (10.26030/f6b9-4093), 709 (10.26030/fhs1-z519)

Fig. 9B; Supplementary Fig. 9) underlying datasets from NASA OSDR (https://osdr.nasa.gov/bio/): OSD-710 (10.26030/r1hh-ev67) Fig 9D;

Fig 10A; Supplementary Data 12) underlying datasets from NASA OSDR (https://osdr.nasa.gov/bio/): OSD-708 (10.26030/r4pv-hw21) Fig. 10B) underlying datasets from NASA OSDR (https://osdr.nasa.gov/bio/): OSD- 706 (10.26030/rrwe-h429), 707 (10.26030/bcnk-5z50) Supplementary Fig. 1C) underlying datasets from NASA OSDR (https://osdr.nasa.gov/bio/): O5D-575 (10.26030/mc5d-p710) Supplementary

Fig. 7 Comprises all of the other data sets listed here. Supplementary Fig. 8; Supplementary Data 10,11) underlying datasets from NASA OSDR (https://osdr.nasa.gov/bio/): OSD-336 (10.26030/qasa-rr29)
For further information see Supplementary Information.

Research involving human participants, their data, or biological material

Policy information about studies with human participants or human data. See also policy information about sex, gender (identity/presentation), and sexual orientation and race, ethnicity and racism.

Reporting on sex and gender	As indicated in the text (See Fig 1B), the findings apply mostly male humans; due to the crew made up of the human crewed space flight missions. We are not at liberty to disaggregate the sex/gender data due to the onerous conditions of anonymity for the small number of astronauts and ethical concerns around identification.
Reporting on race, ethnicity, or other socially relevant groupings	We have not subclassified or studied different racial or ethnic groups in these studies, due to the onerous conditions of anonymity for the small number of astronauts and the ethical concerns around identification.
Population characteristics	We are unable to provide population characteristics due to the onerous conditions of anonymity for the small number of astronauts and the ethical concerns around identification.
Recruitment	Participants were recruited as part of the selection process for the relevant space missions. The details of which are not accessible to the general public in all instances.

Ethics oversight

Human studies ethical approval
All crews from the Inspiration4, NASA, JAXA spaceflights provided informed written consent prior to their participation. Inspiration4 subjects were consented at an informed consent briefing (ICB) at SpaceX (Hawthorne, CA), and samples were collected and processed under the approval of the Institutional Review Board (IRB) at Weill Cornell Medicine, under Protocol 21-05023569. All crew members have consented for data and sample sharing. Tissue samples were provided by SpaceX Inspiration4 crew members after consent for research use of the biopsies, swabs, and biological materials.
The procedure followed guidelines set by the Health Insurance Portability and Accountability Act (HIPAA) and operated under Institutional Review Board (IRB) approved protocols. Experiments were conducted in accordance with local regulations and with the approval of the IRB at Weill Cornell Medicine (IRB #21-05023569). NASA IRB (CSA defers to NASA's IRB), ESA IRB, JAXA IRB for their respective crewmembers from Biochemical Profile (NASA IRB Pro0797) and Nutritional Status Assessment: SMO 016E (pro0326) projects. All crews provided informed written consent prior to participation.
The JAXA human spaceflight study was proposed to and supported by the 2014 International Life Sciences Research Announcements, JAXA, and NASA. Ethics committee approvals were obtained at the University of Tsukuba (No. 251, Nov. 27, 2015), JAXA (JX-IRBA-20-071, Aug. 30, 2016), NASA (Pro1995, Feb. 28, 2017), ESA (2017_04_09, Apr. 20, 2017). Informed consent was obtained by the personal information manager of the study, and de-identified samples were made available to researchers who performed sample processing and data analysis.
Cosmonaut data were obtained from previously published data [https://link.springer.com/article/10.1007/s10517-013-2310-2]

Animal Studies ethical approval
For all animal data coming from NASA Genelab Open Science Data Repository (see detailed citations for all missions in the supplementary methods section) the ethical oversight information and protocol number can be found in the Protocol section (e.g.https://osdr.nasa.gov/bio/repo/data/studies/OSD-102).
For BNL-1/2/3 - Brookhaven National Laboratory IACUC Protocol 506 "miRNA Signature Detection and Countermeasures Against HZE Radiation Exposure for Tissue Degeneration".
For NSRL-22A - All care and procedures were approved by the Institutional Animal Care and Use Committees (IACUC) at BNL and CHOP and were in accordance with the AAALAC and National Institute of Health (NIH) guidelines for the care and use of laboratory animals. For RR-10- NASA John F. Kennedy Space Center Institutional Animal Care and Use Committee (IACUC) Research Protocol Review IACUC Protocol #: FLT-20-133 "The Role of CDKN1a/p21Pathway in Microgravity-Induced Bone Tissue Regenerative Arrest – A Spaceflight Study of Transgenic CDKN1a/p21-Null Mice in Microgravity (SpaceX-21)"

Note that full information on the approval of the study protocol must also be provided in the manuscript.

Field-specific reporting

Please select the one below that is the best fit for your research. If you are not sure, read the appropriate sections before making your selection.

[X] Life sciences [ ] Behavioural & social sciences [ ] Ecological, evolutionary & environmental sciences

For a reference copy of the document with all sections, see nature.com/documents/nr-reporting-summary-flat.pdf

Life sciences study design

All studies must disclose on these points even when the disclosure is negative.

Sample size

Due to the nature of our study, we used samples from animals that had been in spaceflight (usually as part of the 'Rodent Research' missions to the International Space Station) or had been taken from costly experiments using the Galactic Cosmic Radiation Simulator at Brookhaven national laboratories. We also used samples and/or data from humans who had undergone spaceflight (in historical NASA missions, Roscosmos missions, and JAXA missions to the ISS or modern commercial missions with SpaceX such as inspiration4). Our sample numbers were therefore limited to the number of samples made available to us in the original experimental designs or mission parameter limitations (restricted by practical considerations of weight to launch, available space and resource consumption). In all cases, we endeavored to use the maximum number of biological replicates available to us to maximise experimental power.

Data exclusions

Data were only excluded on technical grounds due to poor sample preservation quality where positive control measures or QC standards failed, or where the identity of samples matching to animals/participant ID number could not be validated and mislabeling was suspected.

Replication

All-omics studies were performed using a minimum of three technical & three biological replicates. All low-throughput studies (e.g. imaging, qPCR, morphometry) were reproduced in a minimum of 3 biological replicates. All attempt at replications were successful.

Randomization

Some data were unsuitable for randomisation i.e. the manned mission data (Inspiration, NASA, JAXA) which often formed their own controls; while other datasets were shipped to us with the samples already randomised. These were randomised by randomised block design.

Blinding

All experimental samples arrived to us blinded. However, the omics samples were unblinded during analysis, and samples from the manned missions were not blinded in that they formed their own controls.

Reporting for specific materials, systems and methods

We require information from authors about some types of materials, experimental systems and methods used in many studies. Here, indicate whether each material, system or method listed is relevant to your study. If you are not sure if a list item applies to your research, read the appropriate section before selecting a response.

Materials & experimental systems

n/a	Involved in the study
☐	☒ Antibodies
☒	☐ Eukaryotic cell lines
☒	☐ Palaeontology and archaeology
☐	☒ Animals and other organisms
☒	☐ Clinical data
☒	☐ Dual use research of concern
☒	☐ Plants

Methods

n/a	Involved in the study
☒	☐ ChIP-seq
☒	☐ Flow cytometry
☒	☐ MRI-based neuroimaging

Antibodies

Antibodies used

Primaries:
Target: anti-rat total NCC (tNCC) [cross reacts with mouse and human total NCC]
Host species: Rabbit
Clonality: pAb full IgG
Product: Abcam (ab95302)
Lot #: GR3274565-9
Working Concentration: @2ug/mL

Target: anti-human phospho-NCC (pNCC) Thr46, Thr50, Thr55 [cross reacts with mouse pNCC Thr44, Thr48, Thr53]
Host species: Sheep
Clonality: pAb IgG
Product: MRC PPU Reagents and Services (S908B)
Working Concentration: @2ug/mL (+ 10ug/mL non-phosphopeptide to ensure specificity)

Target: anti-human phospho-NCC (pNCC) Thr60 [cross reacts with mouse pNCC Thr58]
Host species: Sheep
Clonality: pAb full IgG
Product: MRC PPU Reagents and Services (S995B)
Lot #: 1st Bleed
Working Concentration: @2ug/mL (+ 10ug/mL non-phosphopeptide to ensure specificity)

Secondaries:
Target: Alexa Fluor 647 anti-rabbit IgG
Host species: Donkey
Clonality: pAb IgG Fab Fragment
Product: Jackson ImmunoResearch (711-547-003)
Working Concentration: @8ug/mL

Target: Alexa Fluor 555+ anti-rabbit IgG
Host species: Donkey
Clonality: pAb full IgG
Product: ThermoFisher (A32794)
Working Concentration: @10ug/mL

Target: Alexa Fluor 647+ anti-Goat IgG
Host species: Donkey
Clonality: pAb full IgG
Product: ThermoFisher (A32849)
Working Concentration: @10ug/mL

Validation

Primary:
1. Previously published in model that decreases NCC phosphorylation (https://doi.org/10.1093/hmg/ddv185)
2. Previously published in model that decreases NCC phosphorylation (https://doi.org/10.1093/hmg/ddv185)
3. Previously published in model that increase or decreases NCC phosphorylation (https://doi.org/10.1093/hmg/ddv185 and https://doi.org/10.15252/emmm.201505444)

These antibodies have been extensively used in the research communities and validated in both knockdown, overexpression and physiologically stimulated in vitro cellular systems [https://mrcppureagents.dundee.ac.uk/product/71984]. They have also been validated orthogonally by IHC and Western blot in mouse tissues, from models which have inhibition (Gitelman syndrome) or stimulation (Gordon syndrome) of NCC abundance and phosphorylation due to alterations in upstream regulators. The Abcam antibody (ab95302) is identical to the Stress Marq SPC-402 and Chemicon AB3553 antibodies, they are highly regarded in the scientific community, evidenced by over 100 citations collectively from the three suppliers. As well as by colocalisation with other anti-NCC antibodies targeting different protein regions and also in NCC knockout mouse kidney tissues. The Chemicon AB3553 antibody's validation is further demonstrated by its ability to stain NCC knockout (KO) mouse tissue, as shown on the Sigma-Aldrich website. The phospho-specific NCC antibody has been validated through experiments where human NCC was expressed and induced phosphorylation. This data is available on the Medical Research Council Protein Phosphorylation and Ubiquitination Unit (MRC-PPU) website: https://mrcppureagents.dundee.ac.uk/reagents-view-antibodies/588276
[DOI 10.1002/emmm.200900058] [DOI: 10.1093/hmg/ddv185] [DOI: 10.15252/emmm.201505444]

Secondary:
1. Extensive citations could be found at: https://www.jacksonimmuno.com/catalog/products/711-547-003
2. Extensive citations could be found at: https://www.thermofisher.com/antibody/product/Goat-anti-Rabbit-IgG-H-L-Highly-Cross-Adsorbed-Secondary-Antibody-Polyclonal/A32794
3. Extensive citations could be found at: https://www.thermofisher.com/antibody/product/Donkey-anti-Goat-IgG-H-L-Highly-Cross-Adsorbed-Secondary-Antibody-Polyclonal/A32849

Animals and other research organisms

Policy information about studies involving animals; ARRIVE guidelines recommended for reporting animal research, and Sex and Gender in Research

Laboratory animals	See Figure 1b for the sex and strain of the research animals. Ages were obtained from NASA Genelab Open Science Data Repository (OSDR). Details of housing conditions, ambient temperature and humidity were also available on the OSDR. Mice were quarantined and acclimated to a standard 12:12h light:dark cycle, with controlled temperature/humidity for 1-week prior to cage acclimation. Food and water were given ad libitum, and standard bedding was changed once per week.
Wild animals	No wild animals were used in this study
Reporting on sex	As indicated in the text (See Fig 1B), the findings apply mostly female animals (roughly two thirds of animal missions were female). As we were collecting data from previously conducted experiments we did not have the ability to alter this sex disparity. Only one study had both males and females, which enabled us to perform sex difference analyses for parameters such as blood and urine electrolytes.
Field-collected samples	No field-collected samples were used in this study
Ethics oversight	The ethics statement below is provided in the main manuscript.<br><br>For all animal data coming from NASA Genelab Open Science Data Repository (see detailed citations for all missions in the supplementary methods section) the ethical oversight information and protocol number can be found in the Protocol section (e.g. https://osdr.nasa.gov/bio/repo/data/studies/OSD-102).<br>For BNL-1/2/3 - Brookhaven National Laboratory IACUC Protocol 506 "miRNA Signature Detection and Countermeasures Against HZE Radiation Exposure for Tissue Degeneration".<br>For NSRL-22A - All care and procedures were approved by the Institutional Animal Care and Use Committees (IACUC) at BNL and CHOP and were in accordance with the AAALAC and National Institute of Health (NIH) guidelines for the care and use of laboratory animals.<br>For RR-10- NASA John F. Kennedy Space Center Institutional Animal Care and Use Committee (IACUC) Research Protocol Review IACUC Protocol #: FLT-20-133 "The Role of CDKN1a/p21Pathway in Microgravity-Induced Bone Tissue Regenerative Arrest – A Spaceflight Study of Transgenic CDKN1a/p21-Null Mice in Microgravity (SpaceX-21)"

Note that full information on the approval of the study protocol must also be provided in the manuscript.

This checklist template is licensed under a Creative Commons Attribution 4.0 International License, which permits use, sharing, adaptation, distribution and reproduction in any medium or format, as long as you give appropriate credit to the original author(s) and the source, provide a link to the Creative Commons license, and indicate if changes were made. The images or other third party material in this article are included in the article's Creative Commons license, unless indicated otherwise in a credit line to the material. If material is not included in the article's Creative Commons license and your intended use is not permitted by statutory regulation or exceeds the permitted use, you will need to obtain permission directly from the copyright holder. To view a copy of this license, visit http://creativecommons.org/licenses/by/4.0/
